# *In Crystallo* Wolff Rearrangement of a Metalated Diazoester: Structural Confirmation of the Singlet Carbene Wolff-Intermediate

**DOI:** 10.1021/jacs.4c18289

**Published:** 2025-02-11

**Authors:** Ze-Jie Lv, Arnd Fitterer, Regine Herbst-Irmer, Serhiy Demeshko, Hendrik Verplancke, Max C. Holthausen, Sven Schneider

**Affiliations:** † Institut für Anorganische Chemie and International Center for Advanced Studies of Energy Conversion (ICASEC), University of Göttingen, Tammannstraße 4, 37077 Göttingen, Germany; # Institut für Anorganische und Analytische Chemie, Goethe-Universität, Max-von-Laue-Straße 7, 60438 Frankfurt am Main, Germany

## Abstract

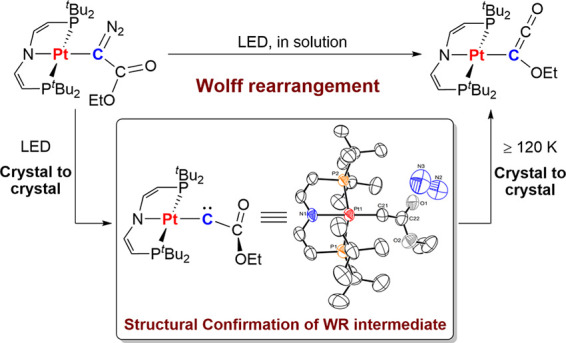

The Wolff rearrangement (WR) is widely used for the synthesis of ketenes from diazoketones and -esters. Stepwise WR reactions are proposed to proceed through transient carbonylcarbene (R–C–C(O)–R′) intermediates, which so far have evaded structural characterization. Here, a Wolff metallocarbene (Pt^II^–C–C(O)–OEt) is reported as a fleeting intermediate in the photoinitiated fragmentation of a diazoester ligand. Frozen solution and crystal matrix isolation experiments enabled the spectroscopic, magnetic, crystallographic, and computational characterization of this highly reactive species. All methods confirmed a singlet ground state for the WR metallocarbene, which is stabilized by π interactions with the carboxyl substituent, thus complementing computational and transient spectroscopy studies for classic organic WR reactions.

The Wolff rearrangement (WR) is a key methodology for the selective rearrangement of carbon frameworks, as in the Arndt-Eistert homologation or ring contraction strategies.^1−8^ Thermal or photochemical N_2_ elimination from α-diazoketone or -ester precursors and 1,2-shift of substituent R′ (Figure 1a) give ketenes as versatile synthetic building blocks. The WR was examined by matrix isolation, transient spectroscopy, and computational studies, and the degree of concertedness was subject of debate.^2,9−14^ Early work related the mechanism to stereochemical arguments: *syn*-coplanar orientation of the diazo and carbonyl groups, as in cyclic examples, favors a concerted path while the *anti* conformer favors stepwise mechanisms via a carbonylcarbene intermediate (Figure 1a).^15,16^ Time-resolved spectroscopy later revealed a more complex picture. Both paths can contribute;^11^ the stepwise mechanism generally benefits from carbene stabilization by substituents with low propensity for 1,2-migration, σ interactions with solvents, and/or π interactions with the carbonyl group. The latter requires orthogonal orientation of the carbene and carbonyl moieties. However, the transient nature of WR carbenes so far prevented crystallographic confirmation, contrasting with the plethora of isolable singlet carbenes.^17^

**Figure 1:**
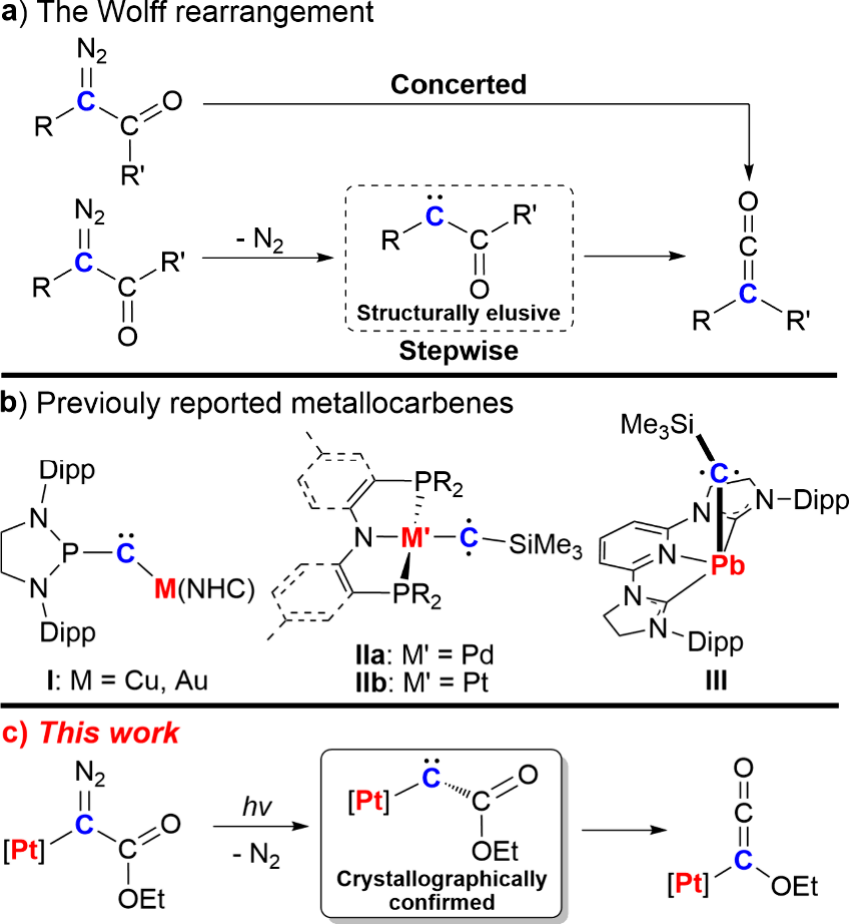
(a) Mechanism of the WR. (b) Metallocarbenes reported in the literature. (c) Metallo-WR reported here.

The Liu group reported the first isolable carbenes with transition-metal substituents (Figure 1b, **I**). The coinage metallocarbenes favor M–C single bonding over a carbyne (M≡C–R) structure and singlet ground states that are stabilized by unidirectional π-push phosphino substituents.^18−20^ In contrast, our group 10 triplet metallocarbenes **IIa/b** rely on spin-polarized α-pull and β-push donor/acceptor π interactions that do not break p-orbital degeneracy at the carbon atom.^21,22^ Munz and co-workers recently reported a related plumbacarbene (Figure 1b, **III**) that connects chemical bonding for main group and transition-metallocarbenes.^23^*In crystallo* generation of **IIa**/**b** and other highly reactive nitrenoid and carbenoid species proved instrumental for structural characterization.^24,25^ Spurred by this precedence, we set out to examine fleeting WR metallocarbenes in the absence of potentially interacting solvents (Figure 1c).

Diazoesters were chosen as precursors over diazoketones due to lower tendency for alkoxy 1,2-shift and, thus, higher quantum yields toward WR carbenes.^4^ Analytically pure [Pt{C(N_2_)CO_2_Et}(PNP)] (**1**, PNP = N(CHCHP^*t*^Bu_2_)_2_) was obtained in moderate yields (32%) upon *in situ* deprotonation of diazoethyl acetate and salt metathesis with the respective platinum(II) triflate complex. Assignment of two distinct IR bands (ν̃ = 2031 and 1664 cm^–1^ in THF) to the diazo and carbonyl stretching modes of **1** was confirmed by DFT (ν̃_BP86_ = 2057 and 1587 cm^–1^) and comparison with reported metalated diazoesters.^26^ X-ray crystallography revealed coplanar diazo and carbonyl groups with mutual *s*-*cis* conformation (*vide infra*).^27−30^ The UV/vis spectrum features dominant absorptions in the near-UV region and a weak visible band at λ_max_ = 467 nm (ε = 360 M^–1^ cm^–1^). TD-DFT computations excellently reproduced this transition (λ_TD-DFT_ = 462 nm), which exhibits π_⊥_^*^ → π_∥_^*^ (HOMO–1 → LUMO) character.

Bulk photolysis of **1** in toluene or THF was carried out with a blue light-emitting diode (LED; λ_max_ = 467 ± 20 nm) at −40 °C. The solution initially changed color from light yellow to light violet but ultimately faded to colorless over the course of 1 h. Workup afforded the formyl complex [Pt(CHO)(PNP)] (**2**) (Figure 2a) in 71% yield. Photofragmentation of the diazoester to a formyl ligand is supported by a characteristic ^1^H NMR triplet signal at δ = 17.0 ppm (^3^*J*_P–H_ = 4.9 Hz) with ^195^Pt satellites (^2^*J*_Pt–H_ = 165 Hz). The C=O stretching mode can be assigned to the IR band at ν̃_CHO_ = 1595 cm^–1^, as supported by DFT calculations (ν̃_BP86_ = 1584 cm^–1^) and comparison with other Pt^II^ formyl complexes.^31^ Single-crystal X-ray crystallography (SC-XRD) confirmed the molecular structure of **2** (Figure 2b).

**Figure 2:**
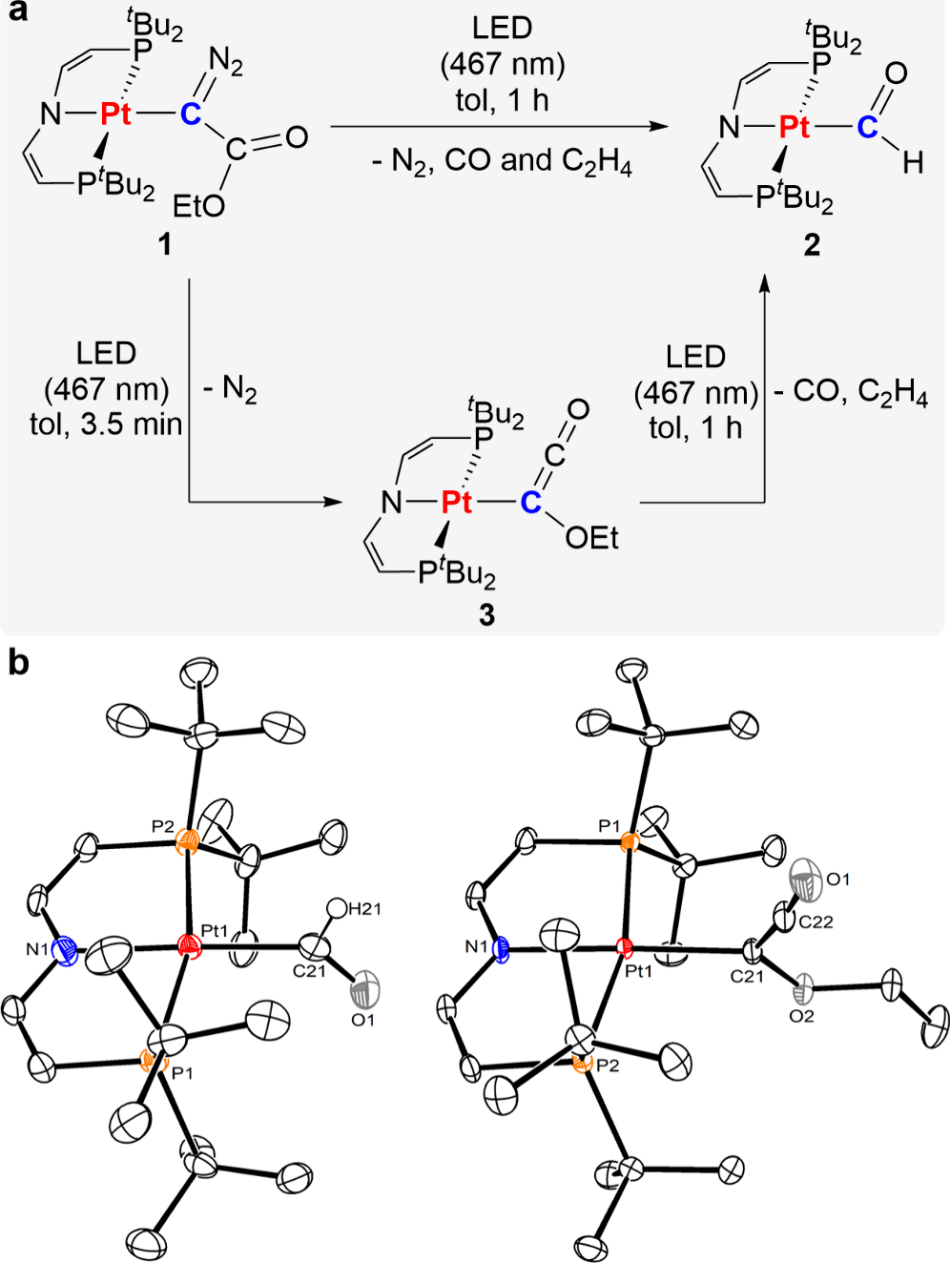
Solution syntheses and molecular structures of **2** and **3** (thermal ellipsoids at 50% probability; H atoms, except C*H*O in **2**, omitted for clarity).

Besides **2**, ethylene was detected (^1^H NMR), providing a lead toward the photofragmentation mechanism. Next, photolysis was reduced to a few minutes to identify the violet intermediate (**3**), which could be isolated in 65% yield (Figure 2a). The IR spectrum of **3** revealed a strong band at 2023 cm^–1^. Comparison with [Pd{C(CO)SiMe_3_}(PNP)] (ν̃_C=C=O_ = 2006 cm^–1^)^21^ and other reported ketenyl complexes^32^ suggests initial photoconversion of the diazoester to a ketenyl ligand. DFT computations (ν̃_BP86_ = 2018 cm^–1^) and SC-XRD (Figure 2b) confirmed the structural assignment of intermediate **3** as the η^1^-κ_C_-ethylketenyl complex [Pt{C(CO)OEt}(PNP)].

Ketenyl complexes of main group and transition metals are well-known, e.g., from CO addition to carbyne complexes, metallocarbenes, or ylides.^32,33^ Direct formation from a metallodiazoester represents a new route, suggesting a WR as initial step of the photofragmentation sequence. Accordingly, bulk photolysis of **3** under identical conditions selectively gave the final formyl complex **2** and ethylene. Notably, the high WR selectivity contrasts with many organic diazoesters, which exhibit overstabilized WR carbene intermediates due to large ester resonance energy.

The thermal steps of the reaction sequence were further evaluated by ONIOM(CCSD(T)-F12:r^2^SCAN-3c) computations (Figure 3). N_2_ loss from precursor **1** gives an ethylcarboxy metallocarbene (**4**) with a singlet ground state that is moderately stabilized with respect to the triplet electromer (Δ*G*_S/T_ = 5.9 kcal·mol^–1^). Highly exergonic 1,2-migration of the methoxy group (Δ*G*° = −20.0 kcal·mol^–1^) proceeds with a small kinetic barrier (Δ*G*^⧧^ = 10.6 kcal·mol^–1^). Photolytic CO dissociation again produces a metallocarbene (**5**), yet with a large singlet/triplet gap as expected for an alkoxycarbene (Δ*G*_S/T_ = 23.7 kcal·mol^–1^; see the Supporting Information for a full quantum chemical characterization).^34^ Ethylene extrusion via γ-H transfer gives the final formyl product **2** in a strongly exergonic step (Δ*G*° = −41.2 kcal·mol^–1^). Notably, the computed barrier (Δ*G*^⧧^ = 10.4 kcal·mol^–1^) is almost identical with that of the WR. We thus attribute the ability to isolate the ketene complex at short photolysis times to the significantly higher quantum yield for N_2_ photoelimination from **1** (31 ± 5%) vs photoextrusion of CO from the ketenyl intermediate **3** (9 ± 2%).

**Figure 3:**
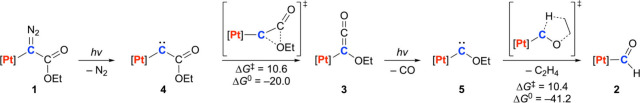
Computed pathway for the transformation of **1** to **2** (free energies in kcal mol^–1^, ONIOM(CCSD(T)-F12:r^2^SCAN-3c) results).

This mechanistic proposal requires that the photochemical steps produce metallocarbene photoproducts with sufficient lifetime for electronic and thermal relaxation. We therefore evaluated the thermal stability of the WR intermediate **4**.^35^ Photolysis of **1** in 2-methyl-THF glass at 77 K resulted in a color change from yellow to green due to bleach of the weak band at 467 nm and evolution of the strong absorption at λ_max_ = 395 nm that can be assigned to the **4** (Figure 4). TD-DFT results are in excellent agreement with the HOMO→LUMO+1 transition of the singlet metallocarbene (λ_TD-DFT_ = 383 nm) with LP_C_ → σ* character. Warming beyond 130 K results in decay of the UV/vis signature of **4** along with formation of the final WR product **3**.

**Figure 4:**
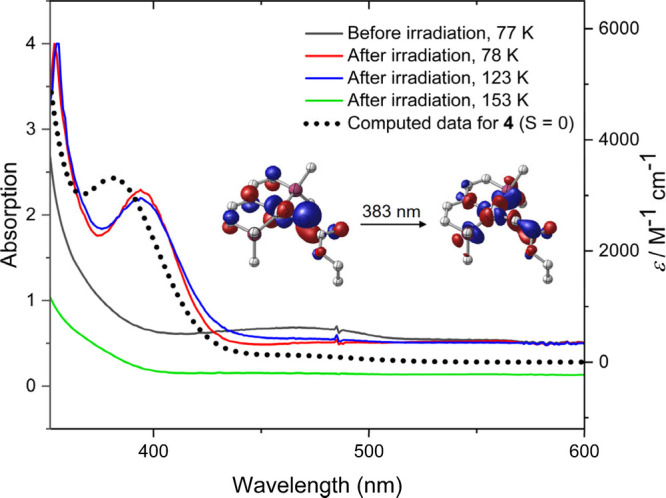
VT-UV/vis examination of the photolysis of **1** in 2-methyl-THF and computed absorption spectrum of **4**. Inset: Natural transition orbitals representing (96.1%) the absorption band of **4** at 383 nm (isosurfaces at ±0.05 a_0_^–3/2^, methyl groups and H atoms not shown).

Computations by Platz and co-workers on the WR intermediate *p*-biphenylmethylcarboxycarbene predicted a minute S/T-gap and a solvent-dependent ground state spin multiplicity.^13b^ We therefore set out to further evaluate the (electronic) structure of **4** in the absence of solvent by magnetometry and crystallography.^21,36^*In situ* photolysis of solid **1** at 10 K (λ_exc_ = 467 nm) inside a SQUID (superconducting quantum interference device) showed no change of the d.c. magnetic moment during the entire reaction period (see the Supporting Information for details). This observation supports the singlet ground state assignment from UV/vis spectroscopy and computations.

Structural characterization of **4** was carried out by continuous *in situ* photolysis (*λ*_exc_ = 530 nm) of a mounted single crystal of **1** inside a diffractometer at 80 K. Reaction progress was evidenced by gradual color change to green (Figure 5b) and monitored by periodic collection of diffraction data. Satisfactory crystallographic data was obtained for a crystal with 64% photoconversion as determined by structural refinement (see the Supporting Information). Refinement of the diffraction data revealed a decrease of the occupancy of the diazo nitrogen atoms. In turn, free N_2_ is observed within the crystalline matrix at a nonbonding distance of over 3.2 Å from the Pt–C moiety. Warming the photogenerated single crystal of **4** from 80 to 120 K resulted in a color change over several hours from green to light violet (Figure 5b). X-ray diffraction confirmed clean conversion of **4** to **3**,^37^ completing the full two-step *in crystallo* WR sequence and thus connecting the crystal matrix isolation results with solution reactivity.

**Figure 5:**
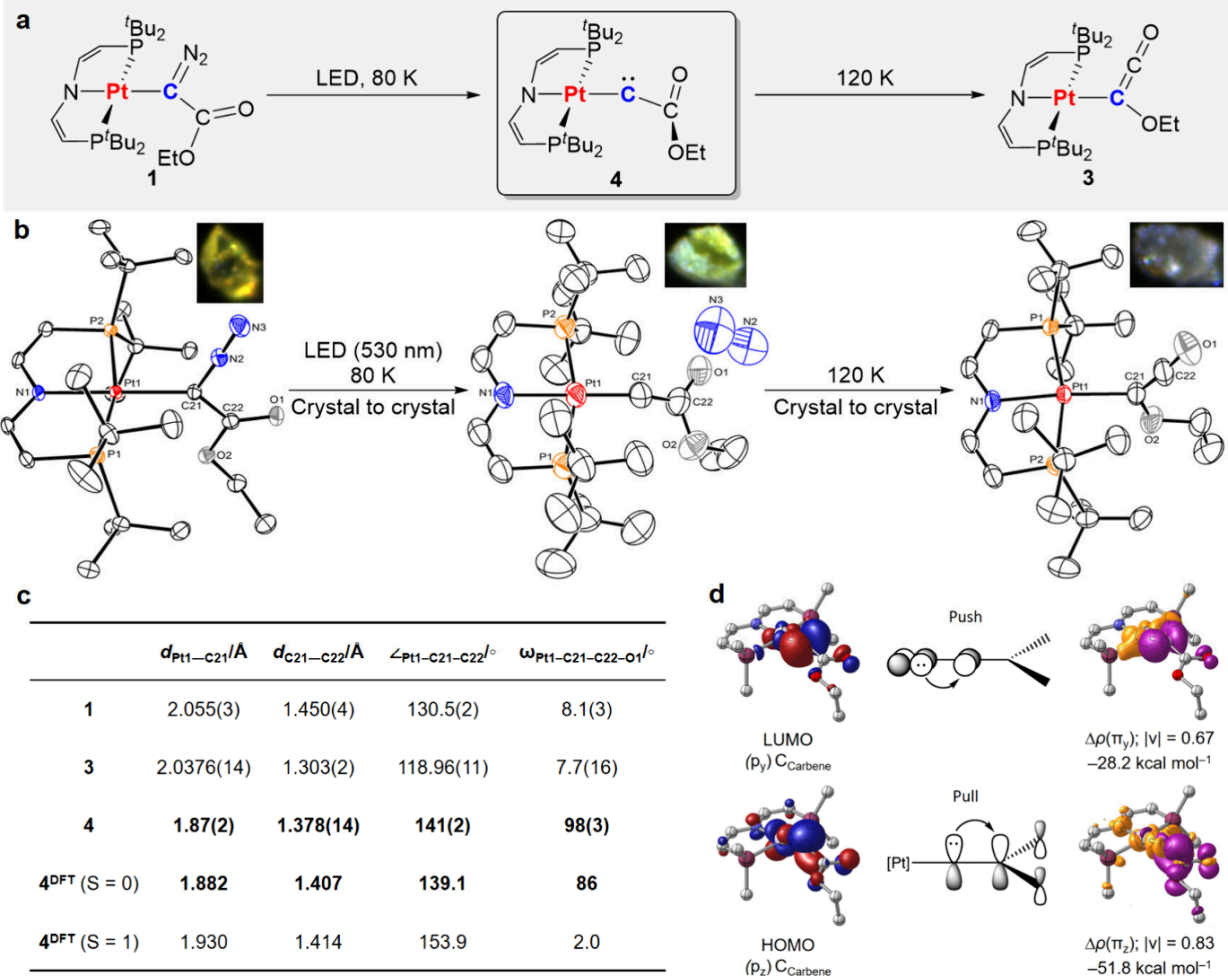
(a) *In crystallo* photo-WR of complex **1**. (b) Molecular structures (50% ellipsoid probability) of **1** as well as **4** and **3** obtained by crystal-to-crystal conversion;^37^ cocrystallized pentane and hydrogen atoms have been omitted for clarity. (c) Comparison of key structural parameters of **1**, **3** and **4** and DFT optimized bond parameters of ^**S**^**4** (*S* = 0) and ^**T**^**4** (*S* = 1). (d) Electronic structure analysis of the singlet ground state of ^**S**^**4**: frontier molecular orbitals (isovalue ±0.05 *a*_0_^–3/2^; H atoms and methyl groups not shown); schematic representation of the push–pull stabilization in the singlet ground state along with the corresponding natural orbital for chemical valence (NOCV) deformation densities (isovalues ±0.002 *a*_0_^–3^, charge flow orange → purple; |ν|: electron density transferred, energy contributions).

Several structural features of WR metallocarbene **4** support a singlet ground state, even if taking into account bond length and angle inaccuracies that result from disordered cocrystallization of **4** and **1**:

(a) The Pt–C bond distance of **4** (1.87(2) Å) is markedly shorter compared to **1** (2.055(3) Å) and ketenyl isomerization product **3** (2.0376(14) Å). It is also slightly shorter than that of triplet metallocarbene **IIb** (1.95(4) Å; Figure 1b),^21^ but still within the Pt–C single bond range as judged by Pyykkö’s covalent radii (*d*_Pt–C_ = 1.98 Å, *d*_Pt=C_ = 1.79 Å, *d*_Pt≡C_ = 1.70 Å).^38^ DFT structure optimizations excellently reproduced the Pt–C distance for the singlet state of **4** (1.882 Å), while a notably longer bond results for the triplet electromer (1.930 Å; Figure 5c).^39^
(b) Upon N_2_ loss, the bond angle ∠_Pt1–C21–C22_ around the carbene carbon atom slightly relaxes (**4**, 141(2)°; **1**, 130.5(2)°). However, the triplet carbenes **IIa** (∠_Pd–C–Si_ = 164.9(13)°) and **IIb** (∠_Pt–C–Si_ = 155.5(16)°) adopt much more linear structures to facilitate π interactions of both singly filled carbon p orbitals.^21^ DFT computations nicely reproduced the Pt–C–C angle for singlet **4** (139.1°), while a significantly more obtuse angle resulted for the triplet state (153.9°).
(c) The C_carbene_–C_CO_2_R_ distance shortens from 1.450(4) (**1**) to 1.378(14) Å (**4**). The increased double bond character suggests mixing of the carbene lone pair with the neighboring CO_2_R π* orbital, as expressed by the Wiberg bond index (1.33) and distinct conformational reorganization from **1** via **4** to **3**: While parent **1** features coplanar orientation of the diazo and the carboxyl group (ω_Pt–C–C–O_ = 8.1(3)°), almost perfectly perpendicular arrangement of the planes defined by the carbene (Pt–C–C) and carboxyl groups (ω_Pt–C–C–O_ = 98(3)°) is observed for **4**, as a prerequisite to efficient π bonding. In turn, a coplanar orientation is restored in the ketene isomer **3** (ω_Pt–C–C–O_ = 7.7(16)°), suggesting that crystal packing effects play a minor role for the conformational reorganization. Accordingly, space-filling crystallographic models of **1**, **3**, and **4** show no close contacts of the ethoxy group with other moieties, including free N_2_ (Figure S29). Computational work for organic Wolff carbonylcarbenes predicted orthogonal carbene/carboxy orientation for singlet, but coplanar arrangements for triplet minimum structures.^14a^ The DFT optimized structures for both electromers of **4** corroborate these results. The computed conformation for ^**S**^**4** is close to experiment (ω_Pt–C–C–O_ = 86°) vs a coplanar structure for ^**T**^**4** (ω_Pt–C–C–O_ = 2°); a rotational barrier of Δ*G*^⧧^ = 12 kcal·mol^–1^ computed on the (broken-symmetry) singlet potential energy surface indicates that ^**S**^**4** resides in a rather steep potential. Quantum-chemical bonding analysis of ^**S**^**4** further revealed strong and mutually orthogonal Pt→C_Carbene_ push and C_Carbene_→C_CO_2_R_ pull π interactions (Figure 5d), thus stabilizing the singlet ground state.

In summary, we have reported the photofragmentation of metalated diazoester **1** to formyl complex **2**. The reaction sequence is comprised of an initial photo-WR and subsequent, light-induced CO loss from the ketene WR product, resulting in ethylene extrusion from the ethoxy substituent. Both steps proceed through metallocarbene intermediates. Crystal matrix isolation experiments of the first step allowed for full crystallographic resolution of a stepwise WR mechanism. The absence of potentially interacting solvent allowed for focusing on the stereoelectronic key parameters. Spectroscopic, magnetic, crystallographic, and computational data confirmed a singlet ground state for the metallocarbene Wolff intermediate **4** and confirmed singlet vs triplet stabilization upon rotation of the carbonyl group, as was previously proposed computationally. This study, thus, demonstrates that crystal matrix isolation of highly reactive species provides a powerful tool for mechanistic analysis of classic reactions like the WR.

## Supporting Information Available

Experimental and calculation details and NMR spectra of new compounds (PDF)
