# Supplementary material for: In Crystallo Wolff Rearrangement of a Metalated Diazoester: Structural Confirmation of the Singlet Carbene Wolff-Intermediate
Source: J Am Chem Soc. 2025 Feb 11;147(7):5590–5. doi: 10.1021/jacs.4c18289 (PMC11848915; doi:10.1021/jacs.4c18289)
Supplement: Supplementary file 1 — ja4c18289_si_001.pdf [file ja4c18289_si_001.pdf]

Cover Page for Supporting Information

Manuscript Title:

In Crystallo Wolff Rearrangement of a Metalated Diazoester: Structural Confirmation of the Singlet Carbene Wolff-Intermediate

Authors:

Ze-Jie Lv,[†,‡] Arnd Fitterer,[#,‡] Regine Herbst-Irmer,[†] Serhiy Demeshko,[†] Hendrik Verplancke,[#] Max C. Holthausen,[*,#] and Sven Schneider[*,†]

Affiliations:

[†] Institut für Anorganische Chemie, University of Göttingen, Tammannstraße 4, 37077 Göttingen, Germany

[#] Institut für Anorganische und Analytische Chemie, Goethe-Universität, Max-von-Laue-Strasse 7, 60438 Frankfurt am Main, Germany

[‡] These authors contributed equally.

Contents:

1. Experimental Details and Characterization Data **S3**

1.1 Materials and Methods S3

1.2 Analytical Methods S3

1.3 Syntheses and Characterization Data S4

1.4 Spectroscopic Characterization S7

1.5 Determination of Quantum Yield S18

2. Crystallographic Details **S19**

2.1 General Details S19

2.2 Crystal-to-crystal Transformation Experiments S20

2.3 Crystallographic Details of **1** S22

2.4 Crystallographic Details of **2** S26

2.5 Crystallographic Details of **3** S29

2.6 Crystallographic Details of **4** S32

2.7 Crystallographic Details of **3** from crystal-to-crystal transformation S37

3. *In situ* SQUID Measurement **S41**

4. Low-Temperature UV/Vis Spectra Measurement **S42**

5. Computational Details **S43**

5.1 Computational Methods S43

5.2 Molecular Structures of **1**, **2**, **3**, **4** and **5** S46

5.3 Calculated IR Spectra of **1**, **2** and **3** S48

5.4 Calculated UV/VIS Spectra of **1**, **2**, **3**, **4** and **5** S50

5.5 Singlet-Triplet Energy Differences for **4** and **5** S55

5.6 Characterization of Metallocarbene **4** S56

5.7 Characterization of Metallocarbene **5** S63

5.8 Computed Reaction Path S67

5.9 Total Energies S68

5.10 Cartesian Coordinates r^2SCAN-3c Molecular Structures (Å) S70

6. Supplementary References **S93**

1. Experimental Details and Characterization Data

1.1 Materials and Synthetic Methods

[(PNP)Pt(OTf)] (PNP = N(CHCHP^{t}Bu$_2$)$_2$) was synthesized according to published procedures.[1] Unless otherwise noted, all other starting materials were commercially available and were used without further purification. Solvents were purified by MBraun SPS-800 Solvent Purification System and dried over fresh Na chips and molecular sieves in a glovebox. Deuterated solvents were obtained from Deutero GmbH and dried over Na/K (C_6D_6, THF-d_8), distilled by trap-to-trap transfer in vacuo, and degassed by three freeze-pump-thaw cycles, respectively. All reactions were operated under an argon atmosphere in MBraun gloveboxes or under slightly positive dry argon pressure using standard Schlenk line techniques. The glove box atmosphere was constantly circulated through a copper/molecular sieves catalyst unit. Oxygen and moisture concentrations were monitored by an O_2/H_2O Combi-Analyzer to ensure that both were always below 0.1 ppm.

A 467 nm LED lamp (Kessil Lighting PR160L LEDs) was used for photochemical reactions in solution. Samples were placed in an ^{i}PrOH bath with cryogenic cooling circulation pump during photolysis. Prizmatix LEDs (PRI FC5-LED-USB-WL, Wavelengths: 365 nm (365A), 395 nm (395A), 420 nm (420Z), 455 nm (455Z), 530 nm (530TR)) coupled to fiber optic (polymer fiber, NA 0.5, diameter 1000 Mikrometer) were used for photolytic crystal-to-crystal conversion experiments.

1.2 Analytical Methods

NMR spectra were recorded on Bruker Avance III 300 or Avance III 400 spectrometers or an Avance 500 spectrometer equipped with Prodigy broadband cryoprobe, respectively. Spectra were calibrated to the residual solvent signals (C_6D_6: δ_H = 7.16 ppm, δ_C = 128.06 ppm; THF-d_8: δ_H = 1.73 ppm, δ_C = 25.37 ppm). IR spectra were obtained as powder on a Bruker ALPHA FT-IR spectrometer with Platinum ATR module. UV/vis spectra were recorded on an Agilent Cary 8454 spectrometer using quartz cuvettes and air tight caps. Elemental analyses were obtained by the Analytisches Labor, Georg-August-Universität using an Elementar Vario EL 3 analyzer. HR-ESI-MS (Bruker maXis QTOF) and LIFDI-MS (JEOL AccuTOF JMS-T100GCV; inert conditions) were measured by the Zentrale Massenabteilung, Fakultät für Chemie, Georg-August-Universität, Göttingen.

1.3 Syntheses and characterization data

[Pt{C(N_2)CO_2Et}(PNP)] (1)

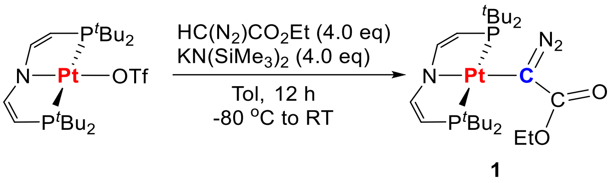

[Pt(OTf)(PNP)] (21.1 mg, 0.03 mmol) and K[N($SiMe_3$)$_2$] (23.8 mg, 0.12 mmol) were dissolved in toluene (*ca.* 5 mL) in a glovebox and the solution was cooled to –80 °C. Ethyl diazoacetate (15% in toluene, 102 µL, 0.12 mmol) was added and the reaction mixture was stirred at room temperature for 12 hours. After removal of the solvent, the residue was extracted with pentane (*ca.* 10 mL). After filtration, the volatiles were removed under reduced pressure. The residue was again extracted with pentane (*ca.* 2 mL) and filtered. After filtration, the volatiles were removed to give a yellow powder. Analytically pure **1** (6.4 mg, 32% yield) was obtained by crystallization from a pentane solution (*ca.* 1 mL) at −35 °C over one week, which also provided single crystals suitable for X-ray diffraction. ^{1}H NMR (300 MHz, C_6D_6, 298 K): δ (ppm) 6.90 ($A_{18}XX'A'_{18}$, N = | $^3J_{HP} + ^4J_{HP}$ | = 37.6 Hz, $^3J_{HH}$ = 5.6 Hz, $^3J_{PtH}$ = 59.6 Hz, 2H, NC*H*), 4.25 (q, $^3J_{HH}$ = 7.1 Hz, 2H, O*CH*$_2$CH$_3$), 4.05 ($A_{18}XX'A'_{18}$, N = |$^2J_{HP} + ^4J_{HP}$| = 9.8 Hz, $^3J_{HH}$ = 5.6 Hz, $^3J_{PtH}$ = 29.0 Hz, 2H, PC*H*), 1.43–1.23 (m, 36H, C*Me*$_3$), 1.15 (t, $^3J_{HH}$ = 7.1 Hz, 3H, OCH$_2$*CH*$_3$). ^{13}C{^{1}H} NMR (126 MHz, C_6D_6, 298 K): δ (ppm) 172.6 (s, *C*O$_2$Et), 162.3 (t, $^2J_{PC}$ = 7.2 Hz, $^2J_{PtC}$ = 68.6 Hz, N*C*H), 83.8 (t, $^1J_{PC}$ = 21.4 Hz, $^2J_{PtC}$ = 34.2 Hz, *C*HP), 59.7 (s, *C*H$_2$CH$_3$), 36.7 (vt, $^1J_{PC}$ = 14.6 Hz, P*C*Me$_3$), 35.6 (vt, $^1J_{PC}$ = 12.9 Hz, P*C*Me$_3$), 29.2 (br, PC*Me*$_3$), 29.1 (br, PC*Me*$_3$), 15.1 (br, CH$_2$*C*H$_3$), 15.1 (br, $^1J_{PtC}$ = 904.2 Hz, Pt*C*(N$_2$), this peak overlapped with the CH$_2$*C*H$_3$ peak). ^{31}P{^{1}H} NMR (121 MHz, C_6D_6, 298 K): δ (ppm) 63.0 (s, $^1J_{PtP}$ = 2648.8 Hz). IR (ATR): 2026 (CN$_2$), 1643 (C=O), 1529 (C=C). IR (in THF): 2031 (1067, CN$_2$), 1664 (632, C=O), 1536 (2264, C=C). UV/Vis (THF): λ_{max} [nm] (ε [$M^{-1}cm^{-1}$]) = 254 (16500), 313 (23300), 467 (360). HRMS (m/z): $[M+H]^+$ calcd. for $[C_{24}H_{45}N_3O_2P_2Pt+H]^+$: 665.2710; found, 665.2698. Anal. Calcd for $C_{24}H_{45}N_3O_2P_2Pt$: C, 43.37; H, 6.82; N, 6.32. Found: C, 43.53; H, 6.89; N, 5.95.

[Pt(CHO)(PNP)] (2)

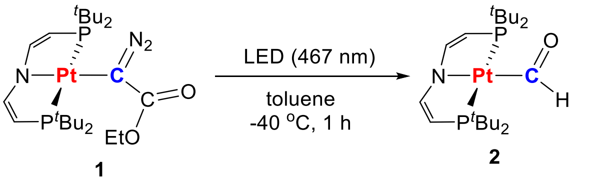

Complex **1** (6.7 mg, 0.010 mmol) was placed in a J. Young NMR tube and dissolved in toluene-

d_8 (*ca.* 0.4 ml). The solution was photolyzed with a 467 nm LED at –40 °C for 1 hour. After removal of the solvent, the residue was extracted with 3 mL pentane. Filtration and removal of the volatiles gave **2** (4.1 mg, 71% yield) as a colourless powder of analytical purity. Suitable single crystals of **2** for X-ray analysis could be acquired by crystallization of a saturated pentane solution at −35 °C for 1 week. ^{1}H NMR (300 MHz, C_6D_6, 298 K): δ (ppm) 17.00 (t, $^2J_{PC}$ = 4.9 Hz, $^1J_{PtC}$ = 164.8 Hz, PtC*H*O), 7.04 ($A_{18}XX'A'_{18}$, N = | $^3J_{HP}$ + $^4J_{HP}$ | = 18.5 Hz, $^3J_{HH}$ = 5.0 Hz, $^3J_{PtH}$ = 32.8 Hz, 2H, NC*H*), 4.05 ($A_{18}XX'A'_{18}$, N = |$^2J_{HP}$ + $^4J_{HP}$| = 7.3 Hz, $^3J_{HH}$ = 5.0 Hz, $^3J_{PtH}$ = 35.0 Hz, 2H, PC*H*), 1.26 ($A_{18}XX'A'_{18}$, N = |$^3J_{HP}$ + $^5J_{HP}$| = 14.3 Hz, 36H, C*Me*$_3$). ^{13}C{^{1}H} NMR (101 MHz, C_6D_6, 298 K): δ (ppm) 228.9 (t, $^2J_{PC}$ = 8.6 Hz, $^1J_{PtC}$ = 920.8 Hz, Pt*C*HO), 161.8 (t, $^2J_{PC}$ = 7.4 Hz, $^2J_{PtC}$ = 68.7 Hz, N*C*H), 82.7 (t, $^1J_{PC}$ = 23.9 Hz, $^2J_{PtC}$ = 33.4 Hz, *C*HP), 59.7 (s, *C*H$_2$CH$_3$), 36.7 (vt, $^1J_{PC}$ = 13.8 Hz, P*C*Me$_3$), 29.2 (t, $^1J_{PC}$ = 3.2 Hz, PC*Me*$_3$). ^{31}P{^{1}H} NMR (121 MHz, C_6D_6, 298 K): δ (ppm) 66.6 (s, $^1J_{PtP}$ = 2971.2 Hz). IR (ATR): 1595 (C=O), 1514 (C=C). UV/Vis (THF): λ_{max} [nm] (ε [M^{-1}cm^{-1}]) = 324 (12200). HRMS (m/z): [M+H]$^+$ calcd. for [$C_{21}H_{41}NOP_2Pt$+H]$^+$: 581.2263; found, 581.2283. Anal. Calcd for $C_{21}H_{41}NOP_2Pt$: C, 43.44; H, 7.12; N, 2.41. Found: C, 43.53; H, 7.09; N, 2.26.

[Pd{C(CO)OEt}(PNP)] (3)

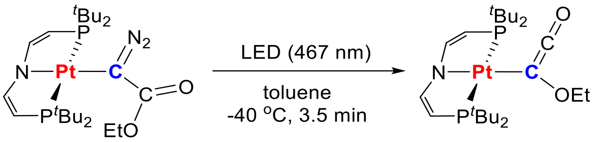

Complex **1** (6.6 mg, 0.010 mmol) was added into a J. Young NMR tube and dissolved in toluene-d_8 (*ca.* 0.5 ml). The solution was photolyzed with a 467 nm LED at –40 °C for 3.5 minutes. After removal of the solvent, a violet powder was obtained. Analytically pure **3** (4.1 mg, 65% yield) was obtained by crystallization from a pentane solution (*ca.* 1 mL) at −35 °C over one week, which also provided single crystals suitable for X-ray diffraction. ^{1}H NMR (300 MHz, C_6D_6, 298 K): δ (ppm) 7.01 ($A_{18}XX'A'_{18}$, N = | $^3J_{HP}$ + $^4J_{HP}$ | = 19.4 Hz, $^3J_{HH}$ = 5.6 Hz, $^3J_{PtH}$ = 37.2 Hz, 2H, NC*H*), 4.11 ($A_{18}XX'A'_{18}$, N = |$^2J_{HP}$ + $^4J_{HP}$| = 7.3 Hz, $^3J_{HH}$ = 5.6 Hz, $^3J_{PtH}$ = 29.4 Hz, 2H, PC*H*), 3.50 (q, $^3J_{HH}$ = 7.0 Hz, 2H, OC*H*$_2$CH$_3$), 1.36 ($A_{18}XX'A'_{18}$, N = |$^3J_{HP}$ + $^5J_{HP}$| = 14.2 Hz, 36H, C*Me*$_3$), 1.18 (t, $^3J_{HH}$ = 7.0 Hz, 2H, OCH$_2$*CH*$_3$). ^{13}C{^{1}H} NMR (126 MHz, C_6D_6, 298 K): δ (ppm) 222.5 (s, $^2J_{PtC}$ = 96.9 Hz, PtC*C*O), 162.1 (t, $^2J_{PC}$ = 7.4 Hz, $^2J_{PtC}$ = 64.6 Hz, N*C*H), 84.1 (t, $^1J_{PC}$ = 31.4 Hz, $^2J_{PtC}$ = 30.8 Hz, *C*HP), 68.7 (s, $^2J_{PtC}$ = 58.7 Hz, O*C*H$_2$CH$_3$), 49.6 (t, $^2J_{PC}$ = 9.6 Hz, $^2J_{PtC}$ = 893.8 Hz, Pt*C*CO), 36.6 (vt, $^1J_{PC}$ = 13.0 Hz, $^2J_{PtC}$ = 38.9 Hz, P*C*Me$_3$), 29.3 (vt, $^1J_{PC}$ = 3.0 Hz, $^3J_{PtC}$ = 14.3 Hz, PC*Me*$_3$). ^{31}P{^{1}H} NMR (121 MHz, C_6D_6, 298 K): δ (ppm) 62.4 (s, $^1J_{PtP}$ = 2682.1 Hz). IR (ATR): 2023 (C=C=O), 1530 (C=C). IR

(in THF): 2029 (540, C=C=O), 1532 (1941, C=C). UV/Vis (THF): λ_{max} [nm] (ε [$M^{-1}cm^{-1}$]) = 320 (20300). LIFDI-MS (m/z): $[M]^+$ calcd. for $[C_{24}H_{45}NO_2P_2Pt]^+$: 636.3; found, 636.3. Anal. Calcd for $C_{24}H_{45}NO_2P_2Pt$: C, 45.28; H, 7.12; N, 2.20. Found: C, 45.42; H, 7.15; N, 2.59.

Synthesis of 2 from 3

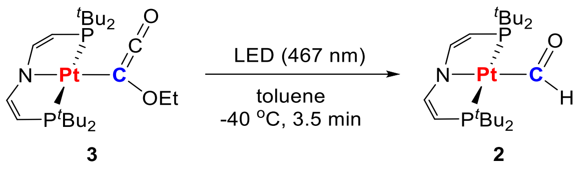

Complex **3** (2.9 mg, 0.0045 mmol) was placed in a J. Young NMR tube and dissolved in toluene-d_8 (*ca.* 0.4 ml). The solution was photolyzed with a 467 nm LED at –40 °C for 1 hour. ^{1}H NMR spectra indicated the full conversion of complex **3** along with the formation of complex **2** in 83% NMR yield, as well as ethylene.

1.4 Spectroscopic Characterization

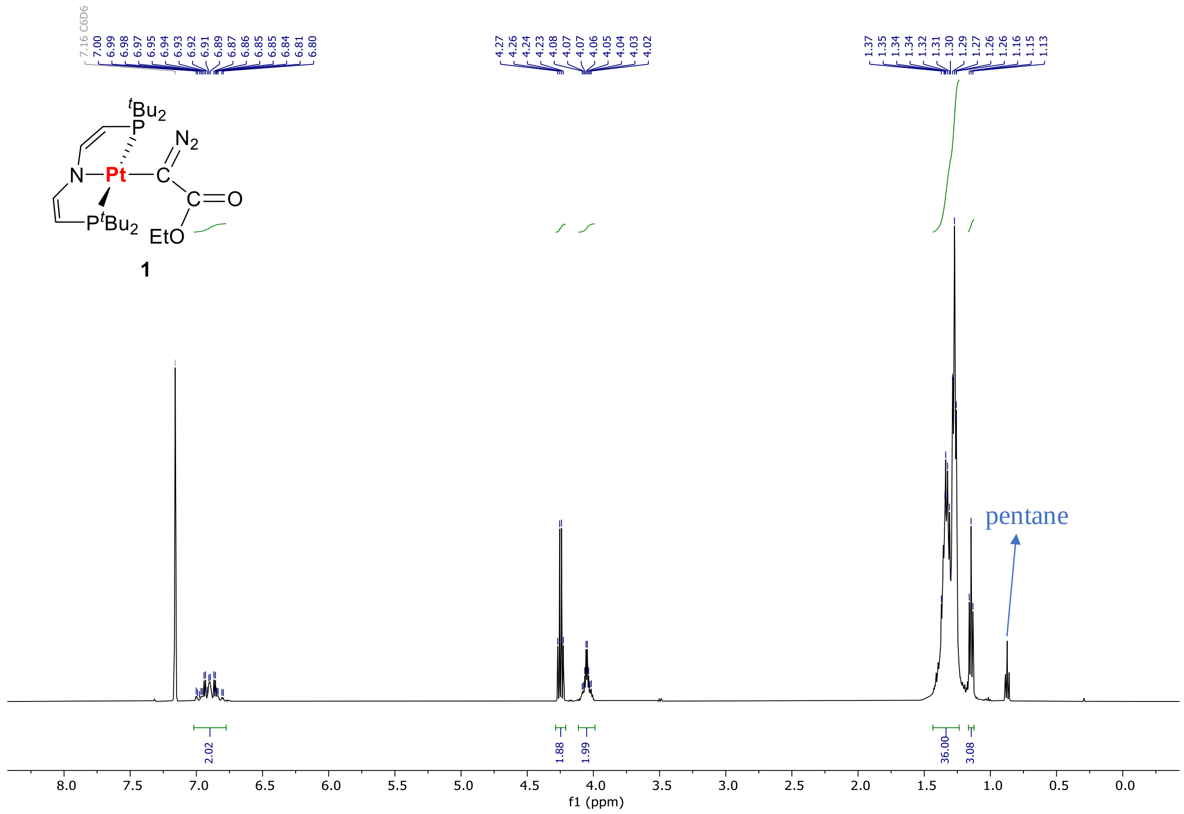

Figure S1. ^{1}H NMR spectrum of [Pt{C(N_2)CO_2Et}(PNP)] (**1**) in C_6D_6.

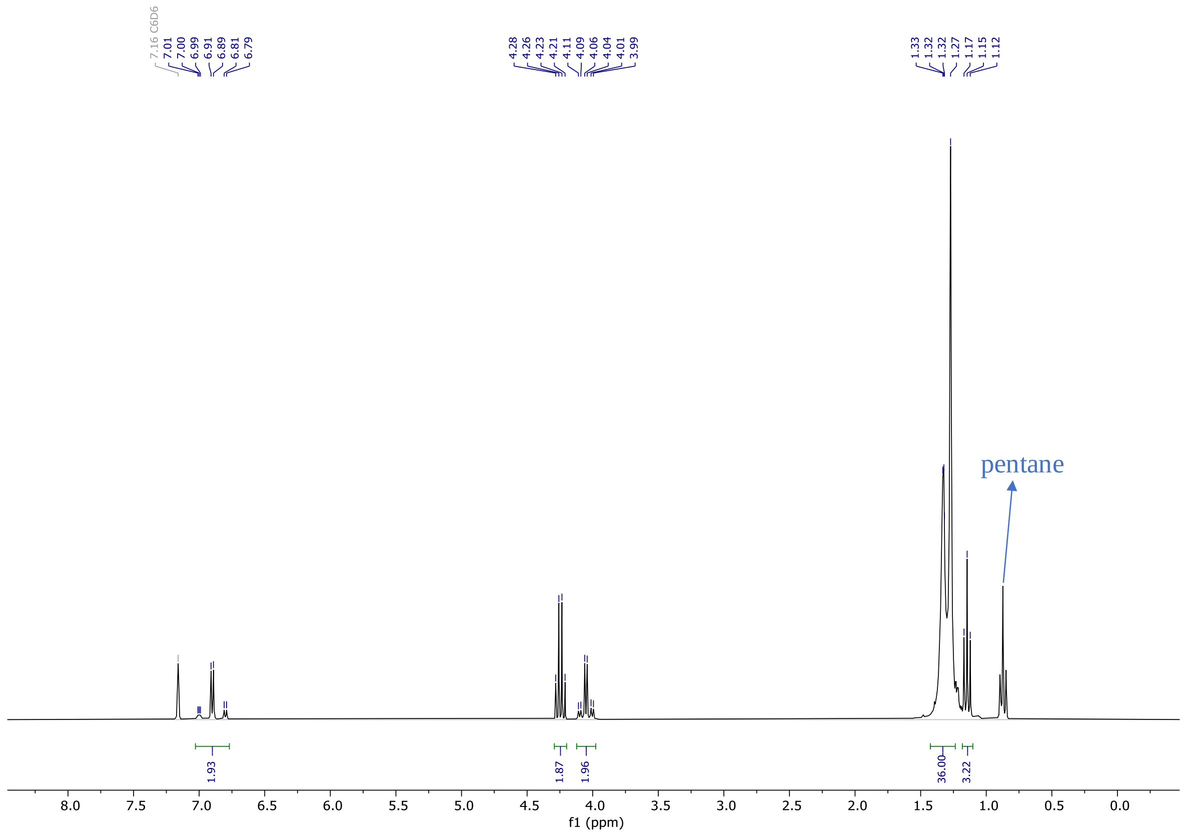

Figure S2. ^{1}H{^{31}P} NMR spectrum of [Pt{C(N_2)CO_2Et}(PNP)] (**1**) in C_6D_6.

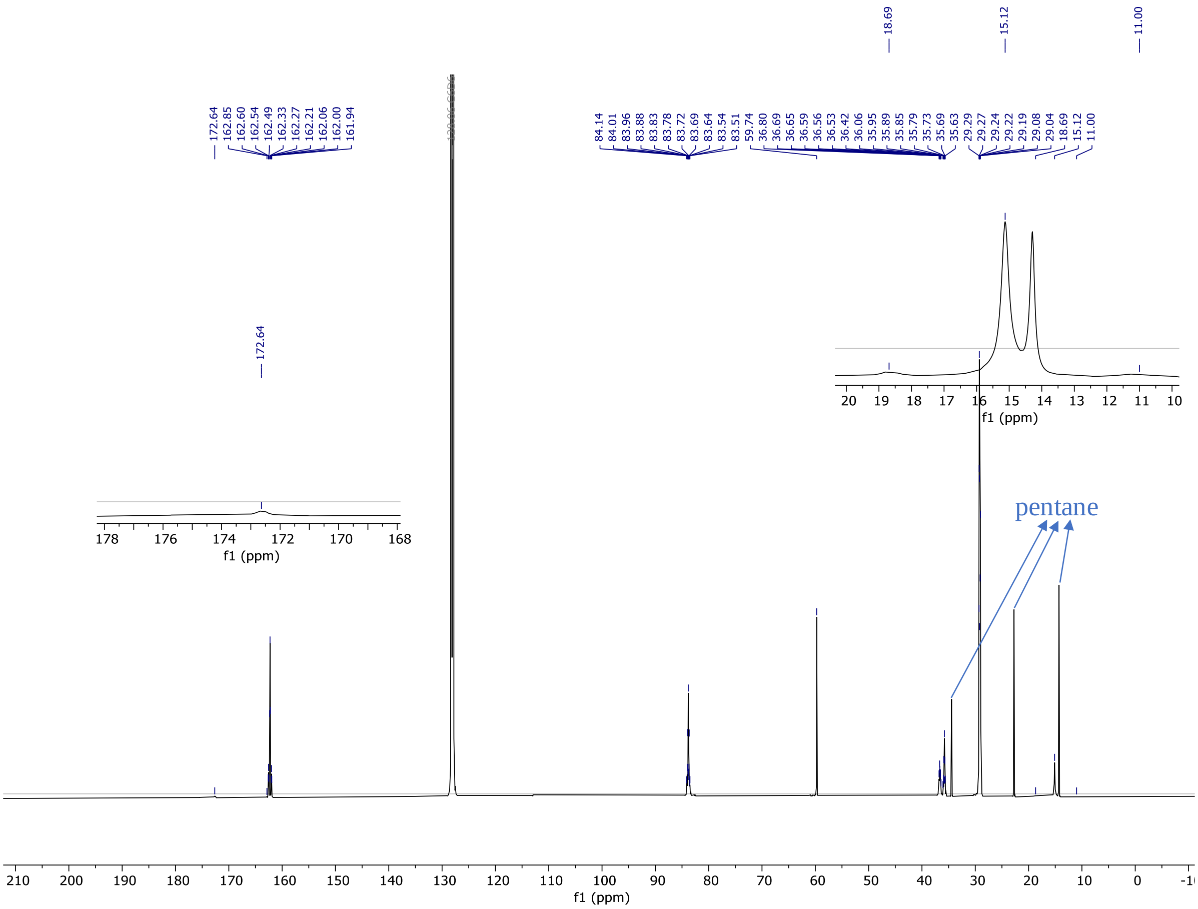

Figure S3. $^{13}C\{^1H\}$ NMR spectrum of $[Pt\{C(N_2)CO_2Et\}(PNP)]$ (**1**) in C_6D_6.

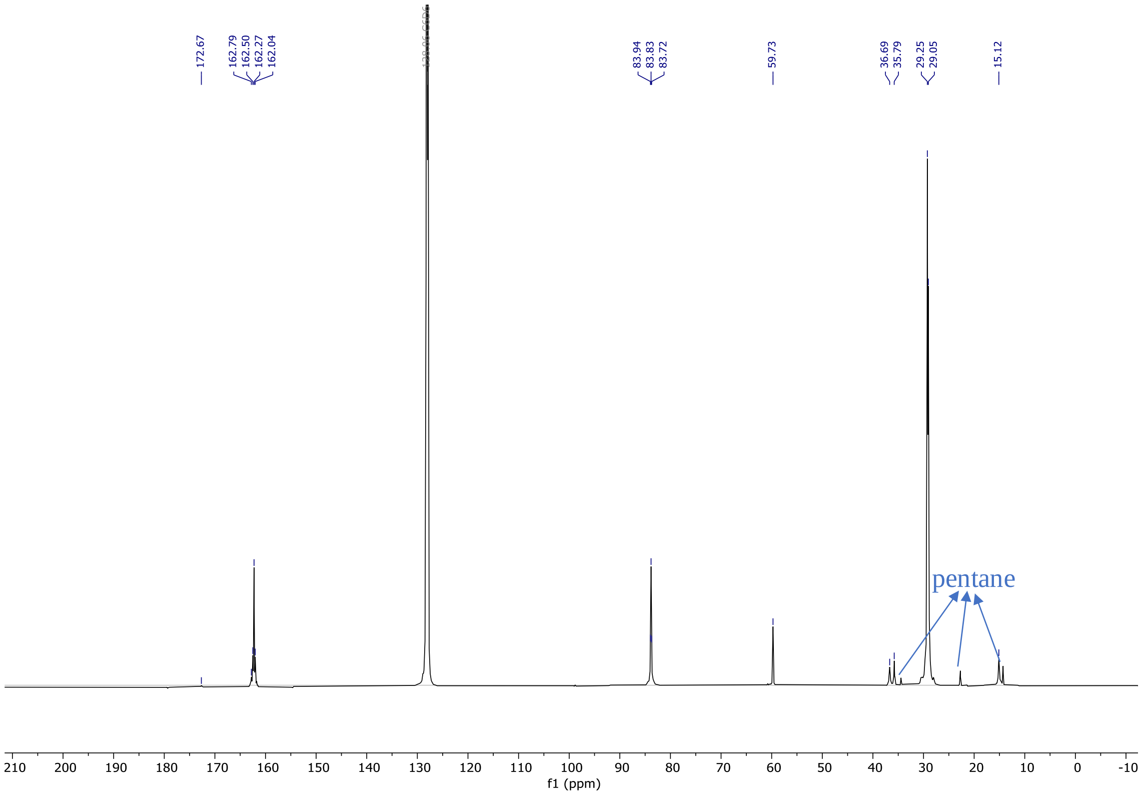

Figure S4. $^{13}C\{^1H,^{31}P\}$ NMR spectrum of $[Pt\{C(N_2)CO_2Et\}(PNP)]$ (**1**) in C_6D_6.

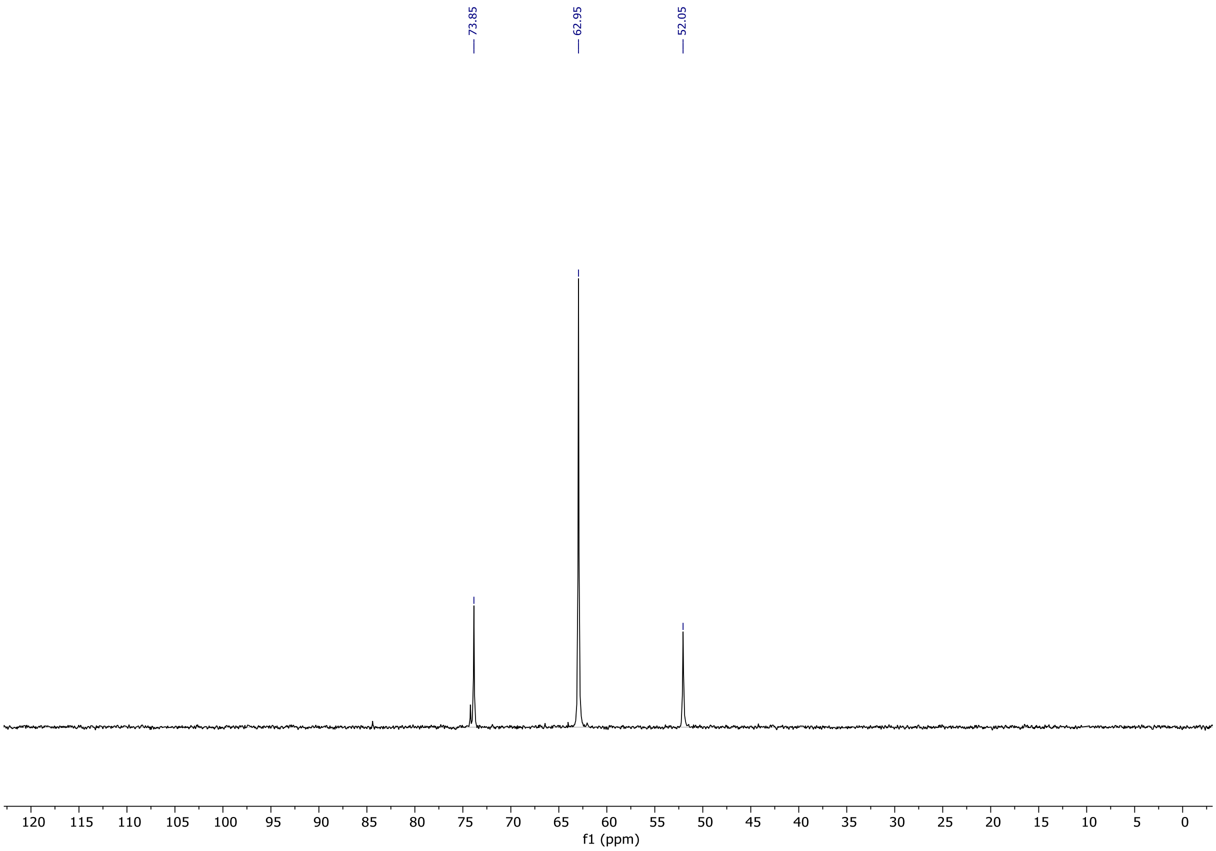

Figure S5. $^{31}P\{^{1}H\}$ NMR spectrum of [Pt{C(N_2)CO_2Et}(PNP)] (**1**) in C_6D_6.

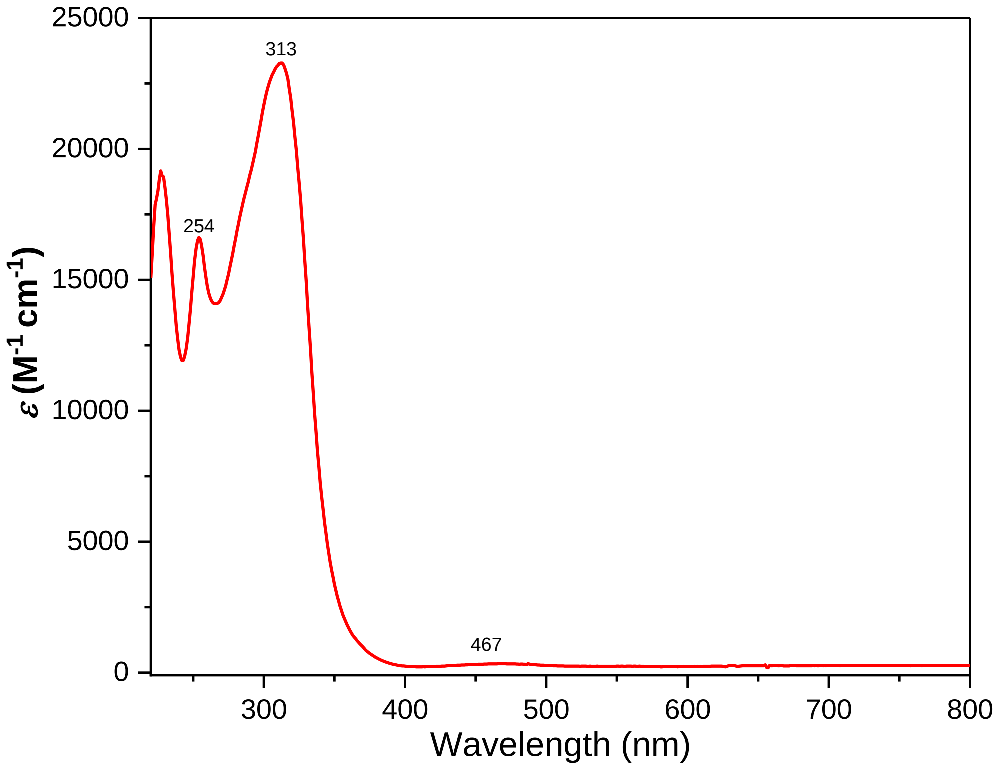

Figure S6. UV/Vis spectrum of [Pt{C(N_2)CO_2Et}(PNP)] (**1**) in THF.

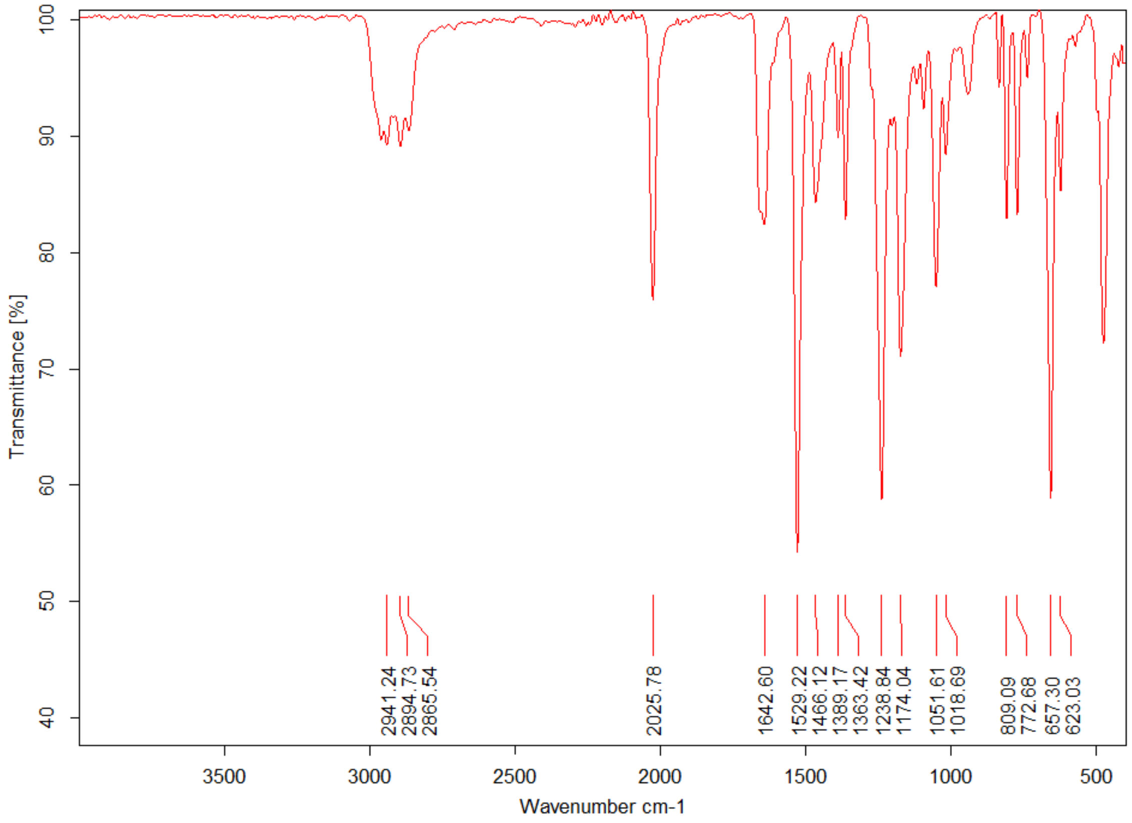

Figure S7. ATR-IR spectrum of [Pt{C(N_2)CO_2Et}(PNP)] (**1**).

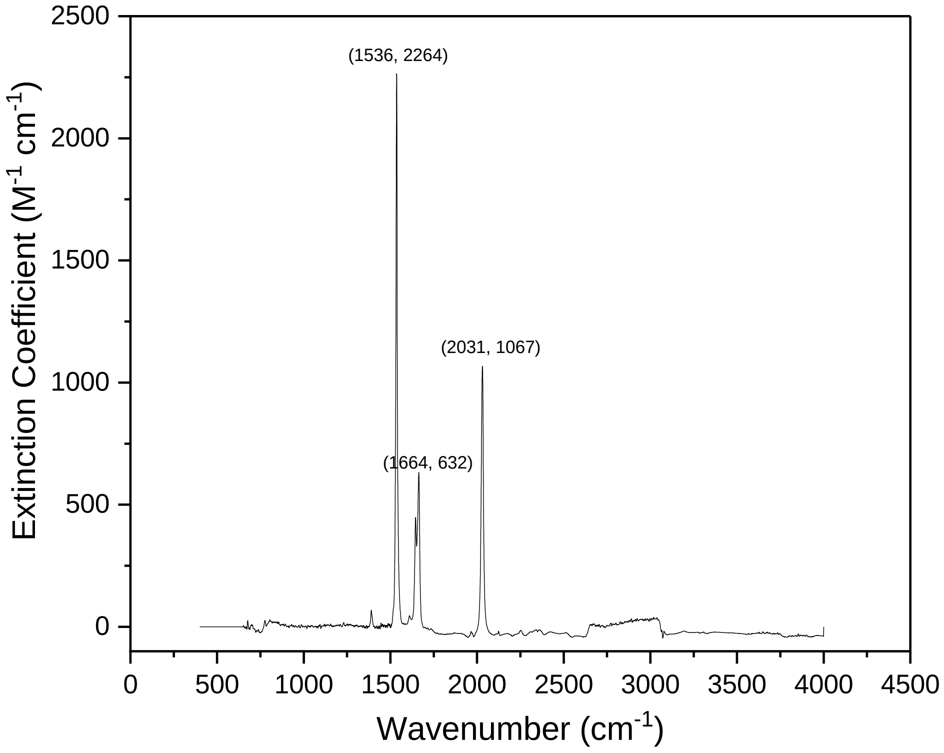

Figure S8. IR spectrum of [Pt{C(N_2)CO_2Et}(PNP)] (**1**) in THF.

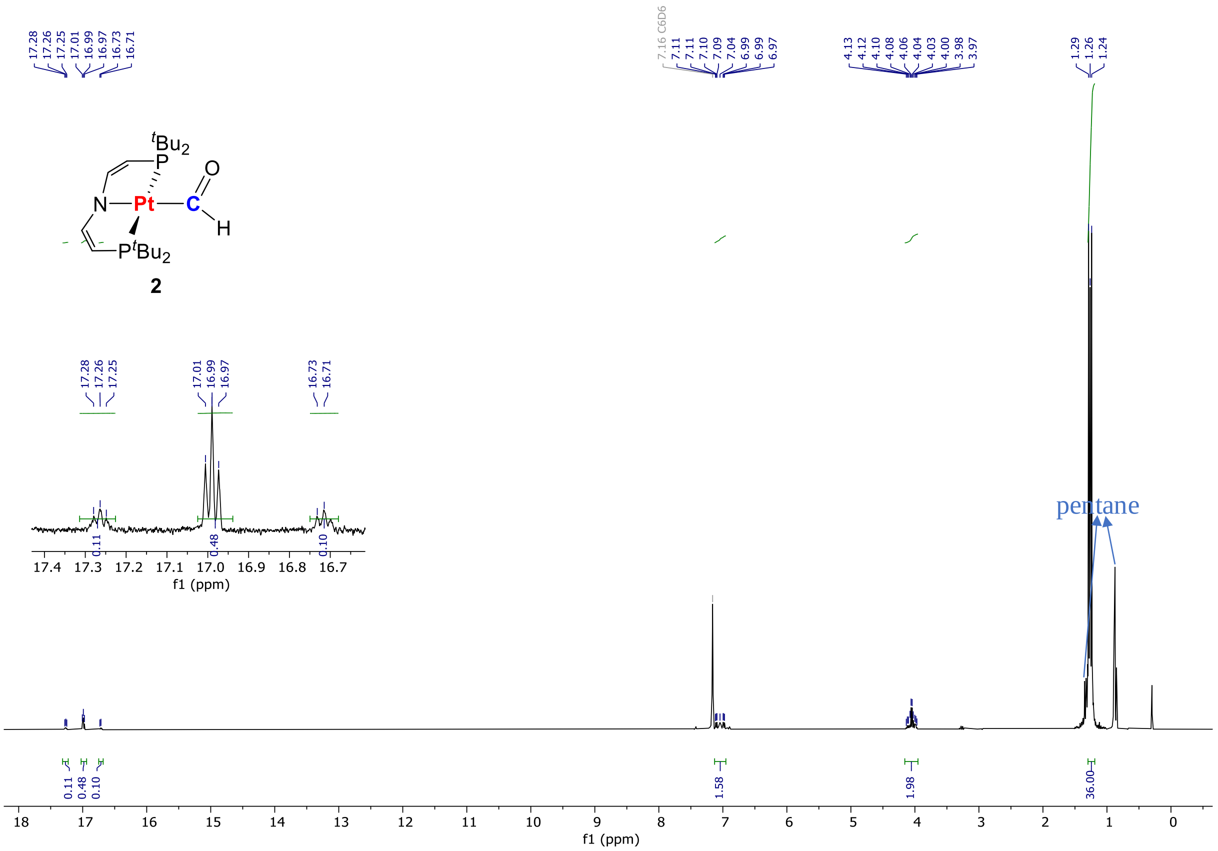

Figure S9. ^{1}H NMR spectrum of [Pt(CHO)(PNP)] (**2**) in C_6D_6.

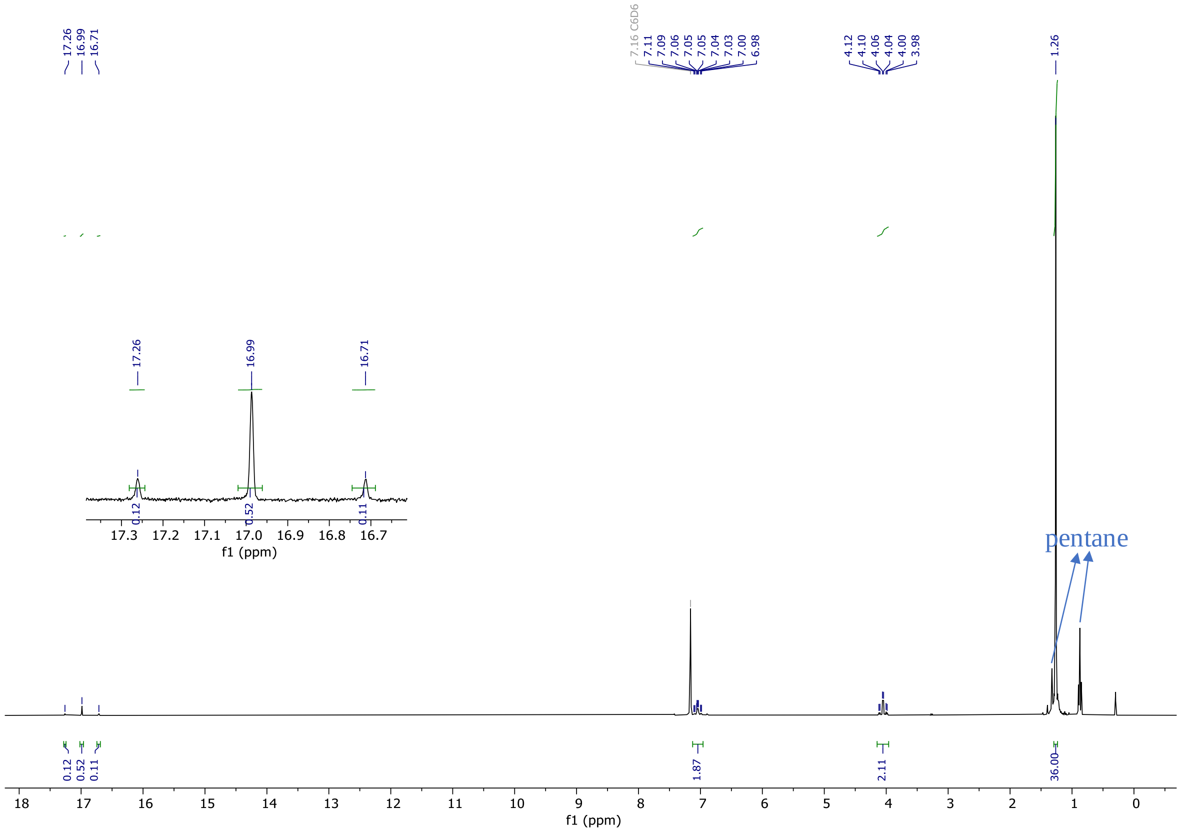

Figure S10. ^{1}H{^{31}P} NMR spectrum of [Pt(CHO)(PNP)] (**2**) in C_6D_6.

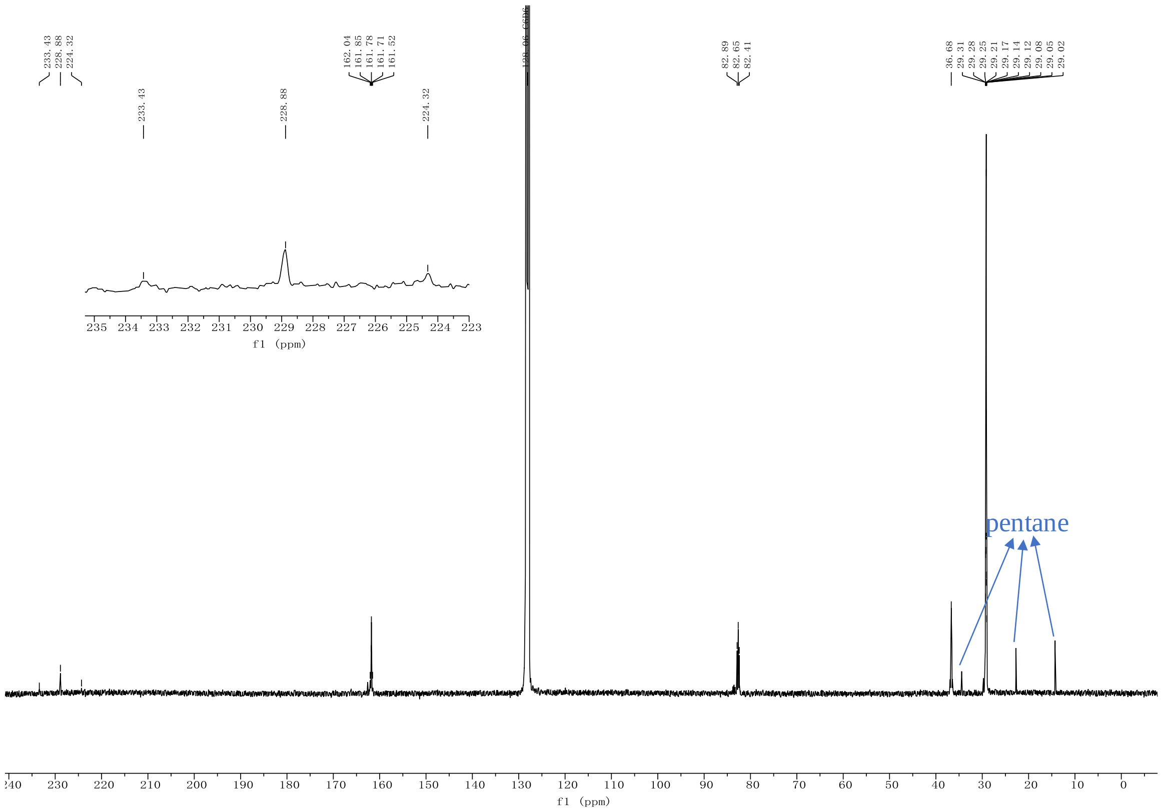

Figure S11. $^{13}C\{^{1}H\}$ NMR spectrum of [Pt(CHO)(PNP)] (**2**) in C_6D_6.

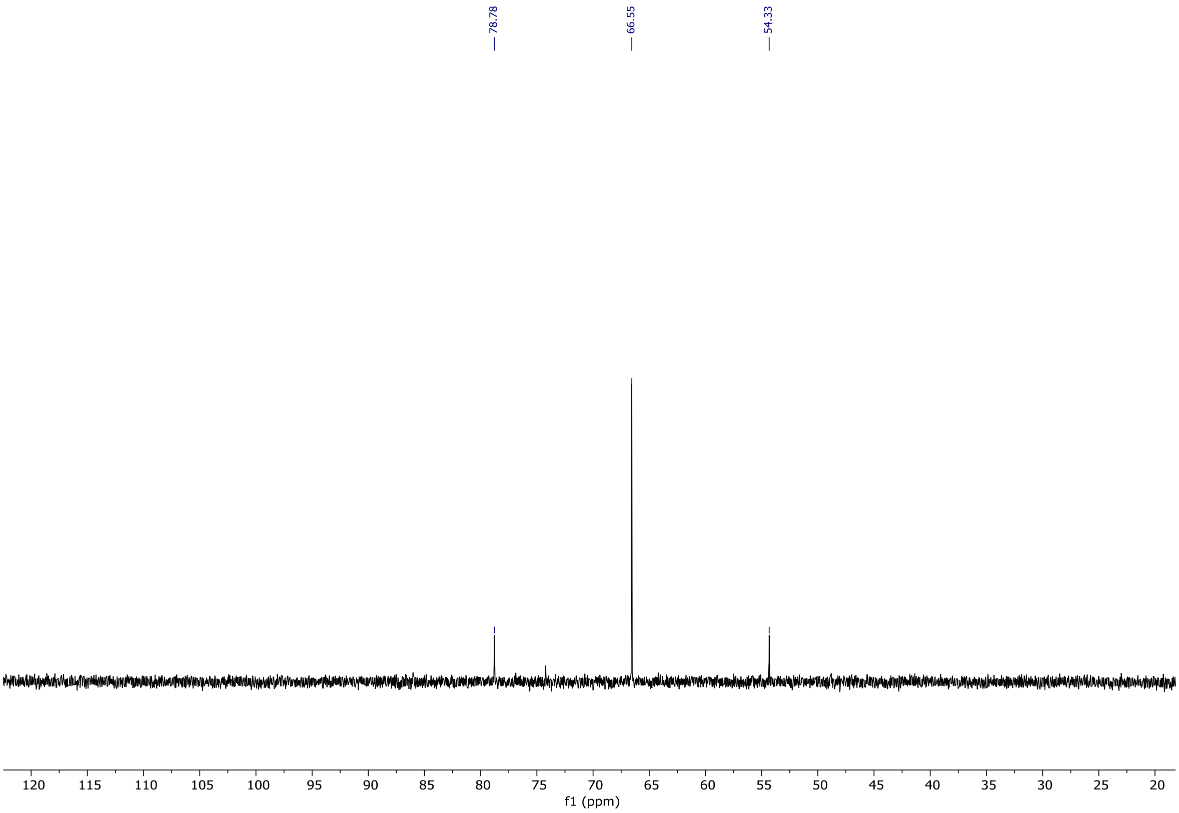

Figure S12. $^{31}P\{^{1}H\}$ NMR spectrum of [Pt(CHO)(PNP)] (**2**) in C_6D_6.

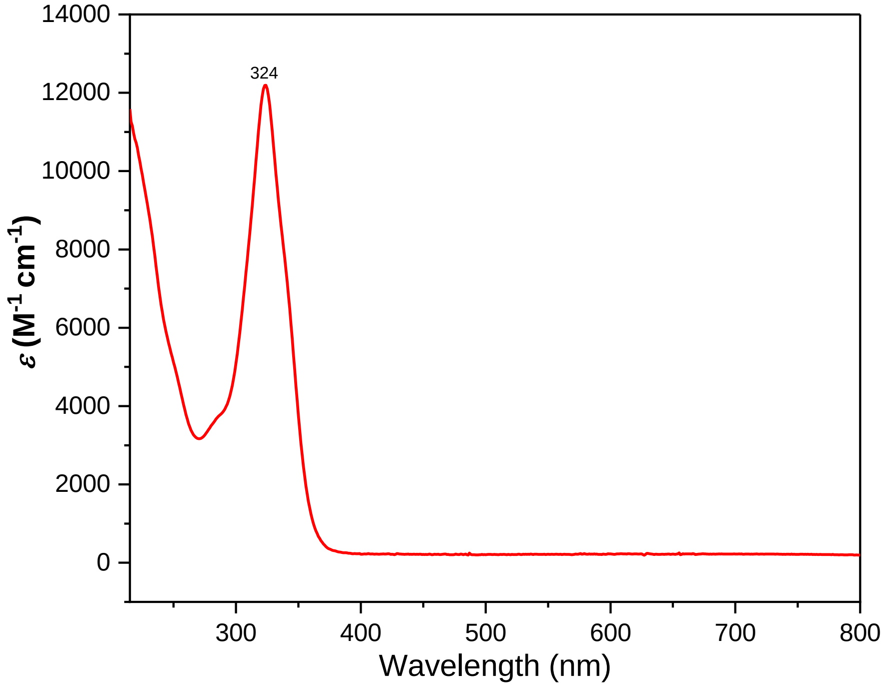

Figure S13. UV/Vis spectrum of [Pt(CHO)(PNP)] (**2**) in THF.

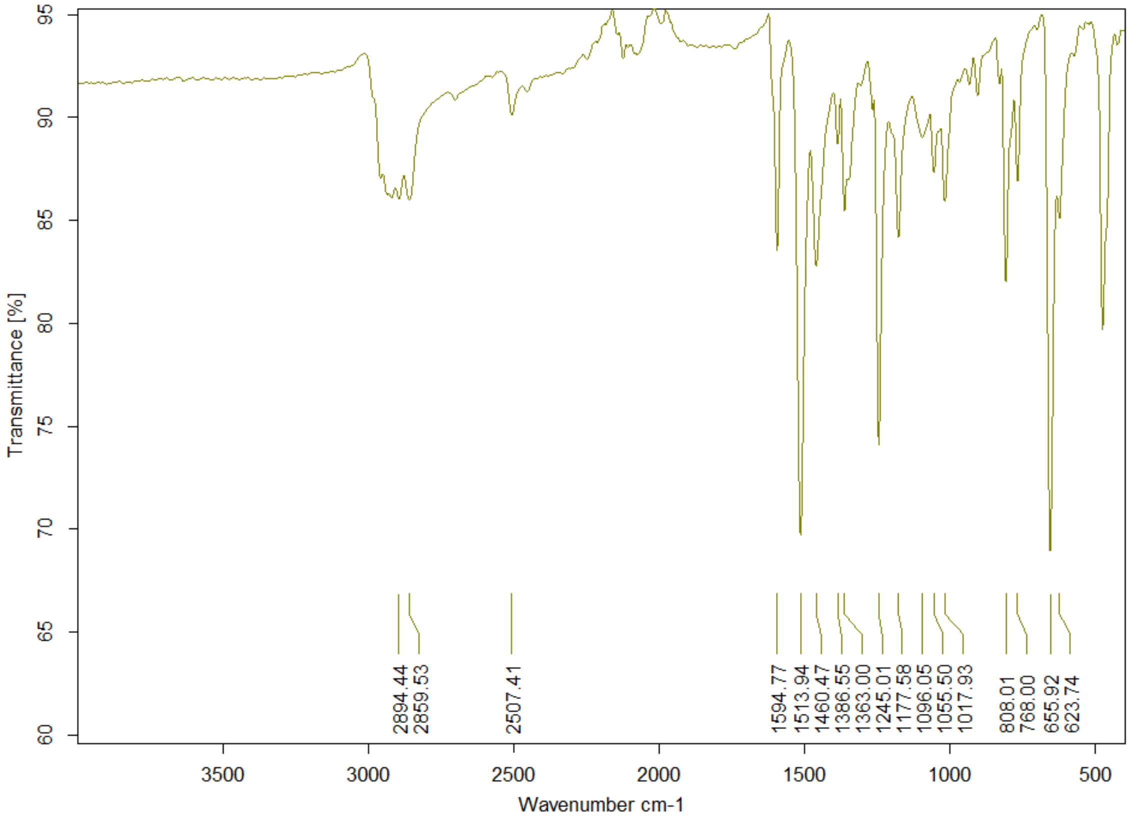

Figure S14. ATR-IR spectrum of [Pt(CHO)(PNP)] (**2**).

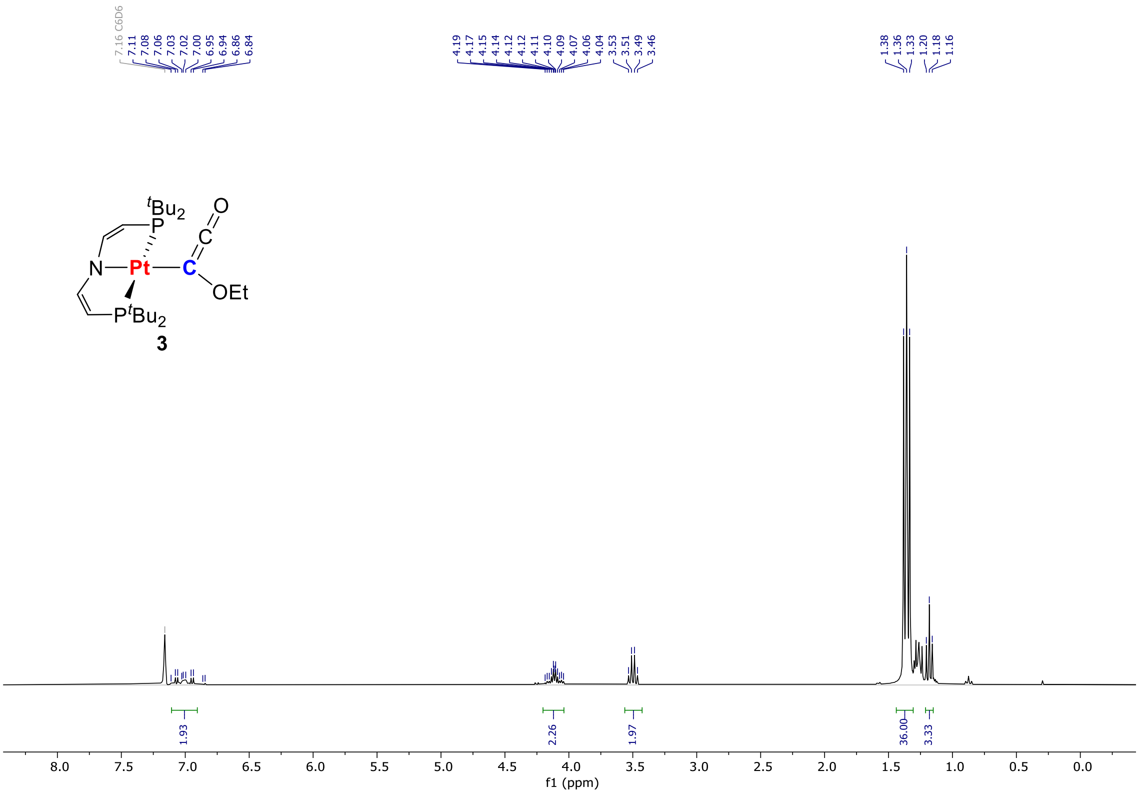

Figure S15. ^{1}H NMR spectrum of [Pd{C(CO)OEt}(PNP)] (**3**) in C_6D_6.

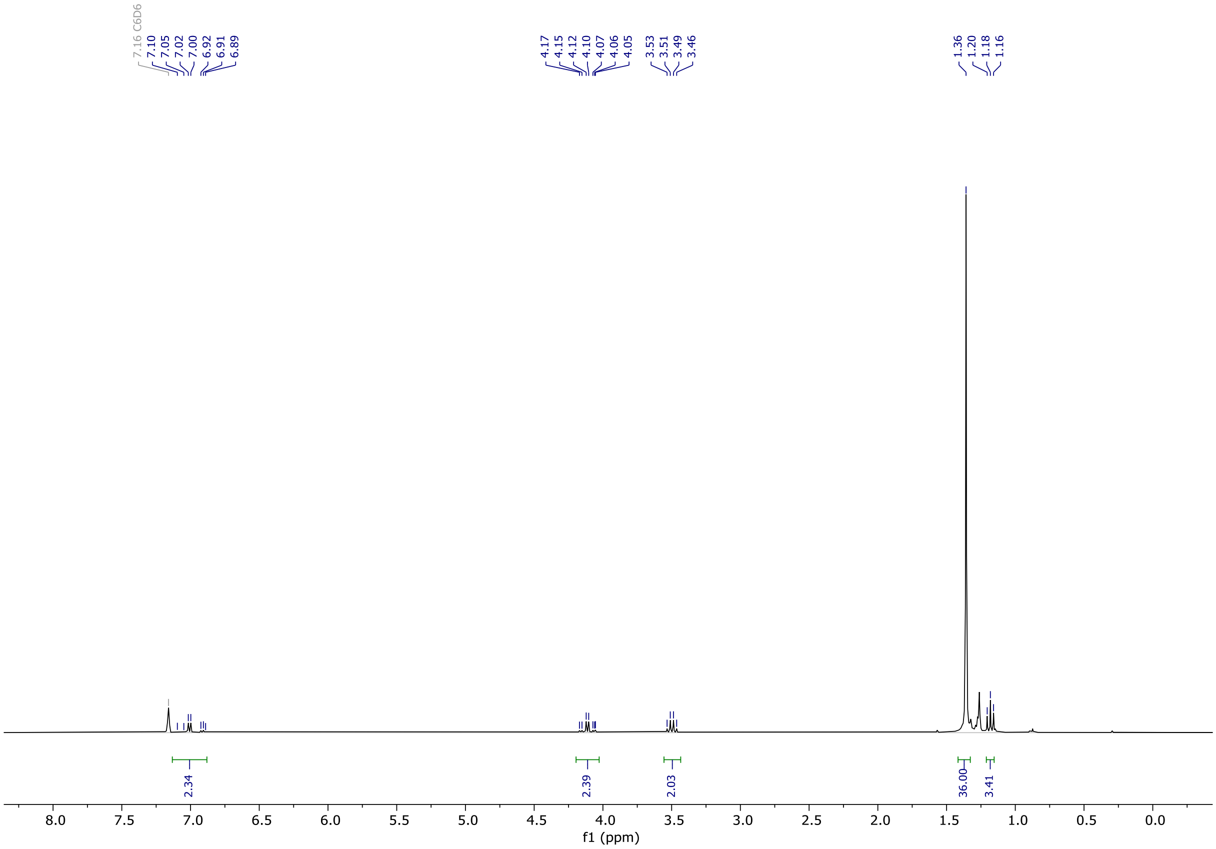

Figure S16. ^{1}H{^{31}P} NMR spectrum of [Pd{C(CO)OEt}(PNP)] (**3**) in C_6D_6.

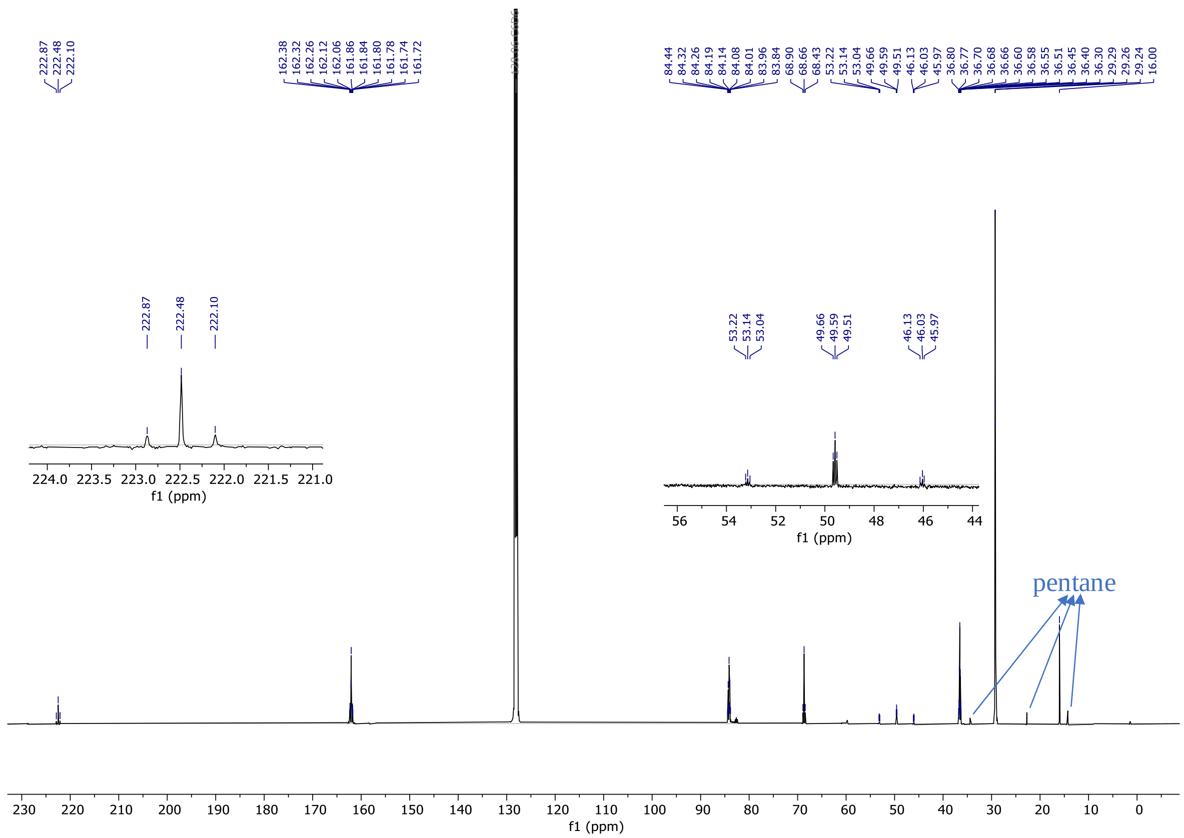

Figure S17. $^{13}C\{^{1}H\}$ NMR spectrum of [Pd{C(CO)OEt}(PNP)] (**3**) in C_6D_6.

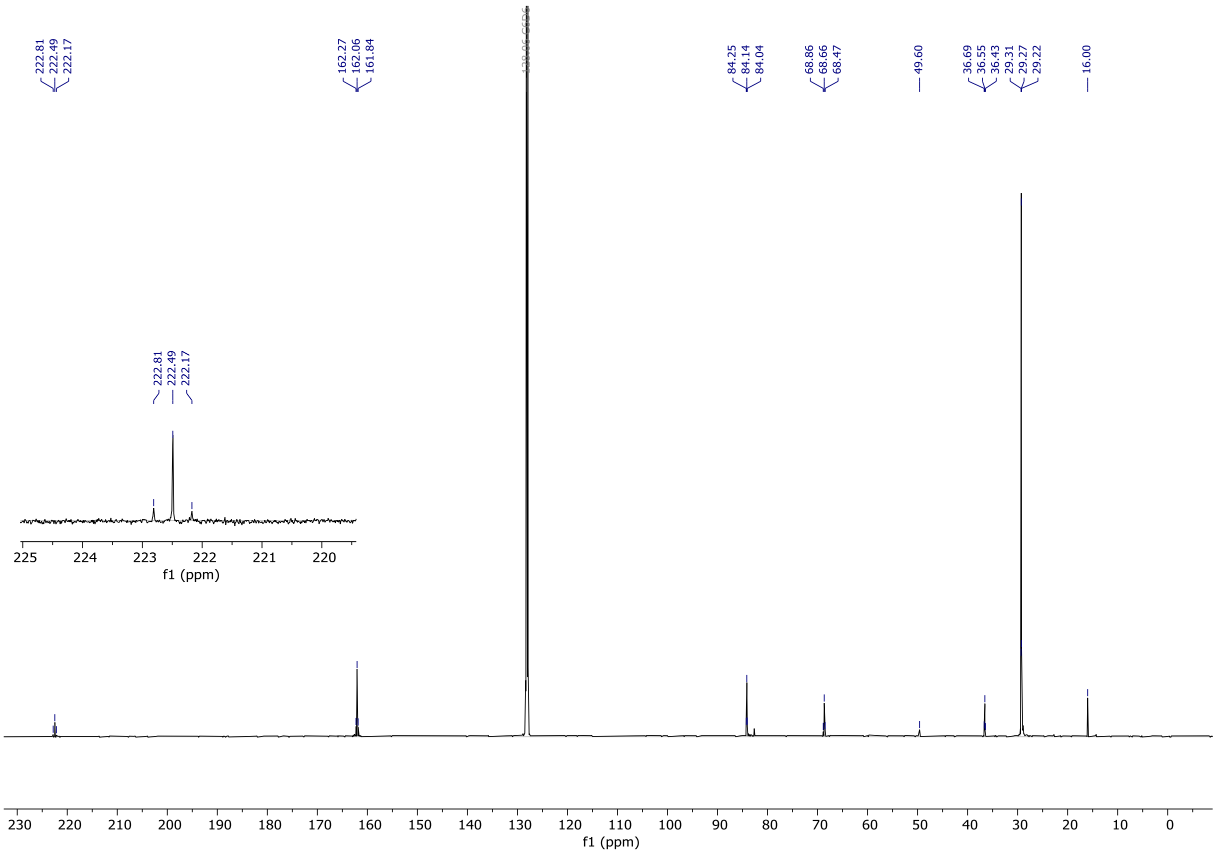

Figure S18. $^{13}C\{^{1}H, ^{31}P\}$ NMR spectrum of [Pd{C(CO)OEt}(PNP)] (**3**) in C_6D_6.

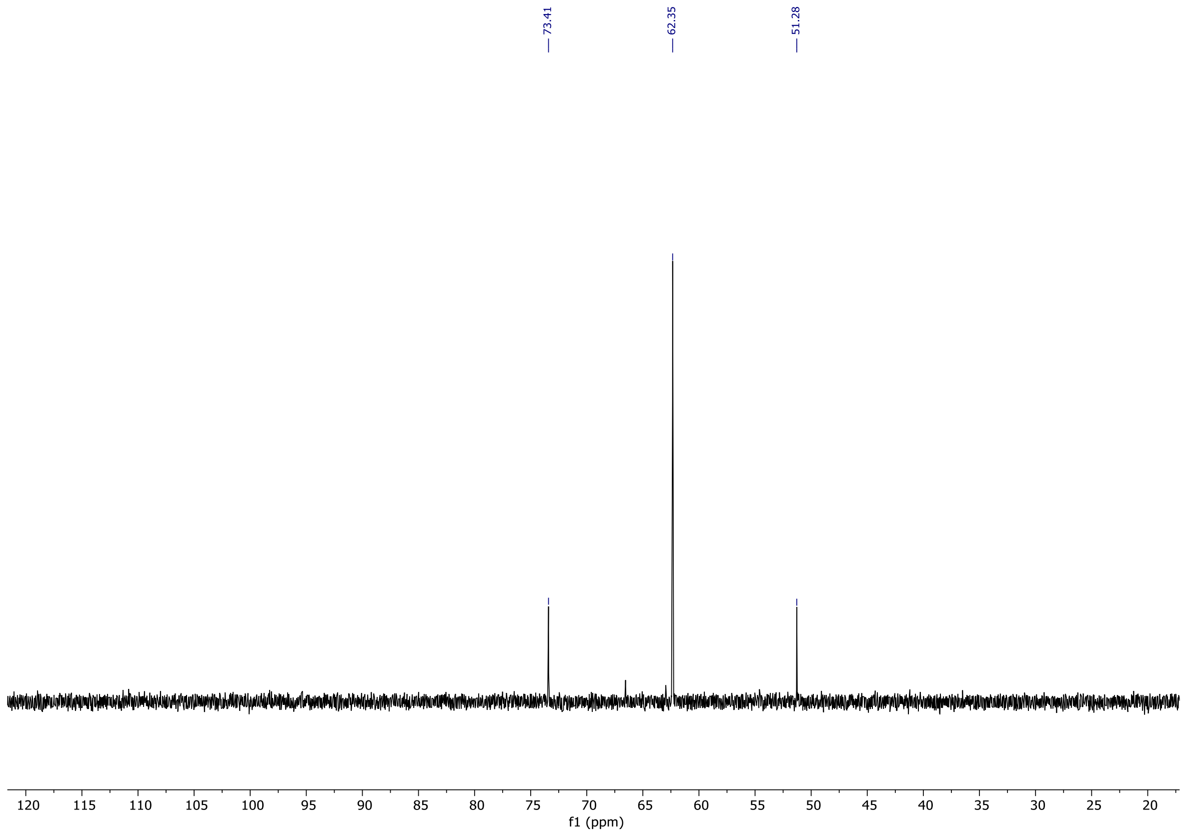

Figure S19. $^{31}P\{^1H\}$ NMR spectrum of [Pd{C(CO)OEt}(PNP)] (**3**) in C_6D_6.

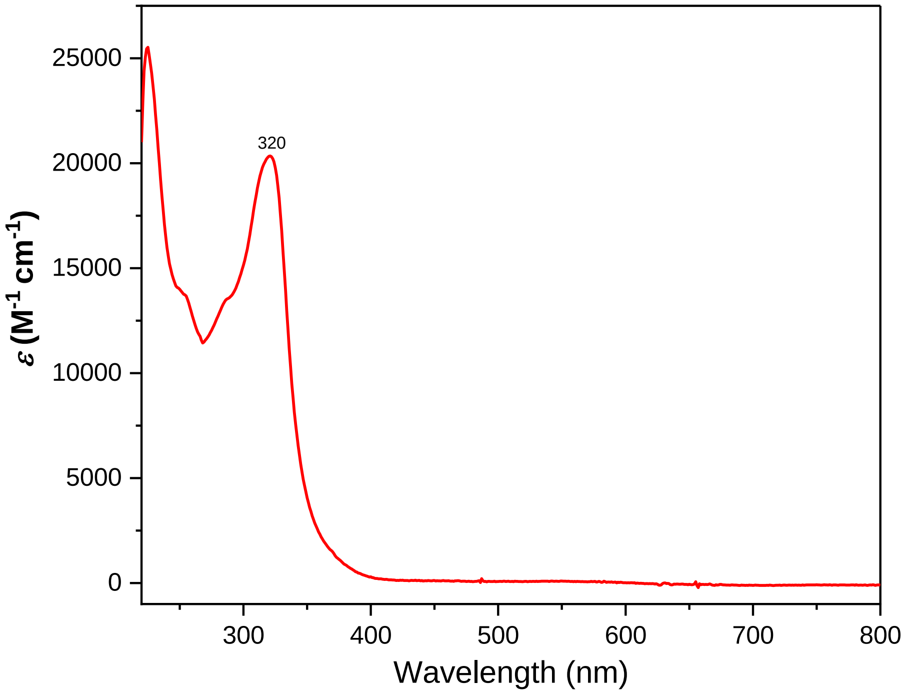

Figure S20. UV/Vis spectrum of [Pd{C(CO)OEt}(PNP)] (**3**) in THF.

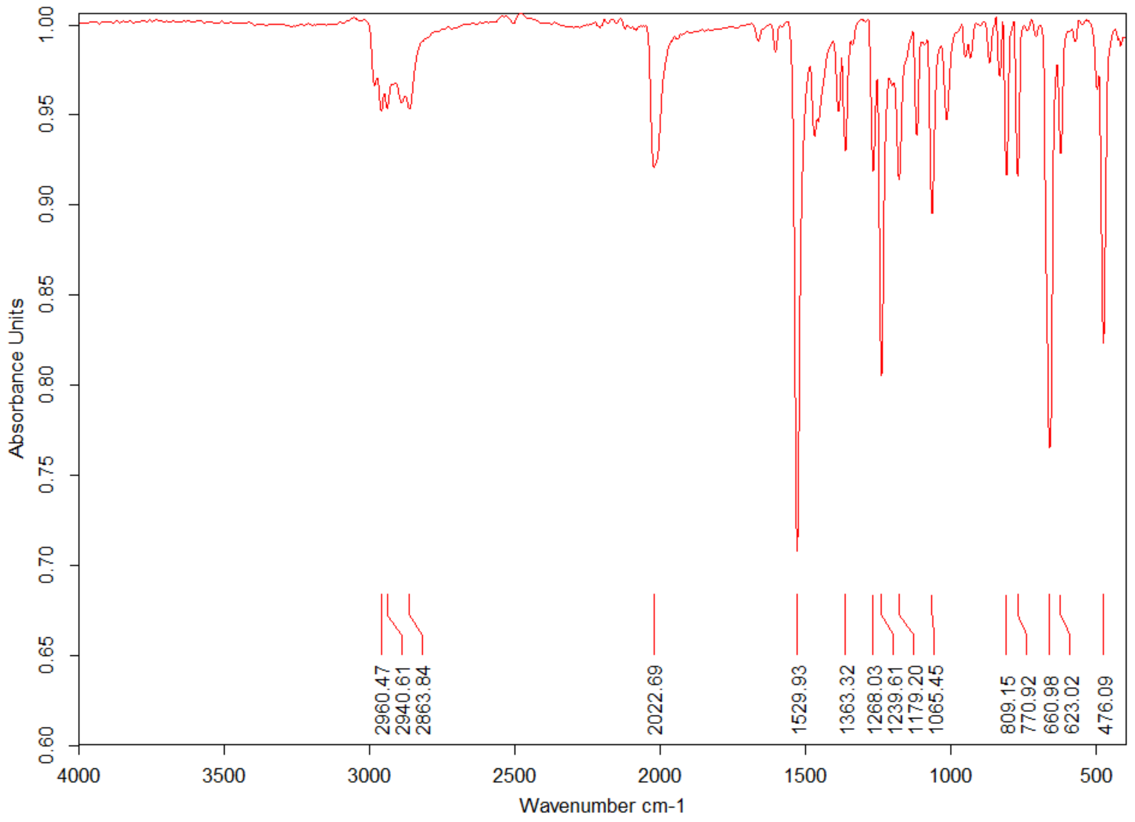

Figure S21. ATR-IR spectrum of [Pd{C(CO)OEt}(PNP)] (**3**).

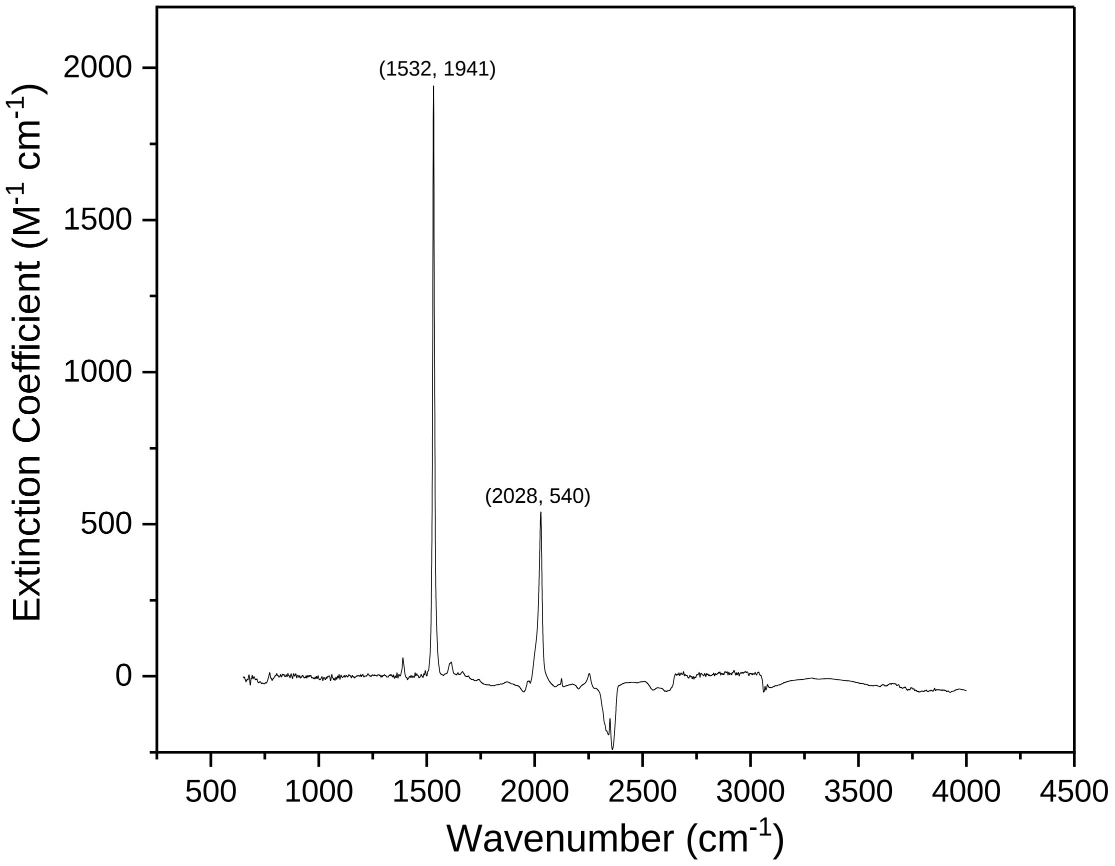

Figure S22. IR spectrum of [Pd{C(CO)OEt}(PNP)] (**3**) in THF.

1.5 Determination of Quantum Yields

The photon flux of the 450 nm LED ($I = 3.4 \cdot 10^{-8}$ mol s^{-1}) was determined actinometry, utilizing the photoisomerization of 2,4-dinitrobenzaldehyde, which has a quantum yield of ϕ = 0.08 at λ = 440 nm.[2]

Quantum yield for the conversion of 1 to 3

1 (1.3 mg, $1.9 \cdot 10^{-3}$ mmol) was dissolved in THF-d_8 (0. 35 mL) and the mixture was photolyzed in the NMR spectrometer for t = 120 s at room temperature. NMR spectra indicated the formation of **3** in 51% NMR yield ($\Delta n = 9.9 \cdot 10^{-4}$ mmol). The absorbance (A_{450}) is determined by UV-vis spectroscopy (A_{450} = 0.70). The quantum yield was calculated from the following equation:

$$\phi_{450} = \frac{\Delta n}{I \cdot t \cdot (1 - 10^{-A_{450}})} = (31 \pm 5)\%$$

The experiment was performed twice for estimation of the standard deviation.

Quantum yield for the conversion of 3 to 2

3 (1.3 mg, $2.0 \cdot 10^{-3}$ mmol) was dissolved in THF-d_8 (0. 30 mL) and the mixture was photolyzed in the NMR spectrometer for t = 3000 s at room temperature. NMR spectra indicated the formation of **2** in 54% NMR yield (Δn = $1.1 \cdot 10^{-3}$ mmol). The absorbance (A_{450}) is determined by UV-vis spectroscopy (A_{450} = 0.050). The quantum yield was calculated from the following equation:

$$\phi_{450} = \frac{\Delta n}{I \cdot t \cdot (1 - 10^{-A_{450}})} = (9 \pm 2)\%$$

The experiment was performed twice for estimation of the standard deviation.

2. Crystallographic Details

2.1 General Details

Suitable single crystals for X-ray structure determination were selected from the mother liquor under an inert gas atmosphere and transferred in protective perfluoro polyether oil on a microscope slide. The selected and mounted crystals were transferred to the cold gas stream on the diffractometer. The diffraction data were obtained at 80, 100 or 120 K on a Bruker D8 three-circle diffractometer, equipped with a PHOTON-III detector and an INCOATEC microfocus source with Quazar mirror optics (Mo-$K\alpha$ radiation, λ = 0.71073 Å). The data obtained were integrated with SAINT[3a] and a semi-empirical absorption correction from equivalents with SADABS[3b] was applied. The structures were solved with SHELXT[3c] using direct methods and refined with the SHELXL[3d] within the graphical interfaces Olex2 and Shelxle.[5,6] Refinement was performed on F^2, anisotropically for all the non-hydrogen atoms by the full-matrix least-squares method. The hydrogen atoms were placed at calculated positions and were refined with the riding model.

Crystallographic data have been deposited with the Cambridge Crystallographic Data Centre with supplementary publication numbers: CCDC-2409642 ([Pt{C(N_2)CO_2Et}(PNP)] (**1**)), CCDC-2409643 ([Pt(CHO)(PNP)] (**2**)), CCDC-2409644 ([Pd{C(CO)OEt}(PNP)] (**3**)), CCDC-2409646 ([Pt(CCO_2Et)(PNP)] (**4**)), CCDC-2409645 (the crystal of **3** obtained from crystal-to-crystal transformation) contain the supplementary crystallographic data for this paper. These data can be obtained free of charge from https://www.ccdc.cam.ac.uk/structures/ (or from Cambridge Crystallographic Data Centre, 12 Union Road, Cambridge, CB2 1EZ, UK. Fax: +44-1223-336-033; e-mail: deposit@ccdc.cam.ac.uk).

2.2 Crystal-to-crystal Transformation Experiments

Photocrystallographic Characterization of 4

A crystal of **1** was mounted on a goniometer and photolyzed with a 530 nm LED lamp for 30 min under a steady flow of dinitrogen at 80 K (see Figure S23a for the setup). The crystal was rotated every 5 minutes by 90° to allow for equal irradiation from all sites. *In crystallo* reaction progress was monitored by periodic collection and analysis of X-ray diffraction data, which confirmed that the occupancy of the diazo nitrogen atoms decreased over time. Over the course of the experiment, the crystal turned from orange (Figure S23b) to green (Figure S23c) with preserved crystallinity. Longer photolysis led to insufficient data quality. Upon irradiation, the cell volume expanded by 4.3%, accompanied by minor alterations of the cell parameters (below 4.1% in all cases). Refinement of the data set revealed the photoinduced formation of **4** accompanied by dinitrogen loss in 64% yield, as derived by refined partial occupation of the C21/N2–N3 and C21a–N2a–N3a moieties, respectively.

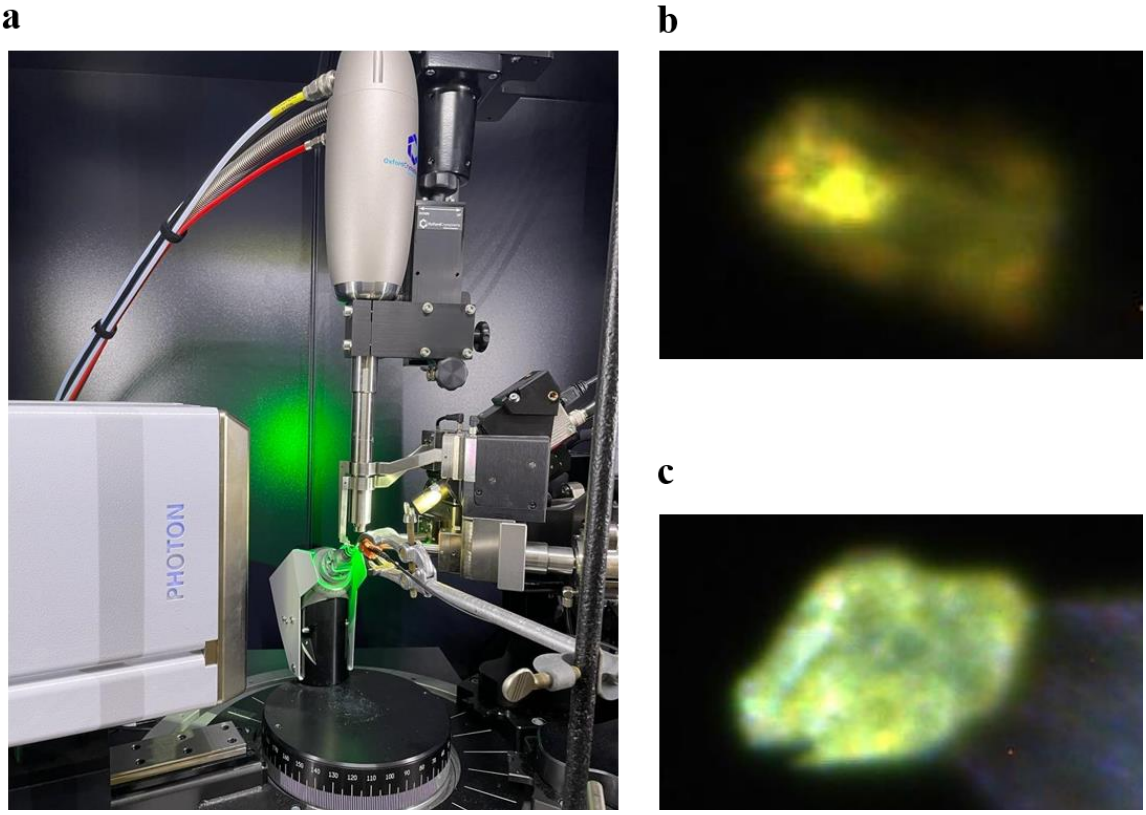

Figure S23. Experimental setup for the photocrystallographic characterization of **4**. **a**, Photolysis of a crystal of **1** with a 530 nm LED light source. **b**, Orange crystal of complex **1** before photolysis. **c**, Green crystal after photolysis.

Crystal-to-crystal conversion from 4 to 3.

A crystal of **1** was photolyzed at 80 K for 40 min on a goniometer with a 530 nm LED lamp as described in the previous section. Collection of the X-ray diffraction data and analysis confirmed the conversion to **4** in 72% yield. After that, the temperature of the dinitrogen cooling was raised to 120 K and the crystal kept at that temperature for 12 h. During this process, the color of the crystal gradually turned from green to light violet (Figure S24). After that, the crystallographic data was collected at 120 K. The data could be refined as a mixture of **1** (28%) and **3** (72%), as derived by refined partial occupation of the C21–C22–O1 and C21a–N2a–N3a moieties, respectively. The free dinitrogen molecule formed in the photolytic step escaped from the crystal at 120 K.

a b

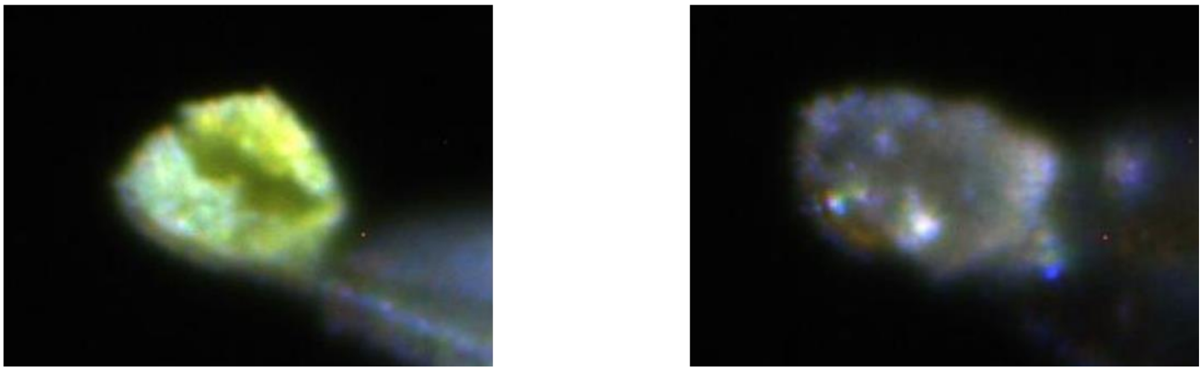

Figure S24. Crystal-to-crystal conversion from **4** to **3**. **a**, Green crystal of complex **4** from photolysis of **1** at 80 K. **b**, Slight violet crystal after keeping the crystal of **4** at 120 K for 12 h.

2.3 Crystal Structure of [Pt{C(N_2)CO_2Et}(PNP)] (1)

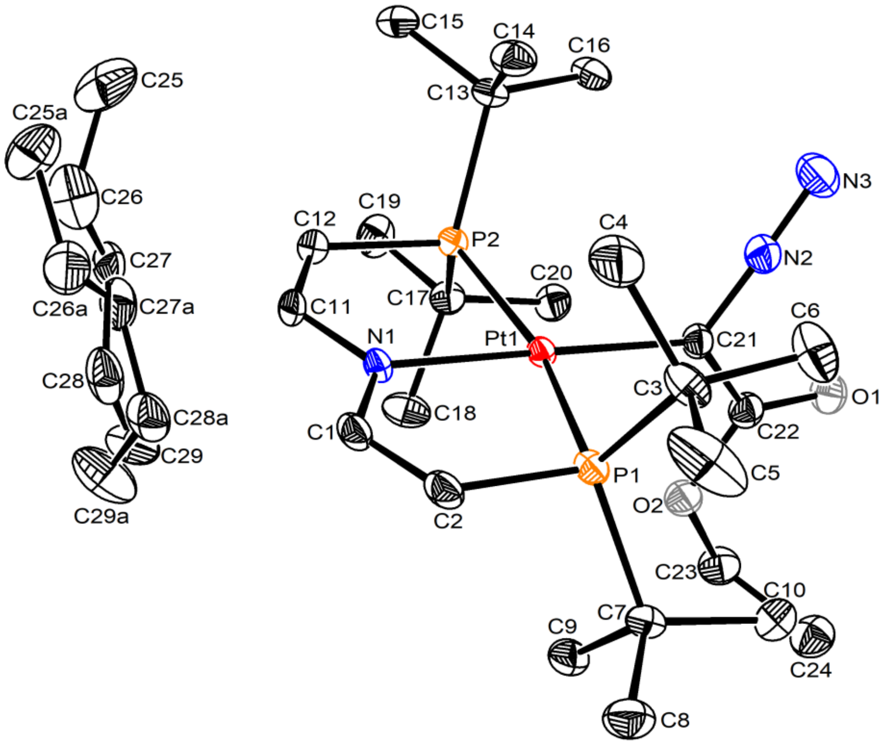

Figure S25. Asymmetric unit of [Pt{C(N_2)CO_2Et}(PNP)] (**1**) with 50% thermal ellipsoids, CCDC deposition number: 2409642. H atoms are omitted for clarity. The asymmetric unit contains one complex molecule and half of pentane solvent molecule. The pentane molecule was disordered over two postions about an inversion centre. It was refined with distance restraints and restraint for the anisotropic parameters. The occupancy of the minor component refined to 0.133(10).

Table S1. Crystal data and structure refinement for [Pt{C(N_2)CO_2Et}(PNP)] (1).

Identification code	1
Empirical formula	$C_{26.5}H_{51}N_3O_2P_2Pt$
Formula weight	700.73
Temperature/K	80(2)
Crystal system	triclinic
Space group	*P*-1
a/Å	8.668(2)
b/Å	13.302(3)
c/Å	14.811(3)
α/°	115.79(2)
β/°	97.60(2)
γ/°	95.95(2)
Volume/Å^3	1499.1(6)
Z	2
ρ_{calc}g/cm^3	1.552

μ/mm^{-1}	4.814
F(000)	710.0
Crystal size/mm	0.158 × 0.104 × 0.076
Radiation	Mo$K\alpha$ (λ = 0.71073)
2Θ range for data collection/°	5.402 to 56.750
Index ranges	$-11 \leq h \leq 11$, $-17 \leq k \leq 17$, $-19 \leq l \leq 19$
Reflections collected	59736
Independent reflections	7453 [R_{int} = 0.0684, R_{sigma} = 0.0362]
Data/restraints/parameters	7453/ 205 / 393
Goodness-of-fit on F^2	1.050
Final R indexes [$I \geq 2\sigma (I)$]	R_1 = 0.0246, wR_2 = 0.0531
Final R indexes [all data]	R_1 = 0.0289, wR_2 = 0.0548
Largest diff. peak/hole / e Å^{-3}	1.112 / -0.760

Table S2. Bond lengths [Å] and angles [°] for [Pt{C(N_2)CO_2Et}(PNP)] (1).

Pt(1)-N(1)	2.041(3)	C(1)-C(2)	1.345(5)
Pt(1)-C(21)	2.055(3)	C(3)-C(5)	1.525(5)
Pt(1)-P(2)	2.3171(9)	C(3)-C(6)	1.530(5)
Pt(1)-P(1)	2.3187(9)	C(3)-C(4)	1.536(5)
P(1)-C(2)	1.783(3)	C(19)-C(17)	1.534(4)
P(1)-C(7)	1.866(3)	C(18)-C(17)	1.535(5)
P(1)-C(3)	1.872(3)	C(17)-C(20)	1.531(4)
P(2)-C(12)	1.786(3)	C(25)-C(26)	1.527(13)
P(2)-C(17)	1.875(3)	C(26)-C(27)	1.573(12)
P(2)-C(13)	1.877(3)	C(27)-C(28)	1.557(12)
N(1)-C(11)	1.369(4)	C(28)-C(29)	1.562(14)
N(1)-C(1)	1.370(4)	C(25A)-C(26A)	1.558(17)
O(2)-C(22)	1.355(4)	C(26A)-C(27A)	1.548(17)
O(2)-C(23)	1.456(4)	C(27A)-C(28A)	1.582(16)
O(1)-C(22)	1.218(4)	C(28A)-C(29A)	1.551(16)
N(2)-N(3)	1.142(4)		
N(2)-C(21)	1.310(4)	N(1)-Pt(1)-C(21)	178.52(12)
C(21)-C(22)	1.450(4)	N(1)-Pt(1)-P(2)	82.82(8)
C(13)-C(16)	1.529(4)	C(21)-Pt(1)-P(2)	96.64(9)
C(13)-C(14)	1.531(4)	N(1)-Pt(1)-P(1)	82.20(8)
C(13)-C(15)	1.539(5)	C(21)-Pt(1)-P(1)	98.41(9)
C(24)-C(23)	1.505(5)	P(2)-Pt(1)-P(1)	164.65(3)
C(8)-C(7)	1.532(5)	C(2)-P(1)-C(7)	104.52(15)
C(11)-C(12)	1.343(5)	C(2)-P(1)-C(3)	105.30(15)
C(9)-C(7)	1.543(5)	C(7)-P(1)-C(3)	113.42(15)
C(7)-C(10)	1.529(5)	C(2)-P(1)-Pt(1)	100.28(11)

C(7)-P(1)-Pt(1)	117.30(11)	C(10)-C(7)-C(9)	109.3(3)
C(3)-P(1)-Pt(1)	113.79(11)	C(8)-C(7)-C(9)	107.8(3)
C(12)-P(2)-C(17)	105.36(15)	C(10)-C(7)-P(1)	111.5(2)
C(12)-P(2)-C(13)	104.84(15)	C(8)-C(7)-P(1)	113.3(2)
C(17)-P(2)-C(13)	113.19(14)	C(9)-C(7)-P(1)	104.9(2)
C(12)-P(2)-Pt(1)	99.73(11)	C(2)-C(1)-N(1)	122.1(3)
C(17)-P(2)-Pt(1)	116.97(10)	O(2)-C(23)-C(24)	112.3(3)
C(13)-P(2)-Pt(1)	114.47(11)	C(5)-C(3)-C(6)	108.4(3)
C(11)-N(1)-C(1)	120.8(3)	C(5)-C(3)-C(4)	109.9(3)
C(11)-N(1)-Pt(1)	119.2(2)	C(6)-C(3)-C(4)	108.1(3)
C(1)-N(1)-Pt(1)	120.0(2)	C(5)-C(3)-P(1)	112.9(2)
C(22)-O(2)-C(23)	116.4(3)	C(6)-C(3)-P(1)	112.2(2)
N(3)-N(2)-C(21)	177.0(3)	C(4)-C(3)-P(1)	105.3(2)
N(2)-C(21)-C(22)	109.5(3)	C(1)-C(2)-P(1)	115.4(2)
N(2)-C(21)-Pt(1)	119.8(2)	C(11)-C(12)-P(2)	115.7(2)
C(22)-C(21)-Pt(1)	130.5(2)	C(20)-C(17)-C(19)	108.4(3)
C(16)-C(13)-C(14)	109.4(3)	C(20)-C(17)-C(18)	108.5(3)
C(16)-C(13)-C(15)	109.3(3)	C(19)-C(17)-C(18)	110.3(3)
C(14)-C(13)-C(15)	108.4(3)	C(20)-C(17)-P(2)	113.0(2)
C(16)-C(13)-P(2)	111.7(2)	C(19)-C(17)-P(2)	112.1(2)
C(14)-C(13)-P(2)	105.5(2)	C(18)-C(17)-P(2)	104.3(2)
C(15)-C(13)-P(2)	112.4(2)	C(25)-C(26)-C(27)	111.0(10)
O(1)-C(22)-O(2)	122.4(3)	C(28)-C(27)-C(26)	106.1(9)
O(1)-C(22)-C(21)	127.0(3)	C(27)-C(28)-C(29)	111.5(11)
O(2)-C(22)-C(21)	110.6(3)	C(27A)-C(26A)-C(25A)	110(2)
C(12)-C(11)-N(1)	122.6(3)	C(26A)-C(27A)-C(28A)	104.2(19)
C(10)-C(7)-C(8)	109.8(3)	C(29A)-C(28A)-C(27A)	106(2)

Table S3. Torsion angles [°] for [Pt{C(N_2)CO_2Et}(PNP)] (1).

C(12)-P(2)-C(13)-C(16)	-165.5(2)	C(23)-O(2)-C(22)-C(21)	177.4(3)
C(17)-P(2)-C(13)-C(16)	-51.2(3)	N(2)-C(21)-C(22)-O(1)	-2.4(5)
Pt(1)-P(2)-C(13)-C(16)	86.3(2)	Pt(1)-C(21)-C(22)-O(1)	171.9(3)
C(12)-P(2)-C(13)-C(14)	75.8(2)	N(2)-C(21)-C(22)-O(2)	178.9(3)
C(17)-P(2)-C(13)-C(14)	-169.9(2)	Pt(1)-C(21)-C(22)-O(2)	-6.7(4)
Pt(1)-P(2)-C(13)-C(14)	-32.5(2)	C(1)-N(1)-C(11)-C(12)	178.5(3)
C(12)-P(2)-C(13)-C(15)	-42.1(3)	Pt(1)-N(1)-C(11)-C(12)	1.0(4)
C(17)-P(2)-C(13)-C(15)	72.2(3)	C(2)-P(1)-C(7)-C(10)	-169.9(2)
Pt(1)-P(2)-C(13)-C(15)	-150.4(2)	C(3)-P(1)-C(7)-C(10)	-55.8(3)
C(23)-O(2)-C(22)-O(1)	-1.3(4)	Pt(1)-P(1)-C(7)-C(10)	80.1(2)

C(2)-P(1)-C(7)-C(8)	-45.5(3)	C(3)-P(1)-C(2)-C(1)	116.6(3)
C(3)-P(1)-C(7)-C(8)	68.7(3)	Pt(1)-P(1)-C(2)-C(1)	-1.7(3)
Pt(1)-P(1)-C(7)-C(8)	-155.4(2)	N(1)-C(11)-C(12)-P(2)	-0.6(4)
C(2)-P(1)-C(7)-C(9)	71.8(2)	C(17)-P(2)-C(12)-C(11)	121.6(3)
C(3)-P(1)-C(7)-C(9)	-174.0(2)	C(13)-P(2)-C(12)-C(11)	-118.7(3)
Pt(1)-P(1)-C(7)-C(9)	-38.1(2)	Pt(1)-P(2)-C(12)-C(11)	0.0(3)
C(11)-N(1)-C(1)-C(2)	-176.5(3)	C(12)-P(2)-C(17)-C(20)	-178.2(2)
Pt(1)-N(1)-C(1)-C(2)	1.0(4)	C(13)-P(2)-C(17)-C(20)	67.9(3)
C(22)-O(2)-C(23)-C(24)	75.9(4)	Pt(1)-P(2)-C(17)-C(20)	-68.5(2)
C(2)-P(1)-C(3)-C(5)	60.8(3)	C(12)-P(2)-C(17)-C(19)	58.9(3)
C(7)-P(1)-C(3)-C(5)	-52.9(3)	C(13)-P(2)-C(17)-C(19)	-55.1(3)
Pt(1)-P(1)-C(3)-C(5)	169.6(3)	Pt(1)-P(2)-C(17)-C(19)	168.5(2)
C(2)-P(1)-C(3)-C(6)	-176.4(3)	C(12)-P(2)-C(17)-C(18)	-60.5(2)
C(7)-P(1)-C(3)-C(6)	69.9(3)	C(13)-P(2)-C(17)-C(18)	-174.5(2)
Pt(1)-P(1)-C(3)-C(6)	-67.6(3)	Pt(1)-P(2)-C(17)-C(18)	49.2(2)
C(2)-P(1)-C(3)-C(4)	-59.1(3)	C(25)-C(26)-C(27)-C(28)	162.4(13)
C(7)-P(1)-C(3)-C(4)	-172.8(2)	C(26)-C(27)-C(28)-C(29)	177.7(13)
Pt(1)-P(1)-C(3)-C(4)	49.7(3)	C(25A)-C(26A)-C(27A)-C(28A)	179(4)
N(1)-C(1)-C(2)-P(1)	0.7(4)	C(26A)-C(27A)-C(28A)-C(29A)	128(4)
C(7)-P(1)-C(2)-C(1)	-123.6(3)		

2.4 Crystal Structure of [Pt(CHO)(PNP)] (2)

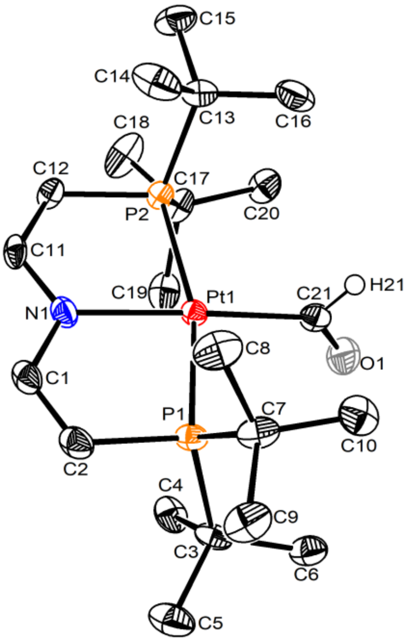

Figure S26. Asymmetric unit of [Pt(CHO)(PNP)] (**2**) with 50% thermal ellipsoids, CCDC deposition number: 2409643. H atoms are omitted for clarity. The asymmetric unit contains one complex molecule.

Table S4. Crystal data and structure refinement for [Pt(CHO)(PNP)] (2).

Identification code	2
Empirical formula	$C_{21}H_{41}NOP_2Pt$
Formula weight	580.58
Temperature/K	100.0
Crystal system	monoclinic
Space group	$P2_1/c$
a/Å	8.630(2)
b/Å	11.416(2)
c/Å	24.370(3)
β/°	90.16(2)
Volume/Å^3	2401.0(3)
Z	4
ρ_{calc}g/cm^3	1.606
μ/mm^{-1}	5.988
F(000)	1160.0
Crystal size/mm	0.12 × 0.1 × 0.084
Radiation	Mo$K\alpha$ (λ = 0.71073)
2Θ range for data collection/°	3.94 to 56.596
Index ranges	$-11 \le h \le 11$, $-15 \le k \le 15$, $-32 \le l \le 32$
Reflections collected	65077
Independent reflections	5962 [R_{int} = 0.1083, R_{sigma} = 0.0519]
Data/restraints/parameters	5962 / 0 / 248

Goodness-of-fit on F^2	1.049
Final R indexes [$I \geq 2\sigma(I)$]	R_1 = 0.0324, wR_2 = 0.0622
Final R indexes [all data]	R_1 = 0.0488, wR_2 = 0.0665
Extinction coefficient	0.00039(7)
Largest diff. peak/hole / e Å^{-3}	0.934 / -0.774

Table S5. Bond lengths [Å] and angles [°] for [Pt(CHO)(PNP)] (2).

Pt(1)-C(21)	1.997(5)	P(2)-Pt(1)-P(1)	164.85(4)
Pt(1)-N(1)	2.069(4)	C(2)-P(1)-C(7)	107.8(2)
Pt(1)-P(2)	2.2989(11)	C(2)-P(1)-C(3)	105.6(2)
Pt(1)-P(1)	2.3012(11)	C(7)-P(1)-C(3)	113.2(2)
P(1)-C(2)	1.777(5)	C(2)-P(1)-Pt(1)	100.36(15)
P(1)-C(7)	1.877(4)	C(7)-P(1)-Pt(1)	114.10(14)
P(1)-C(3)	1.877(4)	C(3)-P(1)-Pt(1)	114.33(14)
P(2)-C(12)	1.773(4)	C(12)-P(2)-C(13)	106.2(2)
P(2)-C(13)	1.875(5)	C(12)-P(2)-C(17)	106.4(2)
P(2)-C(17)	1.875(4)	C(13)-P(2)-C(17)	113.1(2)
O(1)-C(21)	1.216(5)	C(12)-P(2)-Pt(1)	100.53(15)
N(1)-C(1)	1.366(5)	C(13)-P(2)-Pt(1)	114.23(15)
N(1)-C(11)	1.377(5)	C(17)-P(2)-Pt(1)	114.83(15)
C(1)-C(2)	1.343(6)	C(1)-N(1)-C(11)	121.8(4)
C(3)-C(6)	1.521(7)	C(1)-N(1)-Pt(1)	119.1(3)
C(3)-C(5)	1.537(6)	C(11)-N(1)-Pt(1)	118.9(3)
C(3)-C(4)	1.539(6)	C(2)-C(1)-N(1)	121.7(4)
C(7)-C(10)	1.530(7)	C(1)-C(2)-P(1)	116.4(4)
C(7)-C(8)	1.530(6)	C(6)-C(3)-C(5)	109.7(4)
C(7)-C(9)	1.547(6)	C(6)-C(3)-C(4)	109.8(4)
C(11)-C(12)	1.347(6)	C(5)-C(3)-C(4)	108.7(4)
C(13)-C(16)	1.517(7)	C(6)-C(3)-P(1)	112.3(3)
C(13)-C(15)	1.522(6)	C(5)-C(3)-P(1)	112.5(3)
C(13)-C(14)	1.536(6)	C(4)-C(3)-P(1)	103.6(3)
C(17)-C(19)	1.517(7)	C(10)-C(7)-C(8)	109.5(4)
C(17)-C(20)	1.528(6)	C(10)-C(7)-C(9)	109.8(4)
C(17)-C(18)	1.538(6)	C(8)-C(7)-C(9)	107.9(4)
		C(10)-C(7)-P(1)	111.5(3)
C(21)-Pt(1)-N(1)	176.37(16)	C(8)-C(7)-P(1)	105.2(3)
C(21)-Pt(1)-P(2)	97.46(12)	C(9)-C(7)-P(1)	112.8(3)
N(1)-Pt(1)-P(2)	82.48(10)	C(12)-C(11)-N(1)	121.4(4)
C(21)-Pt(1)-P(1)	97.67(12)	C(11)-C(12)-P(2)	116.4(3)
N(1)-Pt(1)-P(1)	82.38(10)	C(16)-C(13)-C(15)	110.4(4)

C(16)-C(13)-C(14)	108.4(4)	C(19)-C(17)-C(18)	108.8(4)
C(15)-C(13)-C(14)	108.9(4)	C(20)-C(17)-C(18)	109.6(4)
C(16)-C(13)-P(2)	110.9(3)	C(19)-C(17)-P(2)	105.3(3)
C(15)-C(13)-P(2)	113.1(3)	C(20)-C(17)-P(2)	111.5(3)
C(14)-C(13)-P(2)	104.8(3)	C(18)-C(17)-P(2)	112.5(3)
C(19)-C(17)-C(20)	108.9(4)	O(1)-C(21)-Pt(1)	128.0(4)

Table S6. Torsion angles [°] for [Pt(CHO)(PNP)] (2).

C(11)-N(1)-C(1)-C(2)	177.1(4)	C(1)-N(1)-C(11)-C(12)	-177.7(4)
Pt(1)-N(1)-C(1)-C(2)	1.6(6)	Pt(1)-N(1)-C(11)-C(12)	-2.1(5)
N(1)-C(1)-C(2)-P(1)	-1.7(6)	N(1)-C(11)-C(12)-P(2)	-2.1(6)
C(7)-P(1)-C(2)-C(1)	120.7(4)	C(13)-P(2)-C(12)-C(11)	-114.8(4)
C(3)-P(1)-C(2)-C(1)	-118.0(4)	C(17)-P(2)-C(12)-C(11)	124.5(3)
Pt(1)-P(1)-C(2)-C(1)	1.0(4)	Pt(1)-P(2)-C(12)-C(11)	4.5(4)
C(2)-P(1)-C(3)-C(6)	-168.6(3)	C(12)-P(2)-C(13)-C(16)	165.8(3)
C(7)-P(1)-C(3)-C(6)	-50.9(4)	C(17)-P(2)-C(13)-C(16)	-77.9(4)
Pt(1)-P(1)-C(3)-C(6)	82.1(3)	Pt(1)-P(2)-C(13)-C(16)	55.9(4)
C(2)-P(1)-C(3)-C(5)	-44.2(4)	C(12)-P(2)-C(13)-C(15)	-69.5(4)
C(7)-P(1)-C(3)-C(5)	73.5(4)	C(17)-P(2)-C(13)-C(15)	46.8(4)
Pt(1)-P(1)-C(3)-C(5)	-153.6(3)	Pt(1)-P(2)-C(13)-C(15)	-179.4(3)
C(2)-P(1)-C(3)-C(4)	73.1(3)	C(12)-P(2)-C(13)-C(14)	49.0(4)
C(7)-P(1)-C(3)-C(4)	-169.2(3)	C(17)-P(2)-C(13)-C(14)	165.4(3)
Pt(1)-P(1)-C(3)-C(4)	-36.3(3)	Pt(1)-P(2)-C(13)-C(14)	-60.8(4)
C(2)-P(1)-C(7)-C(10)	-172.6(3)	C(12)-P(2)-C(17)-C(19)	-77.3(3)
C(3)-P(1)-C(7)-C(10)	71.0(4)	C(13)-P(2)-C(17)-C(19)	166.5(3)
Pt(1)-P(1)-C(7)-C(10)	-62.1(3)	Pt(1)-P(2)-C(17)-C(19)	32.9(3)
C(2)-P(1)-C(7)-C(8)	-54.0(4)	C(12)-P(2)-C(17)-C(20)	164.7(3)
C(3)-P(1)-C(7)-C(8)	-170.4(3)	C(13)-P(2)-C(17)-C(20)	48.5(4)
Pt(1)-P(1)-C(7)-C(8)	56.5(3)	Pt(1)-P(2)-C(17)-C(20)	-85.1(3)
C(2)-P(1)-C(7)-C(9)	63.4(4)	C(12)-P(2)-C(17)-C(18)	41.1(4)
C(3)-P(1)-C(7)-C(9)	-53.1(4)	C(13)-P(2)-C(17)-C(18)	-75.1(4)
Pt(1)-P(1)-C(7)-C(9)	173.9(3)	Pt(1)-P(2)-C(17)-C(18)	151.3(3)

2.5 Crystal Structure of [Pd{C(CO)OEt}(PNP)] (3)

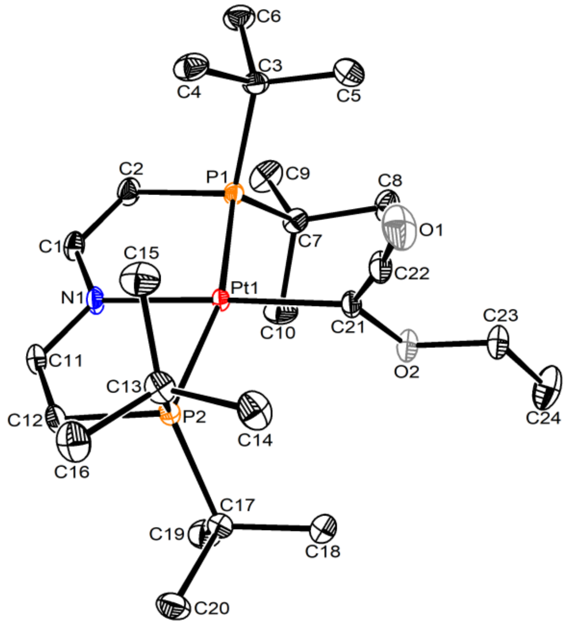

Figure S27. Asymmetric unit of [Pd{C(CO)OEt}(PNP)] (**3**) with 50% thermal ellipsoids, CCDC deposition number: 2409644. H atoms are omitted for clarity. The asymmetric unit contains one complex molecule.

Table S7. Crystal data and structure refinement for [Pd{C(CO)OEt}(PNP)].

Identification code	3
Empirical formula	$C_{24}H_{45}NO_2P_2Pt$
Formula weight	636.64
Temperature/K	100.00
Crystal system	monoclinic
Space group	$P2_1/n$
a/Å	8.926(2)
b/Å	18.295(3)
c/Å	16.512(2)
β/°	92.49(2)
Volume/Å^3	2693.9(8)
Z	4
ρ_{calc}g/cm^3	1.570
μ/mm^{-1}	5.347
F(000)	1280.0
Crystal size/mm	0.177 × 0.159 × 0.069
Radiation	Mo$K\alpha$ (λ = 0.71073)
2Θ range for data collection/°	4.452 to 56.654
Index ranges	$-11 \le h \le 11$, $-24 \le k \le 24$, $-22 \le l \le 22$
Reflections collected	132257
Independent reflections	6690 [R_{int} = 0.0331, R_{sigma} = 0.0110]
Data/restraints/parameters	6690/ 0 /285

Goodness-of-fit on F^2	1.171
Final R indexes [$I \geq 2\sigma(I)$]	R_1 = 0.0125, wR_2 = 0.0297
Final R indexes [all data]	R_1 = 0.0127 wR_2 = 0.0298
Extinction coefficient	0.00078(6)
Largest diff. peak/hole / e Å^{-3}	0.873/-0.380

Table S8. Bond lengths [Å] and angles [°] for [Pd{C(CO)OEt}(PNP)].

Pt(1)-C(21)	2.0376(14)	C(21)-Pt(1)-P(2)	97.48(4)
Pt(1)-N(1)	2.0404(12)	N(1)-Pt(1)-P(2)	82.77(4)
Pt(1)-P(2)	2.3008(5)	C(21)-Pt(1)-P(1)	96.76(4)
Pt(1)-P(1)	2.3127(5)	N(1)-Pt(1)-P(1)	83.00(4)
P(1)-C(2)	1.7875(16)	P(2)-Pt(1)-P(1)	165.755(13)
P(1)-C(7)	1.8744(16)	C(2)-P(1)-C(7)	104.57(7)
P(1)-C(3)	1.8750(16)	C(2)-P(1)-C(3)	105.78(7)
P(2)-C(12)	1.7843(15)	C(7)-P(1)-C(3)	113.76(7)
P(2)-C(17)	1.8747(16)	C(2)-P(1)-Pt(1)	99.58(5)
P(2)-C(13)	1.8826(16)	C(7)-P(1)-Pt(1)	116.69(5)
O(1)-C(22)	1.187(2)	C(3)-P(1)-Pt(1)	114.17(5)
O(2)-C(21)	1.4016(18)	C(12)-P(2)-C(17)	105.52(7)
O(2)-C(23)	1.4240(18)	C(12)-P(2)-C(13)	104.90(7)
N(1)-C(11)	1.3633(19)	C(17)-P(2)-C(13)	113.12(7)
N(1)-C(1)	1.3648(19)	C(12)-P(2)-Pt(1)	100.17(5)
C(1)-C(2)	1.348(2)	C(17)-P(2)-Pt(1)	114.71(5)
C(3)-C(5)	1.532(2)	C(13)-P(2)-Pt(1)	116.31(5)
C(3)-C(6)	1.534(2)	C(21)-O(2)-C(23)	112.90(11)
C(3)-C(4)	1.540(2)	C(11)-N(1)-C(1)	121.21(13)
C(7)-C(8)	1.528(2)	C(11)-N(1)-Pt(1)	119.47(10)
C(7)-C(10)	1.536(2)	C(1)-N(1)-Pt(1)	119.28(10)
C(7)-C(9)	1.537(2)	C(2)-C(1)-N(1)	122.33(14)
C(11)-C(12)	1.350(2)	C(1)-C(2)-P(1)	115.78(12)
C(13)-C(14)	1.533(2)	C(5)-C(3)-C(6)	109.48(14)
C(13)-C(16)	1.537(2)	C(5)-C(3)-C(4)	108.36(14)
C(13)-C(15)	1.540(2)	C(6)-C(3)-C(4)	108.99(13)
C(17)-C(20)	1.529(2)	C(5)-C(3)-P(1)	112.25(11)
C(17)-C(18)	1.534(2)	C(6)-C(3)-P(1)	112.39(11)
C(17)-C(19)	1.538(2)	C(4)-C(3)-P(1)	105.16(11)
C(21)-C(22)	1.303(2)	C(8)-C(7)-C(10)	108.90(14)
C(23)-C(24)	1.507(2)	C(8)-C(7)-C(9)	109.18(13)
		C(10)-C(7)-C(9)	108.87(14)
C(21)-Pt(1)-N(1)	176.44(5)	C(8)-C(7)-P(1)	112.94(11)

C(10)-C(7)-P(1)	104.47(10)	C(20)-C(17)-C(19)	109.65(13)
C(9)-C(7)-P(1)	112.27(11)	C(18)-C(17)-C(19)	107.89(14)
C(12)-C(11)-N(1)	122.17(14)	C(20)-C(17)-P(2)	112.10(11)
C(11)-C(12)-P(2)	115.35(11)	C(18)-C(17)-P(2)	112.22(11)
C(14)-C(13)-C(16)	109.83(13)	C(19)-C(17)-P(2)	105.04(11)
C(14)-C(13)-C(15)	109.08(14)	C(22)-C(21)-O(2)	122.48(13)
C(16)-C(13)-C(15)	108.08(14)	C(22)-C(21)-Pt(1)	118.96(11)
C(14)-C(13)-P(2)	112.63(11)	O(2)-C(21)-Pt(1)	118.56(10)
C(16)-C(13)-P(2)	111.94(11)	O(1)-C(22)-C(21)	173.19(17)
C(15)-C(13)-P(2)	105.05(11)	O(2)-C(23)-C(24)	108.23(14)
C(20)-C(17)-C(18)	109.75(13)		

Table S9. Torsion angles [°] for [Pd{C(CO)OEt}(PNP)].

C(11)-N(1)-C(1)-C(2)	-179.27(15)	N(1)-C(11)-C(12)-P(2)	0.0(2)
Pt(1)-N(1)-C(1)-C(2)	-1.5(2)	C(17)-P(2)-C(12)-C(11)	-121.02(13)
N(1)-C(1)-C(2)-P(1)	1.8(2)	C(13)-P(2)-C(12)-C(11)	119.28(13)
C(7)-P(1)-C(2)-C(1)	119.76(13)	Pt(1)-P(2)-C(12)-C(11)	-1.64(13)
C(3)-P(1)-C(2)-C(1)	-119.84(13)	C(12)-P(2)-C(13)-C(14)	161.71(12)
Pt(1)-P(1)-C(2)-C(1)	-1.20(13)	C(17)-P(2)-C(13)-C(14)	47.20(14)
C(2)-P(1)-C(3)-C(5)	-179.76(12)	Pt(1)-P(2)-C(13)-C(14)	-88.69(12)
C(7)-P(1)-C(3)-C(5)	-65.55(13)	C(12)-P(2)-C(13)-C(16)	37.37(13)
Pt(1)-P(1)-C(3)-C(5)	71.80(12)	C(17)-P(2)-C(13)-C(16)	-77.13(13)
C(2)-P(1)-C(3)-C(6)	-55.82(13)	Pt(1)-P(2)-C(13)-C(16)	146.97(10)
C(7)-P(1)-C(3)-C(6)	58.38(13)	C(12)-P(2)-C(13)-C(15)	-79.68(12)
Pt(1)-P(1)-C(3)-C(6)	-164.27(9)	C(17)-P(2)-C(13)-C(15)	165.82(11)
C(2)-P(1)-C(3)-C(4)	62.63(12)	Pt(1)-P(2)-C(13)-C(15)	29.92(12)
C(7)-P(1)-C(3)-C(4)	176.83(10)	C(12)-P(2)-C(17)-C(20)	-62.85(12)
Pt(1)-P(1)-C(3)-C(4)	-45.82(12)	C(13)-P(2)-C(17)-C(20)	51.28(13)
C(2)-P(1)-C(7)-C(8)	166.23(12)	Pt(1)-P(2)-C(17)-C(20)	-172.09(9)
C(3)-P(1)-C(7)-C(8)	51.31(13)	C(12)-P(2)-C(17)-C(18)	173.11(12)
Pt(1)-P(1)-C(7)-C(8)	-84.91(12)	C(13)-P(2)-C(17)-C(18)	-72.76(13)
C(2)-P(1)-C(7)-C(10)	-75.57(12)	Pt(1)-P(2)-C(17)-C(18)	63.87(12)
C(3)-P(1)-C(7)-C(10)	169.51(10)	C(12)-P(2)-C(17)-C(19)	56.16(12)
Pt(1)-P(1)-C(7)-C(10)	33.29(12)	C(13)-P(2)-C(17)-C(19)	170.29(10)
C(2)-P(1)-C(7)-C(9)	42.25(14)	Pt(1)-P(2)-C(17)-C(19)	-53.08(11)
C(3)-P(1)-C(7)-C(9)	-72.67(14)	C(23)-O(2)-C(21)-C(22)	-2.4(2)
Pt(1)-P(1)-C(7)-C(9)	151.10(10)	C(23)-O(2)-C(21)-Pt(1)	178.02(10)
C(1)-N(1)-C(11)-C(12)	179.90(15)	C(21)-O(2)-C(23)-C(24)	179.32(14)
Pt(1)-N(1)-C(11)-C(12)	2.1(2)		

2.6 Crystal Structure of [Pt(CCO$_2$Et)(PNP)] (4)

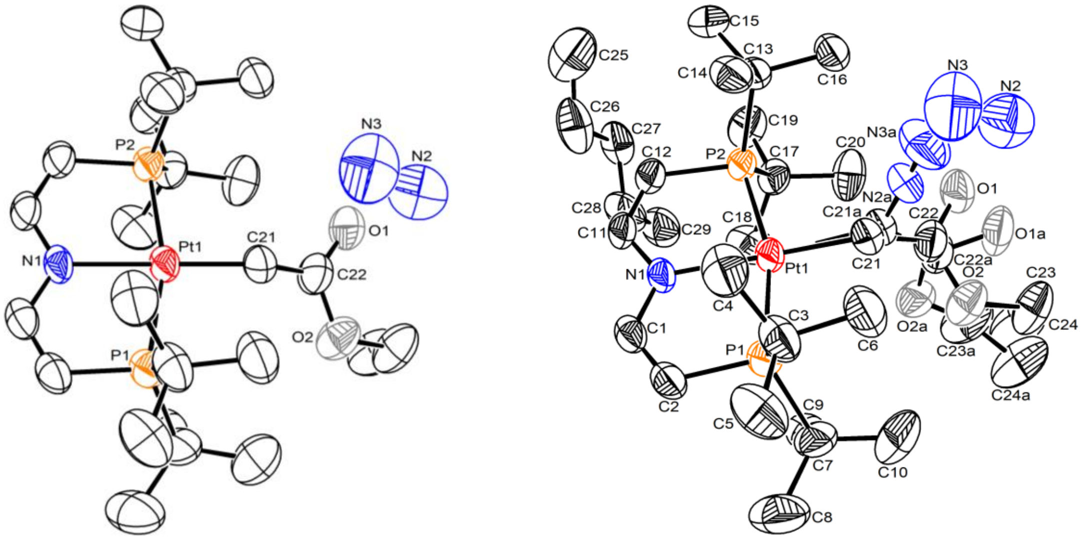

Figure S28. Crystal Structure of [Pt(CCO$_2$Et)(PNP)] (**4**) obtained by crystal-to-crystal reansformation. Left: Solid-state structure of complex **4** at 50% ellipsoid probability; Right: Asymmetric unit of the whole crystal with 50% thermal ellipsoids. CCDC deposition number: 2409646. H atoms are omitted for clarity. The crystal used in this picture was obtained by *in situ* photolysis of **1** which resulted in the expulsion of N_2. The asymmetric unit contains one molecule as two superimposed species, as well as free N_2 and a co-crystallized pentane molecule. The two molecules were refined with occupation factors of 0.642(8) for [Pt(CCO$_2$Et)(PNP)]*N_2 and 0.358(8) for [Pt(C(N_2)CO$_2$Et)(PNP)]. The pentane molecule is disordered about an inversion centre. The structure was refined using some restraints and constraints (RIGU, SIMU, DFIX, EADP, SADI).

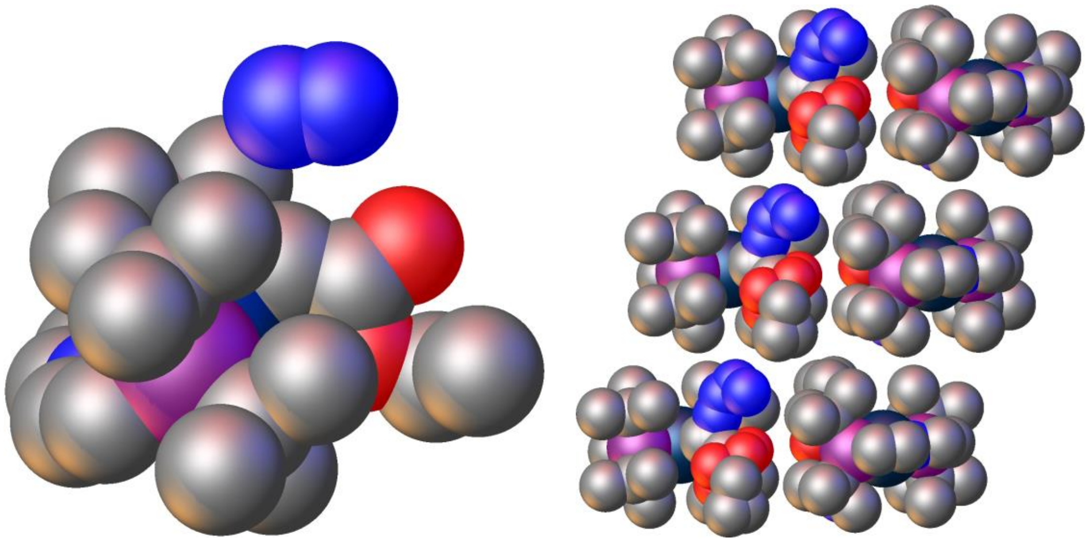

Figure S29. Space filling representation and packing diagram of [Pt(CCO$_2$Et)(PNP)] (**4**) obtained by *in situ* photolysis of **1**. Left: [Pt(CCO$_2$Et)(PNP)]*N_2, H atoms omitted. This space

filling plot shows that there are no close contacts between the free N_2 and the ester group. Right: Crystal packing diagram with six asymmetric units, H atoms and solvent molecules omitted.

Table S10. Crystal data and structure refinement for [Pt(CCO$_2$Et)(PNP)] (4).

Identification code	4
Empirical formula	$C_{26.5}H_{51}N_3O_2P_2Pt$
Formula weight	700.73
Temperature/K	80(2)
Crystal system	triclinic
Space group	*P*-1
a/Å	8.790(2)
b/Å	13.840(3)
c/Å	14.777(3)
α/°	117.80(3)
β/°	97.39(2)
γ/°	92.99(2)
Volume/Å^3	1564.0(7)
Z	2
ρ_{calc}g/cm^3	1.488
μ/mm^{-1}	4.614
F(000)	710.0
Crystal size/mm	0.158 × 0.104 × 0.076
Radiation	Mo$K\alpha$ (λ = 0.71073)
2Θ range for data collection/°	5.424 to 50.056
Index ranges	$-10 \le h \le 10$, $-16 \le k \le 16$, $-17 \le l \le 17$
Reflections collected	40168
Independent reflections	5513 [R_{int} = 0.0631, R_{sigma} = 0.0353]
Data/restraints/parameters	5513 / 346 / 409
Goodness-of-fit on F^2	1.054
Final R indexes [$I \geq 2\sigma(I)$]	R_1 = 0.0421, wR_2 = 0.1050
Final R indexes [all data]	R_1 = 0.0545, wR_2 = 0.1133
Largest diff. peak/hole / e Å^{-3}	2.481 / -1.218

Table S11. Bond lengths [Å] and angles [°] for [Pt(CCO$_2$Et)(PNP)] (4).

Pt(1)-C(21)	1.87(2)	N(1)-C(1)	1.363(10)
Pt(1)-N(1)	2.069(6)	C(1)-C(2)	1.324(12)
Pt(1)-C(21A)	2.12(3)	P(2)-C(12)	1.785(7)
Pt(1)-P(1)	2.315(2)	P(2)-C(13)	1.869(7)
Pt(1)-P(2)	2.317(2)	P(2)-C(17)	1.877(8)
P(1)-C(2)	1.790(8)	C(3)-C(4)	1.521(13)
P(1)-C(7)	1.865(8)	C(3)-C(5)	1.535(13)
P(1)-C(3)	1.879(10)	C(3)-C(6)	1.556(12)
N(1)-C(11)	1.357(10)	C(7)-C(10)	1.529(15)

C(7)-C(9)	1.533(15)
C(7)-C(8)	1.548(14)
C(11)-C(12)	1.344(11)
C(13)-C(15)	1.518(12)
C(13)-C(14)	1.535(11)
C(13)-C(16)	1.535(10)
C(17)-C(19)	1.512(12)
C(17)-C(20)	1.519(11)
C(17)-C(18)	1.521(12)
N(2)-N(3)	1.082(14)
C(21)-C(22)	1.378(14)
C(22)-O(1)	1.23(2)
C(22)-O(2)	1.37(3)
O(2)-C(23)	1.490(15)
C(23)-C(24)	1.42(2)
N(2A)-N(3A)	1.104(15)
N(2A)-C(21A)	1.27(5)
C(21A)-C(22A)	1.381(18)
C(22A)-O(1A)	1.22(2)
C(22A)-O(2A)	1.36(3)
O(2A)-C(23A)	1.50(2)
C(23A)-C(24A)	1.41(3)
C(25)-C(26)	1.542(17)
C(26)-C(27)	1.533(16)
C(27)-C(28)	1.542(16)
C(28)-C(29)	1.539(17)
C(21)-Pt(1)-N(1)	175.2(9)
N(1)-Pt(1)-C(21A)	176.2(11)
C(21)-Pt(1)-P(1)	95.3(10)
N(1)-Pt(1)-P(1)	81.9(2)
C(21A)-Pt(1)-P(1)	100.6(18)
C(21)-Pt(1)-P(2)	100.7(10)
N(1)-Pt(1)-P(2)	82.0(2)
C(21A)-Pt(1)-P(2)	95.4(18)
P(1)-Pt(1)-P(2)	163.93(7)
C(2)-P(1)-C(7)	105.8(4)
C(2)-P(1)-C(3)	105.6(4)
C(7)-P(1)-C(3)	113.9(5)
C(2)-P(1)-Pt(1)	99.9(3)
C(7)-P(1)-Pt(1)	116.7(3)
C(3)-P(1)-Pt(1)	113.0(3)
C(11)-N(1)-C(1)	120.7(6)
C(11)-N(1)-Pt(1)	119.6(5)
C(1)-N(1)-Pt(1)	119.7(6)
C(2)-C(1)-N(1)	122.0(7)
C(12)-P(2)-C(13)	105.7(3)
C(12)-P(2)-C(17)	105.2(4)
C(13)-P(2)-C(17)	114.1(4)
C(12)-P(2)-Pt(1)	100.1(3)
C(13)-P(2)-Pt(1)	114.5(3)
C(17)-P(2)-Pt(1)	115.1(3)
C(1)-C(2)-P(1)	116.5(6)
C(4)-C(3)-C(5)	110.1(8)
C(4)-C(3)-C(6)	107.8(8)
C(5)-C(3)-C(6)	109.6(9)
C(4)-C(3)-P(1)	105.3(7)
C(5)-C(3)-P(1)	112.2(7)
C(6)-C(3)-P(1)	111.6(6)
C(10)-C(7)-C(9)	110.2(9)
C(10)-C(7)-C(8)	111.6(10)
C(9)-C(7)-C(8)	107.4(9)
C(10)-C(7)-P(1)	111.2(7)
C(9)-C(7)-P(1)	105.1(6)
C(8)-C(7)-P(1)	111.0(7)
C(12)-C(11)-N(1)	122.1(7)
C(11)-C(12)-P(2)	116.0(6)
C(15)-C(13)-C(14)	108.2(7)
C(15)-C(13)-C(16)	110.9(7)
C(14)-C(13)-C(16)	109.1(7)
C(15)-C(13)-P(2)	112.8(6)
C(14)-C(13)-P(2)	104.8(5)
C(16)-C(13)-P(2)	110.8(5)
C(19)-C(17)-C(20)	108.9(8)
C(19)-C(17)-C(18)	109.9(8)
C(20)-C(17)-C(18)	108.5(8)
C(19)-C(17)-P(2)	112.6(6)
C(20)-C(17)-P(2)	111.5(6)

C(18)-C(17)-P(2)	105.3(6)	C(22A)-C(21A)-Pt(1)	131(3)
C(22)-C(21)-Pt(1)	141(2)	O(1A)-C(22A)-O(2A)	116(2)
O(1)-C(22)-O(2)	121.2(12)	O(1A)-C(22A)-C(21A)	131(3)
O(1)-C(22)-C(21)	124.8(19)	O(2A)-C(22A)-C(21A)	111(3)
O(2)-C(22)-C(21)	113(2)	C(22A)-O(2A)-C(23A)	118(2)
C(22)-O(2)-C(23)	114.4(12)	C(24A)-C(23A)-O(2A)	113(2)
C(24)-C(23)-O(2)	113.3(16)	C(27)-C(26)-C(25)	117.5(19)
N(3A)-N(2A)-C(21A)	178(4)	C(26)-C(27)-C(28)	116.4(14)
N(2A)-C(21A)-C(22A)	107(3)	C(29)-C(28)-C(27)	116.9(16)
N(2A)-C(21A)-Pt(1)	122.1(16)		

Table S12. Torsion angles [°] for [Pt(CCO$_2$Et)(PNP)] (4).

C(11)-N(1)-C(1)-C(2)	-177.7(7)	N(1)-C(11)-C(12)-P(2)	1.0(10)
Pt(1)-N(1)-C(1)-C(2)	0.9(9)	C(13)-P(2)-C(12)-C(11)	-122.1(6)
N(1)-C(1)-C(2)-P(1)	1.6(10)	C(17)-P(2)-C(12)-C(11)	116.8(7)
C(7)-P(1)-C(2)-C(1)	-124.3(7)	Pt(1)-P(2)-C(12)-C(11)	-2.9(6)
C(3)-P(1)-C(2)-C(1)	114.7(7)	C(12)-P(2)-C(13)-C(15)	-45.6(7)
Pt(1)-P(1)-C(2)-C(1)	-2.7(6)	C(17)-P(2)-C(13)-C(15)	69.6(7)
C(2)-P(1)-C(3)-C(4)	-59.5(7)	Pt(1)-P(2)-C(13)-C(15)	-154.8(5)
C(7)-P(1)-C(3)-C(4)	-175.2(6)	C(12)-P(2)-C(13)-C(14)	71.9(6)
Pt(1)-P(1)-C(3)-C(4)	48.7(6)	C(17)-P(2)-C(13)-C(14)	-172.9(5)
C(2)-P(1)-C(3)-C(5)	60.3(8)	Pt(1)-P(2)-C(13)-C(14)	-37.3(6)
C(7)-P(1)-C(3)-C(5)	-55.4(9)	C(12)-P(2)-C(13)-C(16)	-170.6(6)
Pt(1)-P(1)-C(3)-C(5)	168.5(7)	C(17)-P(2)-C(13)-C(16)	-55.4(7)
C(2)-P(1)-C(3)-C(6)	-176.3(7)	Pt(1)-P(2)-C(13)-C(16)	80.2(6)
C(7)-P(1)-C(3)-C(6)	68.1(8)	C(12)-P(2)-C(17)-C(19)	62.6(7)
Pt(1)-P(1)-C(3)-C(6)	-68.1(7)	C(13)-P(2)-C(17)-C(19)	-52.8(7)
C(2)-P(1)-C(7)-C(10)	-165.8(8)	Pt(1)-P(2)-C(17)-C(19)	171.8(6)
C(3)-P(1)-C(7)-C(10)	-50.3(9)	C(12)-P(2)-C(17)-C(20)	-174.7(7)
Pt(1)-P(1)-C(7)-C(10)	84.2(9)	C(13)-P(2)-C(17)-C(20)	69.9(7)
C(2)-P(1)-C(7)-C(9)	75.0(7)	Pt(1)-P(2)-C(17)-C(20)	-65.5(7)
C(3)-P(1)-C(7)-C(9)	-169.5(6)	C(12)-P(2)-C(17)-C(18)	-57.2(8)
Pt(1)-P(1)-C(7)-C(9)	-35.1(7)	C(13)-P(2)-C(17)-C(18)	-172.6(7)
C(2)-P(1)-C(7)-C(8)	-40.9(10)	Pt(1)-P(2)-C(17)-C(18)	52.1(7)
C(3)-P(1)-C(7)-C(8)	74.6(10)	P(1)-Pt(1)-C(21)-C(22)	109(3)
Pt(1)-P(1)-C(7)-C(8)	-150.9(8)	P(2)-Pt(1)-C(21)-C(22)	-73(3)
C(1)-N(1)-C(11)-C(12)	-179.5(7)	Pt(1)-C(21)-C(22)-O(1)	98(3)
Pt(1)-N(1)-C(11)-C(12)	1.9(10)	Pt(1)-C(21)-C(22)-O(2)	-94(4)

O(1)-C(22)-O(2)-C(23)	-12(3)	Pt(1)-C(21A)-C(22A)-O(2A)	3(8)
C(21)-C(22)-O(2)-C(23)	179.8(19)	O(1A)-C(22A)-O(2A)-C(23A)	2(7)
C(22)-O(2)-C(23)-C(24)	-79(2)	C(21A)-C(22A)-O(2A)-C(23A)	171(4)
N(2A)-C(21A)-C(22A)-O(1A)	-17(10)	C(22A)-O(2A)-C(23A)-C(24A)	69(5)
Pt(1)-C(21A)-C(22A)-O(1A)	170(5)	C(25)-C(26)-C(27)-C(28)	172(3)
N(2A)-C(21A)-C(22A)-O(2A)	177(5)	C(26)-C(27)-C(28)-C(29)	175(3)

2.7 Crystal Structure of 3 from crystal-to-crystal transformation

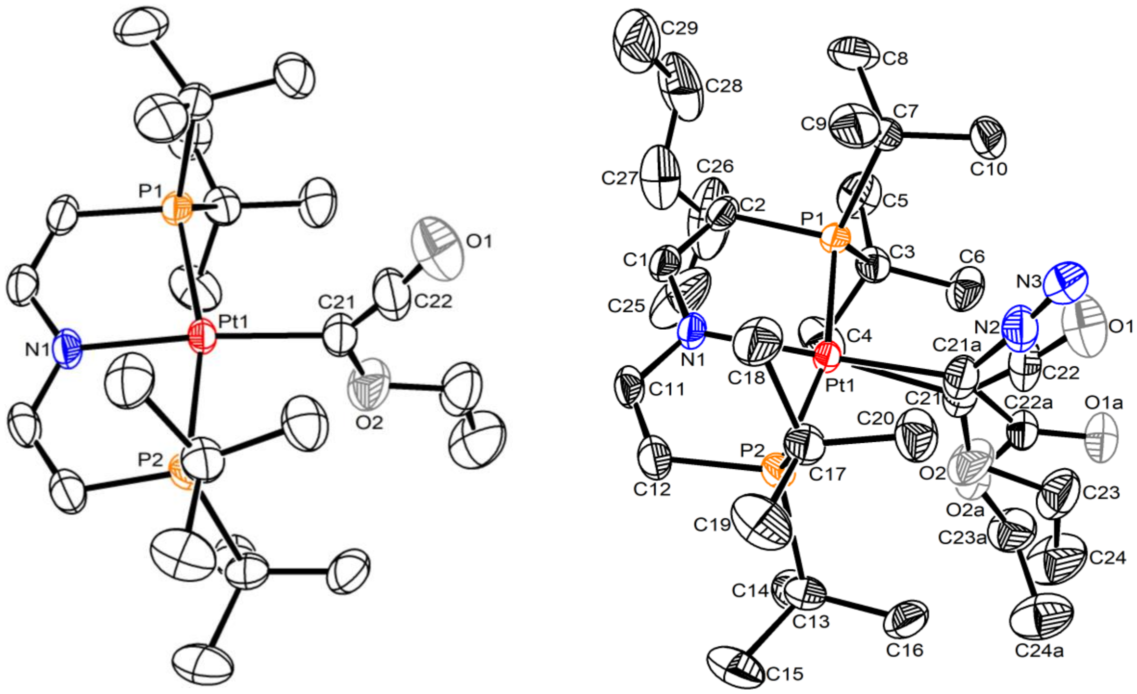

Figure S30. Crystal Structure of **3** obtained by crystal-to-crystal reansformation. Left: solid-state structure of complex **3** from crystal-to-crystal transformation at 50% ellipsoid probability; Right: Asymmetric unit of the whole crystal with 50% thermal ellipsoids. CCDC deposition number: 2409645. H atoms are omitted for clarity. The asymmetric unit contains two different, superimposed molecules and one co-crystallized pentane molecule. The two alternative molecules were refined with occupation factors of 0.722(11) for [Pt(C(CO)OEt)(PNP)] (**3**) and 0.278(11) for [Pt(C(N_2)CO_2Et)(PNP)] (**1**). The pentane molecule is disordered about an inversion centre. The structure was refined using some restraints and constraints (RIGU, SIMU, DFIX, EADP, SADI).

Table S13. Crystal data and structure refinement for 3 from crystal-to-crystal transformation.

Identification code	3
Empirical formula	$C_{26.5}H_{51}N_{1.57}O_2P_2Pt$
Formula weight	680.67
Temperature/K	120(2)
Crystal system	triclinic
Space group	*P*-1
a/Å	8.622(2)
b/Å	13.445(3)
c/Å	14.909(3)
α/°	63.52(2)
β/°	78.08(2)
γ/°	86.02(2)

Volume/Å^3	1513.1(6)
Z	2
ρ_{calc}g/cm^3	1.494
μ/mm^{-1}	4.765
F(000)	690.0
Crystal size/mm	0.126 × 0.118 × 0.075
Radiation	Mo$K\alpha$ (λ = 0.71073)
2Θ range for data collection/°	5.486 to 53.062
Index ranges	$-10 \le h \le 10$, $-16 \le k \le 16$, $-18 \le l \le 18$
Reflections collected	41305
Independent reflections	6245 [R_{int} = 0.0700, R_{sigma} = 0.0428]
Data/restraints/parameters	6245 / 656 / 395
Goodness-of-fit on F^2	1.075
Final R indexes [$I \geq 2\sigma$ (I)]	R_1 = 0.0370, wR_2 = 0.0833
Final R indexes [all data]	R_1 = 0.0488, wR_2 = 0.0883
Largest diff. peak/hole / e Å^{-3}	2.391 / -1.926

Table S14. Bond lengths [Å] and angles [°] for 3 from crystal-to-crystal transformation.

Pt(1)-C(21A)	2.01(5)	C(13)-C(15)	1.545(9)
Pt(1)-N(1)	2.040(4)	C(17)-C(19)	1.514(9)
Pt(1)-C(21)	2.057(16)	C(17)-C(20)	1.527(8)
Pt(1)-P(1)	2.3088(16)	C(17)-C(18)	1.542(8)
Pt(1)-P(2)	2.3111(16)	O(1)-C(22)	1.222(16)
P(1)-C(2)	1.784(6)	O(2)-C(21)	1.415(15)
P(1)-C(3)	1.866(6)	O(2)-C(23)	1.424(13)
P(1)-C(7)	1.868(6)	C(21)-C(22)	1.231(19)
N(1)-C(1)	1.367(8)	C(23)-C(24)	1.531(19)
N(1)-C(11)	1.375(8)	N(2)-N(3)	1.15(4)
C(1)-C(2)	1.342(9)	N(2)-C(21A)	1.34(5)
P(2)-C(12)	1.783(6)	O(1A)-C(22A)	1.19(3)
P(2)-C(13)	1.870(6)	O(2A)-C(22A)	1.40(4)
P(2)-C(17)	1.874(6)	O(2A)-C(23A)	1.45(2)
C(3)-C(4)	1.524(8)	C(21A)-C(22A)	1.49(5)
C(3)-C(5)	1.525(9)	C(23A)-C(24A)	1.52(2)
C(3)-C(6)	1.527(8)	C(25)-C(26)	1.557(17)
C(7)-C(8)	1.525(9)	C(26)-C(27)	1.566(15)
C(7)-C(9)	1.535(9)	C(27)-C(28)	1.577(16)
C(7)-C(10)	1.536(8)	C(28)-C(29)	1.544(16)
C(11)-C(12)	1.332(9)		
C(13)-C(16)	1.519(9)	C(21A)-Pt(1)-N(1)	177.5(11)
C(13)-C(14)	1.534(9)	N(1)-Pt(1)-C(21)	171.8(4)

C(21A)-Pt(1)-P(1)	97(2)	C(9)-C(7)-P(1)	105.0(4)
N(1)-Pt(1)-P(1)	82.75(15)	C(10)-C(7)-P(1)	112.2(4)
C(21)-Pt(1)-P(1)	97.8(7)	C(12)-C(11)-N(1)	122.3(5)
C(21A)-Pt(1)-P(2)	98(2)	C(11)-C(12)-P(2)	115.8(5)
N(1)-Pt(1)-P(2)	82.62(15)	C(16)-C(13)-C(14)	110.0(5)
C(21)-Pt(1)-P(2)	97.2(7)	C(16)-C(13)-C(15)	110.6(6)
P(1)-Pt(1)-P(2)	164.98(5)	C(14)-C(13)-C(15)	108.0(5)
C(2)-P(1)-C(3)	105.8(3)	C(16)-C(13)-P(2)	110.9(4)
C(2)-P(1)-C(7)	104.8(3)	C(14)-C(13)-P(2)	105.0(4)
C(3)-P(1)-C(7)	113.5(3)	C(15)-C(13)-P(2)	112.1(5)
C(2)-P(1)-Pt(1)	99.9(2)	C(19)-C(17)-C(20)	108.8(6)
C(3)-P(1)-Pt(1)	116.5(2)	C(19)-C(17)-C(18)	109.8(6)
C(7)-P(1)-Pt(1)	114.2(2)	C(20)-C(17)-C(18)	107.5(5)
C(1)-N(1)-C(11)	121.5(5)	C(19)-C(17)-P(2)	113.8(4)
C(1)-N(1)-Pt(1)	119.4(4)	C(20)-C(17)-P(2)	111.3(4)
C(11)-N(1)-Pt(1)	119.1(4)	C(18)-C(17)-P(2)	105.3(4)
C(2)-C(1)-N(1)	122.3(5)	C(21)-O(2)-C(23)	114.4(12)
C(12)-P(2)-C(13)	106.0(3)	C(22)-C(21)-O(2)	122.8(14)
C(12)-P(2)-C(17)	105.6(3)	C(22)-C(21)-Pt(1)	125.1(10)
C(13)-P(2)-C(17)	113.5(3)	O(2)-C(21)-Pt(1)	112.1(10)
C(12)-P(2)-Pt(1)	99.7(2)	O(1)-C(22)-C(21)	173.1(15)
C(13)-P(2)-Pt(1)	116.9(2)	O(2)-C(23)-C(24)	107.8(12)
C(17)-P(2)-Pt(1)	113.18(19)	N(3)-N(2)-C(21A)	173(3)
C(1)-C(2)-P(1)	115.7(5)	C(22A)-O(2A)-C(23A)	123(2)
C(4)-C(3)-C(5)	110.0(6)	N(2)-C(21A)-C(22A)	103(3)
C(4)-C(3)-C(6)	108.2(6)	N(2)-C(21A)-Pt(1)	124(3)
C(5)-C(3)-C(6)	108.1(5)	C(22A)-C(21A)-Pt(1)	132(3)
C(4)-C(3)-P(1)	104.1(4)	O(1A)-C(22A)-O(2A)	115(2)
C(5)-C(3)-P(1)	113.5(4)	O(1A)-C(22A)-C(21A)	135(3)
C(6)-C(3)-P(1)	112.9(4)	O(2A)-C(22A)-C(21A)	110(2)
C(8)-C(7)-C(9)	108.3(6)	O(2A)-C(23A)-C(24A)	108(2)
C(8)-C(7)-C(10)	109.9(6)	C(25)-C(26)-C(27)	114.8(16)
C(9)-C(7)-C(10)	108.6(6)	C(26)-C(27)-C(28)	111.4(13)
C(8)-C(7)-P(1)	112.6(4)	C(29)-C(28)-C(27)	112.5(14)

Table S15. Torsion angles [°] for 3 from crystal-to-crystal transformation.

C(11)-N(1)-C(1)-C(2)	-178.3(5)	Pt(1)-N(1)-C(1)-C(2)	-1.2(7)

N(1)-C(1)-C(2)-P(1)	0.5(8)	C(17)-P(2)-C(13)-C(16)	-54.2(5)
C(3)-P(1)-C(2)-C(1)	121.5(5)	Pt(1)-P(2)-C(13)-C(16)	80.4(5)
C(7)-P(1)-C(2)-C(1)	-118.3(5)	C(12)-P(2)-C(13)-C(14)	71.6(5)
Pt(1)-P(1)-C(2)-C(1)	0.3(5)	C(17)-P(2)-C(13)-C(14)	-173.0(4)
C(2)-P(1)-C(3)-C(4)	-61.3(5)	Pt(1)-P(2)-C(13)-C(14)	-38.5(5)
C(7)-P(1)-C(3)-C(4)	-175.7(5)	C(12)-P(2)-C(13)-C(15)	-45.5(6)
Pt(1)-P(1)-C(3)-C(4)	48.5(5)	C(17)-P(2)-C(13)-C(15)	70.0(6)
C(2)-P(1)-C(3)-C(5)	58.2(5)	Pt(1)-P(2)-C(13)-C(15)	-155.5(4)
C(7)-P(1)-C(3)-C(5)	-56.2(5)	C(12)-P(2)-C(17)-C(19)	66.5(6)
Pt(1)-P(1)-C(3)-C(5)	168.1(4)	C(13)-P(2)-C(17)-C(19)	-49.1(6)
C(2)-P(1)-C(3)-C(6)	-178.4(5)	Pt(1)-P(2)-C(17)-C(19)	174.7(4)
C(7)-P(1)-C(3)-C(6)	67.2(5)	C(12)-P(2)-C(17)-C(20)	-170.0(5)
Pt(1)-P(1)-C(3)-C(6)	-68.5(5)	C(13)-P(2)-C(17)-C(20)	74.3(5)
C(2)-P(1)-C(7)-C(8)	-45.6(6)	Pt(1)-P(2)-C(17)-C(20)	-61.9(5)
C(3)-P(1)-C(7)-C(8)	69.3(6)	C(12)-P(2)-C(17)-C(18)	-53.8(5)
Pt(1)-P(1)-C(7)-C(8)	-154.0(5)	C(13)-P(2)-C(17)-C(18)	-169.4(4)
C(2)-P(1)-C(7)-C(9)	71.9(5)	Pt(1)-P(2)-C(17)-C(18)	54.4(5)
C(3)-P(1)-C(7)-C(9)	-173.1(4)	C(23)-O(2)-C(21)-C(22)	3(3)
Pt(1)-P(1)-C(7)-C(9)	-36.4(5)	C(23)-O(2)-C(21)-Pt(1)	-176.4(10)
C(2)-P(1)-C(7)-C(10)	-170.2(5)	C(21)-O(2)-C(23)-C(24)	-178.7(15)
C(3)-P(1)-C(7)-C(10)	-55.3(6)	C(23A)-O(2A)-C(22A)-O(1A)	-3(4)
Pt(1)-P(1)-C(7)-C(10)	81.4(5)	C(23A)-O(2A)-C(22A)-C(21A)	177(4)
C(1)-N(1)-C(11)-C(12)	-179.6(5)	N(2)-C(21A)-C(22A)-O(1A)	2(7)
Pt(1)-N(1)-C(11)-C(12)	3.3(7)	Pt(1)-C(21A)-C(22A)-O(1A)	172(3)
N(1)-C(11)-C(12)-P(2)	2.0(8)	N(2)-C(21A)-C(22A)-O(2A)	-178(3)
C(13)-P(2)-C(12)-C(11)	-126.9(5)	Pt(1)-C(21A)-C(22A)-O(2A)	-8(6)
C(17)-P(2)-C(12)-C(11)	112.4(5)	C(22A)-O(2A)-C(23A)-C(24A)	80(4)
Pt(1)-P(2)-C(12)-C(11)	-5.2(5)	C(25)-C(26)-C(27)-C(28)	-166(2)
C(12)-P(2)-C(13)-C(16)	-169.6(5)	C(26)-C(27)-C(28)-C(29)	-174.6(19)

3. *In situ* SQUID Measurement

SQUID measurement of **4** was carried out using a Quantum Design MPMS3 SQUID magnetometer. Photoproduct **4** were formed *in situ* via the photolysis of **1** with a TLS120Xe xenon light source (465 nm), using the fiber optical sample holder (FOSH) to get optical access to the sample. For this purpose, small amounts of sample were placed in the FOSH. After insertion of the FOSH in the magnetometer, the samples were centered in the magnetometer coils and cooled to 2 K. In order to obtain a background measurement of the set-up prior to the photoreaction, the magnetic moment of **1** in the FOSH was measured from 2 K to 295 K at a magnetic field of 5000 Oe. Afterwards, the samples were cooled down to 10 K, followed by a temperature stabilization period of 30 minutes. Subsequently, the reaction from **1** to **4** was carried out by photolysis at 465 nm over the course of 1.5 h. Monitoring the reaction indicated no change of the the DC magnetic moment over the whole photolysis time (Figure S31).

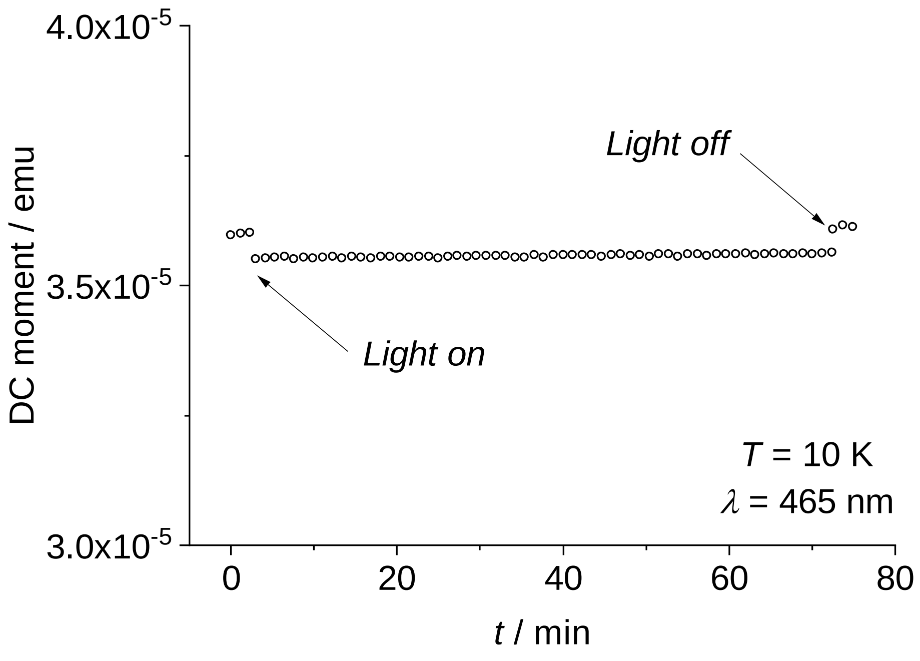

Figure S31. DC magnetic moment *vs* time during photolysis of **1a** with 465 nm light at 10 K.

4. Low-Temperature UV/Vis Spectra Measurement

1 (1.8 mg, 2.7 µmol) is dissolved in 2-MeTHF (2.0 mL) in a reinforced quartz cuvette and sealed with a rubber septum. After freezing the cuvette in liquid nitrogen, the sample is irradiated with a 525 nm LED for 20 minutes. The cuvette is then transferred into the precooled UV/Vis spectrometer (78 K). After measuring the frozen solution (Figure S32), the sample is warmed to 123 K and a spectrum is measured after 5 minutes of thermal equilibration. Further spectra was measured at 153 K.

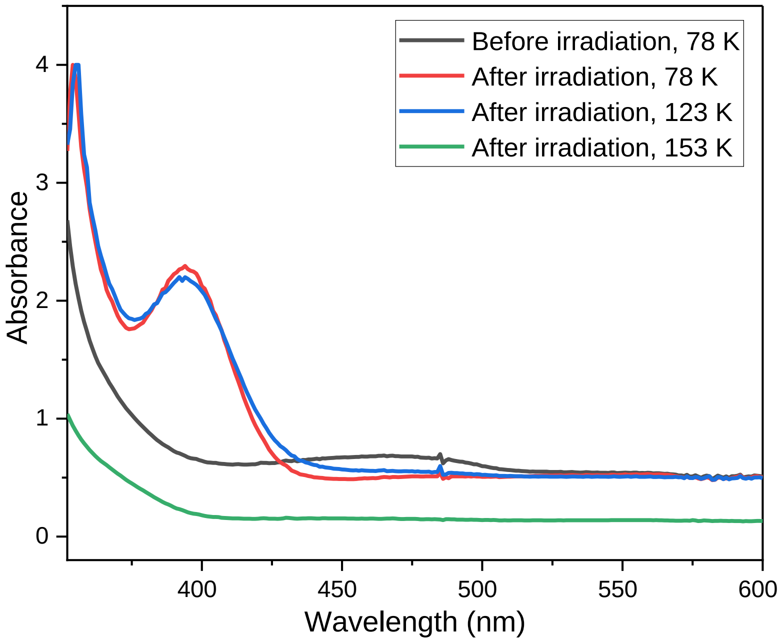

Figure S32. UV/Vis spectra of **1** before (black line) and after (red line) photolysis at 78 K and after warming to 123 K (blue line), and 153 K (green line). The red plot is assigned to **4**, which shows a distinct absorption at λ_{max} = 395 nm.

5. Computational Details

5.1 Computational Methods

The optimizations of molecular geometries and Hessian calculations were carried out with the ORCA 5.0.4[7] program under gas phase conditions. The r^{2}SCAN-3c[8] composite density functional method including the quasi-relativistic 60-electron pseudopotential (ECP60MWB)[9] for platinum was employed together with the resolution-of-the-identity (RI) algorithm[10] for fitting of Coulomb integrals. Zero-point vibrational energies (ZPVEs), thermal and entropic corrections were obtained at standard conditions (1 atm, 298.15 K). In addition, single point energy calculations based on the r^{2}SCAN-3c optimized molecular structures were performed with the mPW2PLYP double-hybrid density functional[11] together with the def2-QZVPP[12] basis set and the D4 empirical dispersion correction.[13] In these calculations, the RIJCOSX algorithm[10,14] was used together with the def2/J[15] and def2-QZVPP/C[16] auxiliary basis sets as implemented in ORCA.

For comparison with experimental vibrational data, molecular structures and unscaled harmonic vibrational frequencies were recomputed at the BP86/def2-TZVP[17] level of DFT utilizing the conductor-like polarizable continuum (CPCM)[18] solvent model with Tetrahydrofuran as solvent. Natural bond orbital (NBO) analyses were performed with the B3LYP(V)[19] hybrid density functional together with the def2-QZVPP basis set and the D4 empirical dispersion correction. For NBO analyses the NBO 7.0 program[20] was interfaced to ORCA.

Natural orbitals for chemical valence (NOCV)[21] analysis were performed at the r^{2}SCAN-3c optimized geometries with Multiwfn 3.8.[22] Wavefunctions of molecular systems and fragments were optimized within the zeroth-order regular approximation (ZORA)[23] utilizing the ZORA-def2-TZVP[24] basis set as provided by the ORCA basis set library for all non-metal atoms and the segmented all-electron relativistically contracted SARC-ZORA-TZVPP[24] basis set for Pt. The BP86 density functional was employed together with Grimme's D3(BJ) empirical dispersion correction with Becke-Johnson damping.[25]

UV/Vis spectra were computed based on the r^{2}SCAN-3c optimized structures employing time-dependent DFT (TD-DFT) using the B3LYP hybrid density functional together with the def2-TZVP basis set and the D4 empirical dispersion correction. Solvation effects (tetrahydrofuran) were treated by the CPCM solvent model. Simulated UV/VIS spectra were obtained with the *orca_mapspc* utility assuming a homogeneous line broadening of 3000 cm^{-1}.

Improved relative energies were obtained in single point calculations by a two-layer ONIOM(QM:QM) approach,[26] in which explicitly correlated coupled-cluster energies for H-

truncated model systems (‘high-level’, HL) are combined with r^2SCAN-3c energies for the real systems (‘low-level’, LL). The low-level method will be denoted ‘DFT’ in the following. The model systems were constructed by removing all *t*Bu and methyl groups of the r^2SCAN-3c optimized structures and adding hydrogen atoms along the original P–C bonds of the real systems. The resulting P–H bond lengths were relaxed at the r^2SCAN-3c level of theory with fixed bond angles and dihedral angles and frozen coordinates of all other atoms of the model system. Total energies at the ONIOM level were calculated by the following equation:

$$E_{ONIOM(QM:QM)} = E_{LL}^{real} - E_{LL}^{model} + E_{HL}^{model}$$

The high-level calculations were performed with the Molpro 2024 program[27] employing the CCSD(T*)-F12b method.[28] The perturbative triples contributions, denoted (T*), are extrapolated towards the complete basis set limit according to the scale factor E_{corr}(MP2-F12)/E_{corr}(MP2).[29] The cc-pV{D,T}Z-F12[30] basis sets for all non-metal atoms and the aug-cc-pV{D,T}Z-PP[31] basis set for platinum were used in combination with the corresponding triple-ζ quality JKfit auxiliary basis sets, the MP2fit sets for density fitting and the OptRI/JKfit sets for constructing the complementary auxiliary basis set (OptRI for non-metal atoms, JKfit for Pt).[32] All basis sets were used as provided in the Molpro basis set library.

Further, single-point calculations on the real system were performed with the DLPNO-CCSD(T) method[33] with T1 iterative triples as implemented in ORCA. In these calculations the correlation-consistent triple- and quadruple-zeta valence basis sets cc-pV{T,Q}Z[34] for all non-metal atoms and the cc-pV{T,Q}Z-PP basis sets for platinum were utilized along with the corresponding cc-pV{T,Q}Z/C and cc-pV{T,Q}Z-PP/C auxiliary basis sets.[35] All correlation-consistent basis sets used for platinum include a 60-electron relativistic pseudopotential (SK-MCDHF-RSC).[36] *VeryTightSCF* convergence criteria, *TightPNO* and default frozen-core settings were used for all DLPNO-CCSD(T) calculations. T_{cutPNO} thresholds of 10^{-6} and 10^{-7} were used and correlation energies were extrapolated towards the complete pair natural orbital (PNO) space as proposed by Altun *et al.*:[37]

$$E_{corr}^{CPS} = E_{corr}^{6} + 1.5 \cdot (E_{corr}^{7} - E_{corr}^{6})$$

E_{corr}^{CPS} denotes the extrapolated correlation energy at the complete PNO space (CPS) limit and E_{corr}^{X} denotes the correlation energy obtained with the $T_{\mathrm{cutPNO}} = 10^{-x}$ value. The reference and the CPS-extrapolated correlation energies were extrapolated towards the complete basis set

(CBS) limit by separate two-point extrapolation schemes. The HF reference energies were extrapolated to the CBS limit E_{ref}^{CBS} employing the exponential ansatz proposed by Klopper and Kutzelnigg:[38]

$$E_{\mathrm{ref}}^{\mathrm{CBS}} = E_{\mathrm{ref}}^{\mathrm{Y}} + \frac{E_{\mathrm{ref}}^{\mathrm{Y}} - E_{\mathrm{ref}}^{\mathrm{X}}}{\exp\left(\alpha\sqrt{\mathrm{Y}} - \alpha\sqrt{\mathrm{X}}\right) - 1}$$

The correlation energies were extrapolated to the CBS limit E_{corr}^{CBS} employing Truhlar's ansatz:[39]

$$E_{corr}^{CBS} = \frac{X^{\beta} E_{corr}^{X} - Y^{\beta} E_{corr}^{Y}}{X^{\beta} - Y^{\beta}}$$

X = 3 and Y = 4 denote the cardinal numbers used for the extrapolation. The parameters α = 5.46 and β = 3.05 were chosen as proposed by Neese.[40]

5.2 Molecular Structures of 1, 2, 3, 4 and 5

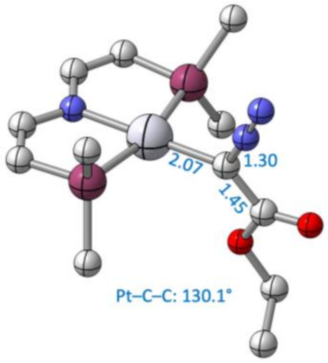

(PNP)Pt{C(N_2)CO_2Et} (**1**)

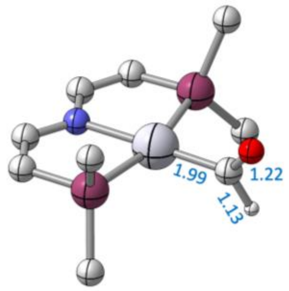

(PNP)Pt(CHO) (**2**)

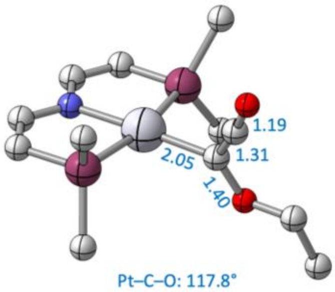

(PNP)Pt{C(CO)OEt} (**3**)

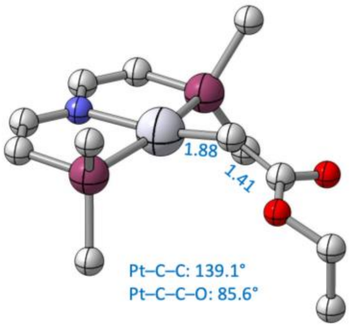

^{S}(PNP)Pt{CCO_2Et} (^{S}**4**)

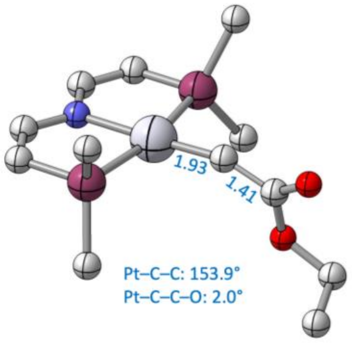

^{T}(PNP)Pt{CCO_2Et} (^{T}**4**)

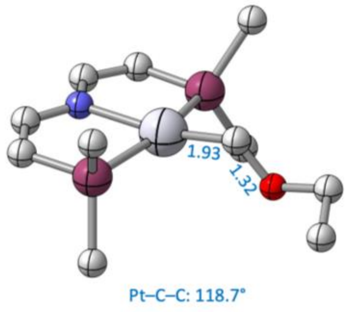

^{S}(PNP)Pt{COEt} (^{S}**5**)

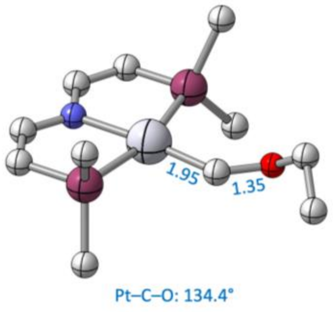

^{T}(PNP)Pt{COEt} (^{T}**5**)

Figure S33. DFT-optimized molecular structures (r^{2}SCAN-3c) of the complexes **1**, **2**, **3**, ^{S}**4**, ^{T}**4**, ^{S}**5** and ^{T}**5**. H atoms and methyl groups not shown for clarity.

The molecular structures of singlet ^{S}**4** and triplet ^{T}**4** differ by a rotation of the carboxyl group. In ^{S}**4** the carbene and the carboxyl group exhibit an orthogonal orientation ($\omega_{Pt–C–C–O}$ = 85.6°) in contrast to the coplanar arrangement in ^{T}**4** ($\omega_{Pt–C–C–O}$ = 2.0°). The broken-symmetry singlet transition-state for the rotation of the carboxyl group was optimized (Figure 34) and the

computed rotational barrier of $\Delta G^{\ddagger}$ = 12.0 kcal mol^{-1} indicates that $^{\mathbf{S}}$**4** resides in a rather steep potential.

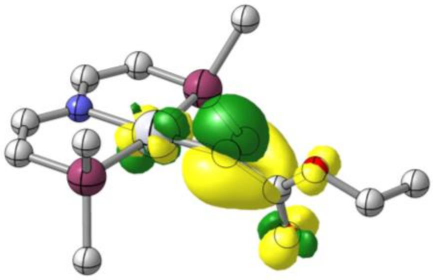

^{BS}**TS3**
$\langle S^2 \rangle = 1.00$
i61 cm^{-1}

Figure S34. Spin density of the DFT-optimized molecular structure of the transition state for the carboxyl group rotation on the broken-symmetry singlet potential surface ($\Delta G^{\ddagger}$ = 12.0 kcal mol^{-1}, mPW2PLYP-D4/def2-QZVPP//r^{2}SCAN-3c).

5.3 Calculated IR Spectra of 1, 2 and 3

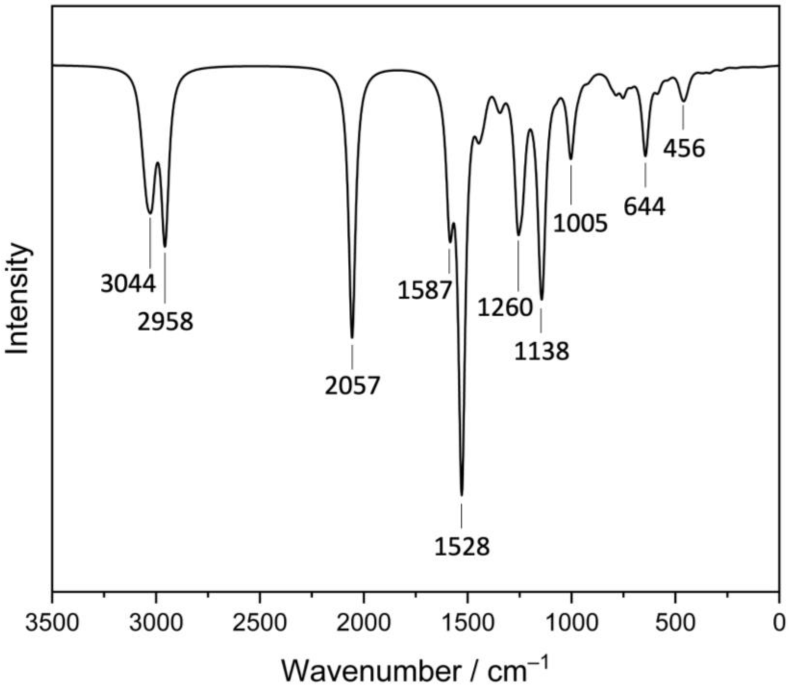

Figure S35. Computed IR spectrum of **1** (CPCM(THF)-BP86/def2-TZVP, Lorentzian broadening with 20 cm^{-1} FWHM).

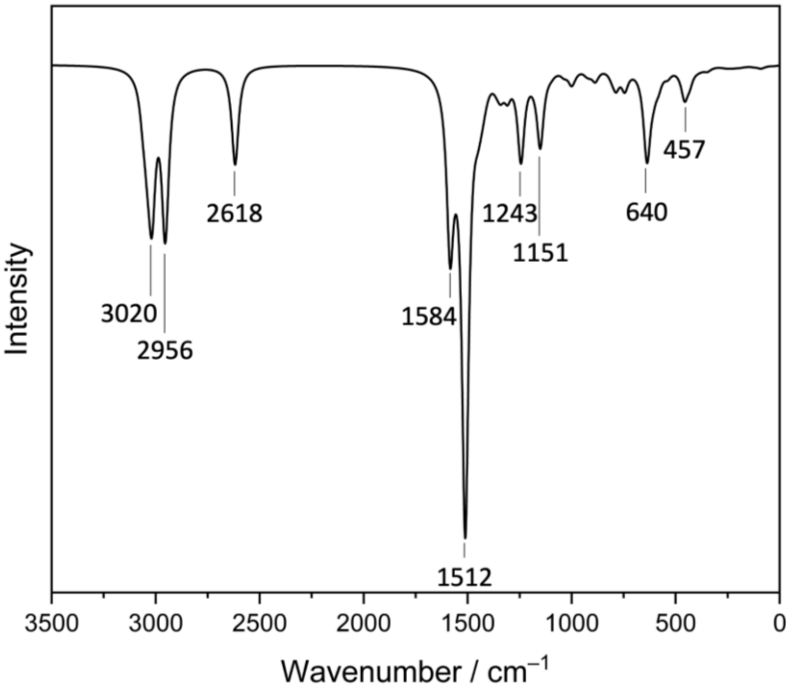

Figure S36. Computed IR spectrum of **2** (CPCM(THF)-BP86/def2-TZVP, Lorentzian broadening with 20 cm^{-1} FWHM).

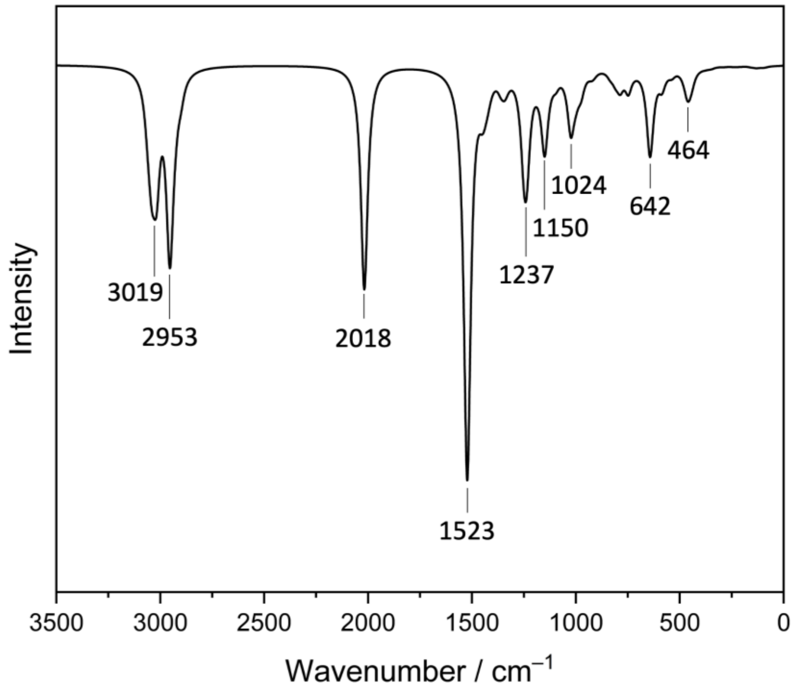

Figure S37. Computed IR spectrum of **3** (CPCM(THF)-BP86/def2-TZVP, Lorentzian broadening with 20 cm^{-1} FWHM).

5.4 Calculated UV/VIS Spectra of 1, 2, 3, 4 and 5

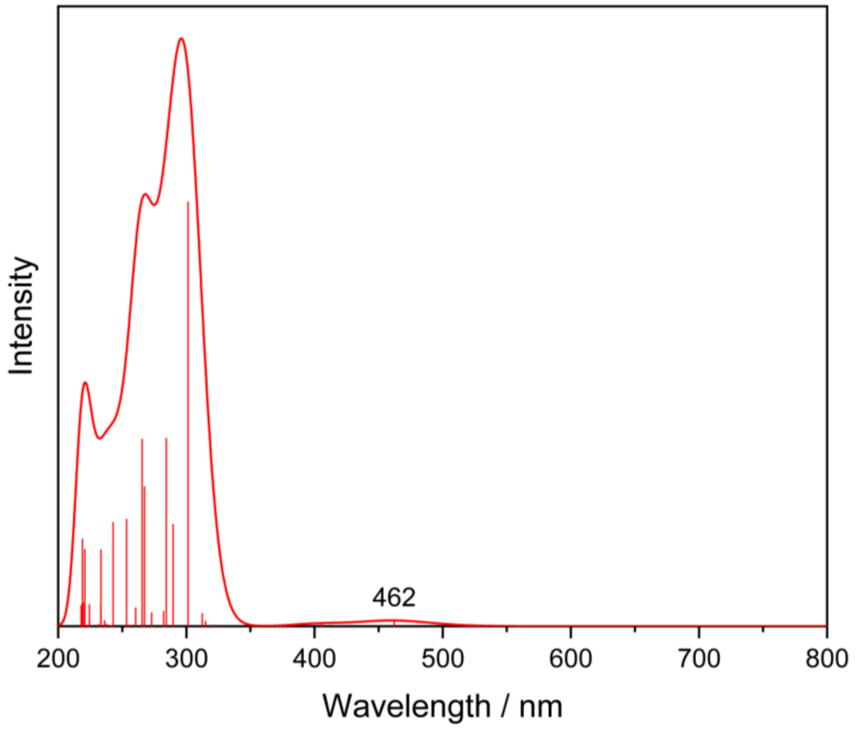

Figure S38. Computed UV/Vis spectrum of **1** (CPCM(THF)-B3LYP-D4/def2-TZVP//r^2SCAN-3c).

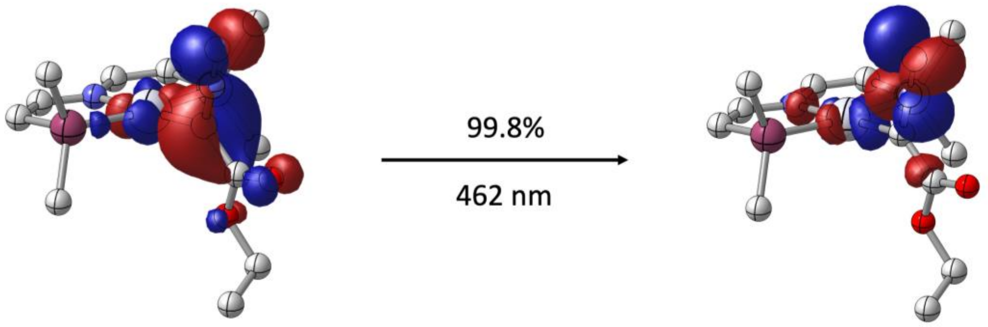

Figure S39. Natural transition orbitals representing (99.8%) the absorption band of **1** at 462 nm (isosurfaces at ± 0.05 $a_0^{-3/2}$, hydrogen atoms and methyl groups not shown for clarity).

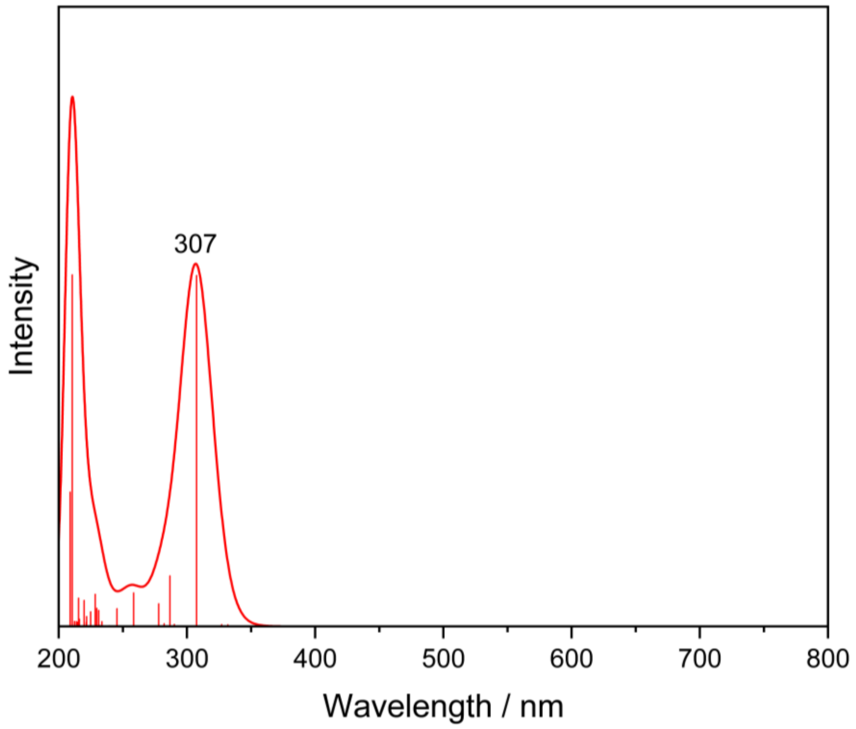

Figure S40. Computed UV/Vis spectrum of **2** (CPCM(THF)-B3LYP-D4/def2-TZVP//r^{2}SCAN-3c).

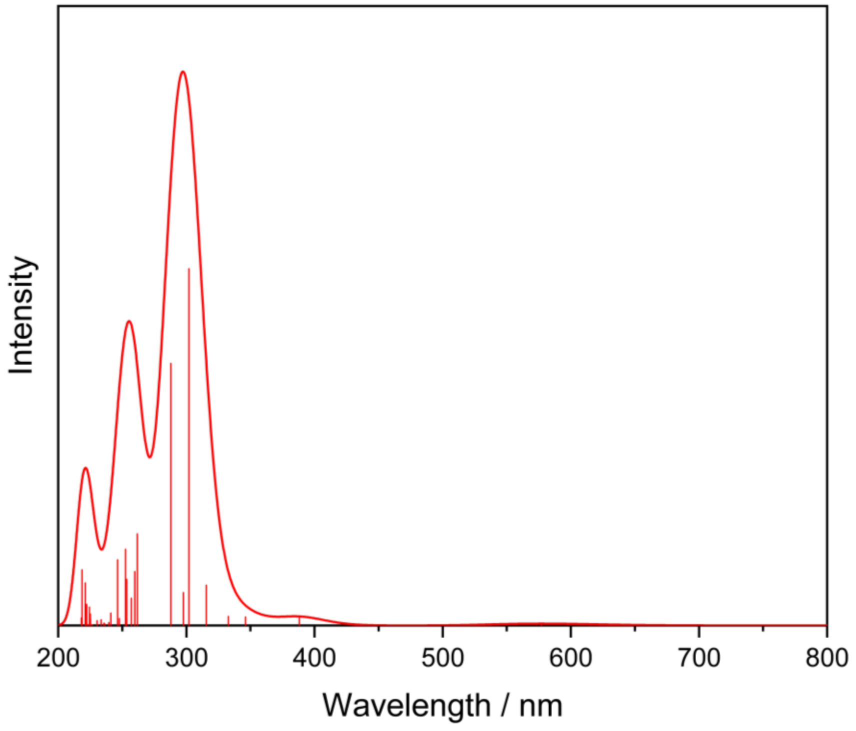

Figure S41. Computed UV/Vis spectrum of **3** (CPCM(THF)-B3LYP-D4/def2-TZVP//r^{2}SCAN-3c).

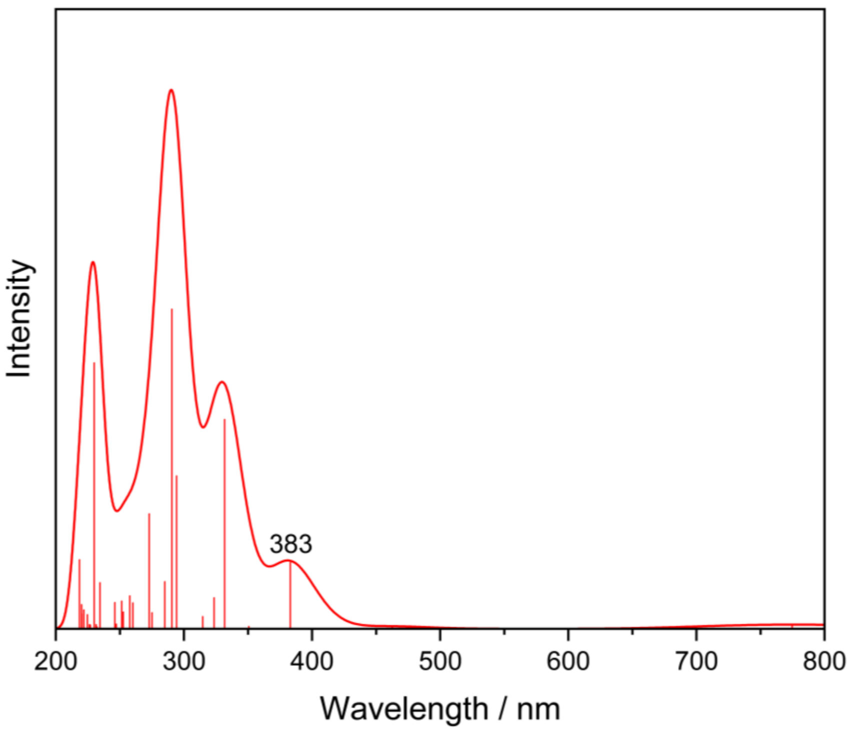

Figure S42. Computed UV/Vis spectrum of ^{S}**4** (CPCM(THF)-B3LYP-D4/def2-TZVP//r^{2}SCAN-3c).

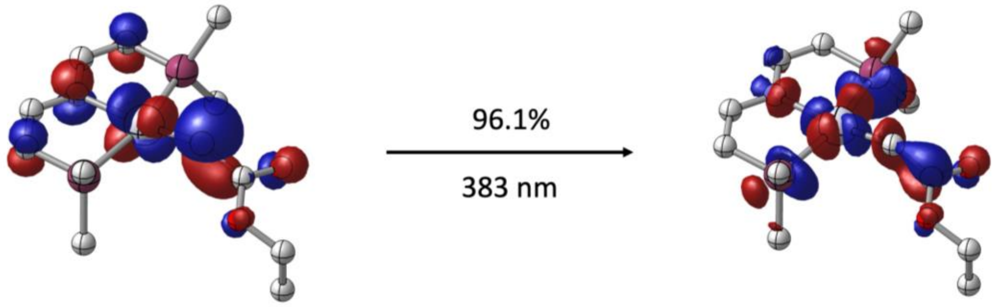

Figure S43. Natural transition orbitals representing (96.1%) the absorption band of ^{S}**4** at 383 nm (isosurfaces at ± 0.05 $a_0^{-3/2}$, hydrogen atoms and methyl groups not shown for clarity).

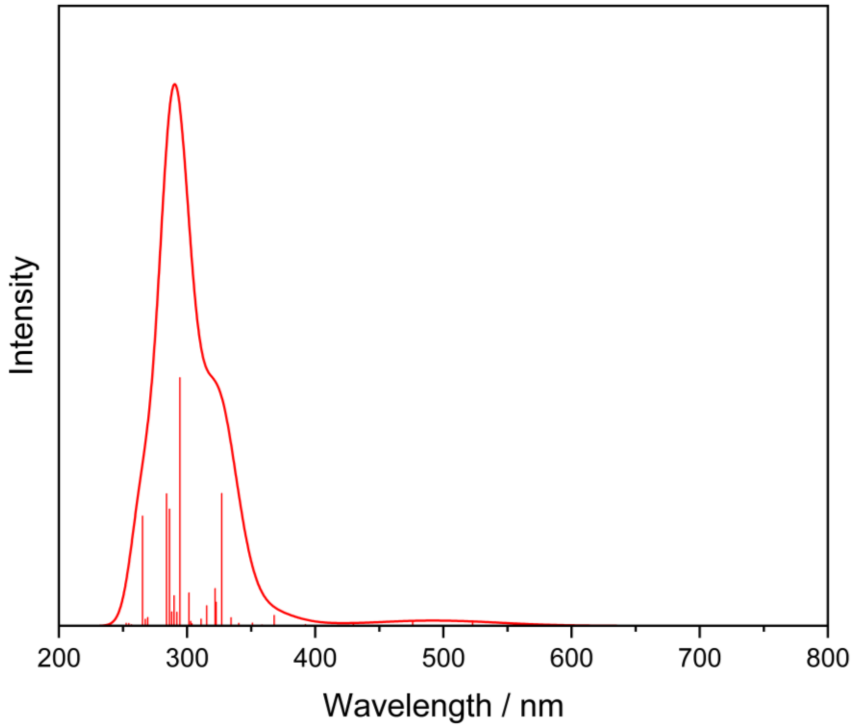

Figure S44. Computed UV/Vis spectrum of $^{T}\mathbf{4}$ (CPCM(THF)-B3LYP-D4/def2-TZVP//r^{2}SCAN-3c).

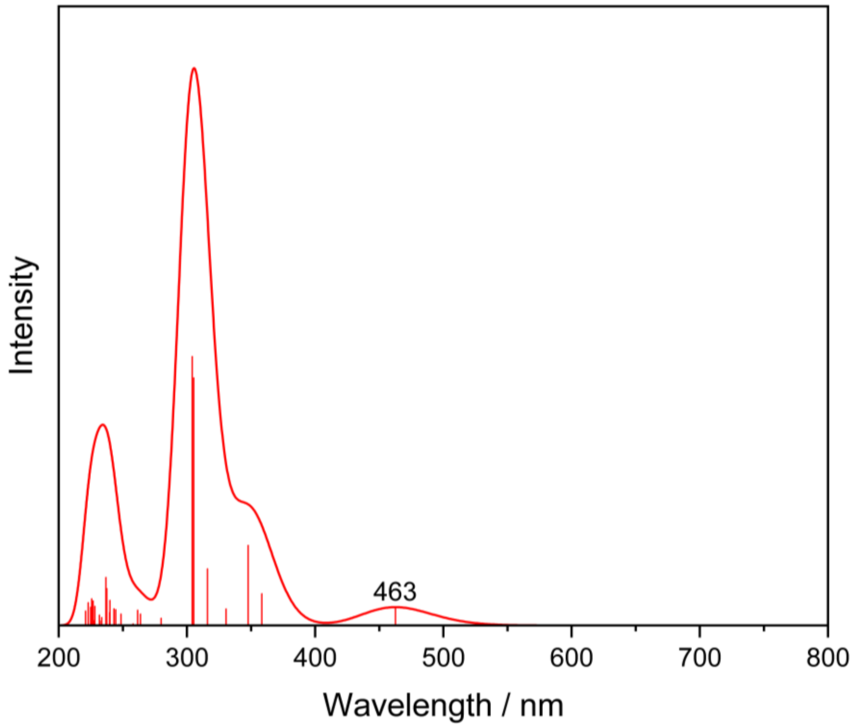

Figure S45. Computed UV/Vis spectrum of $^{S}\mathbf{5}$ (CPCM(THF)-B3LYP-D4/def2-TZVP//r^{2}SCAN-3c).

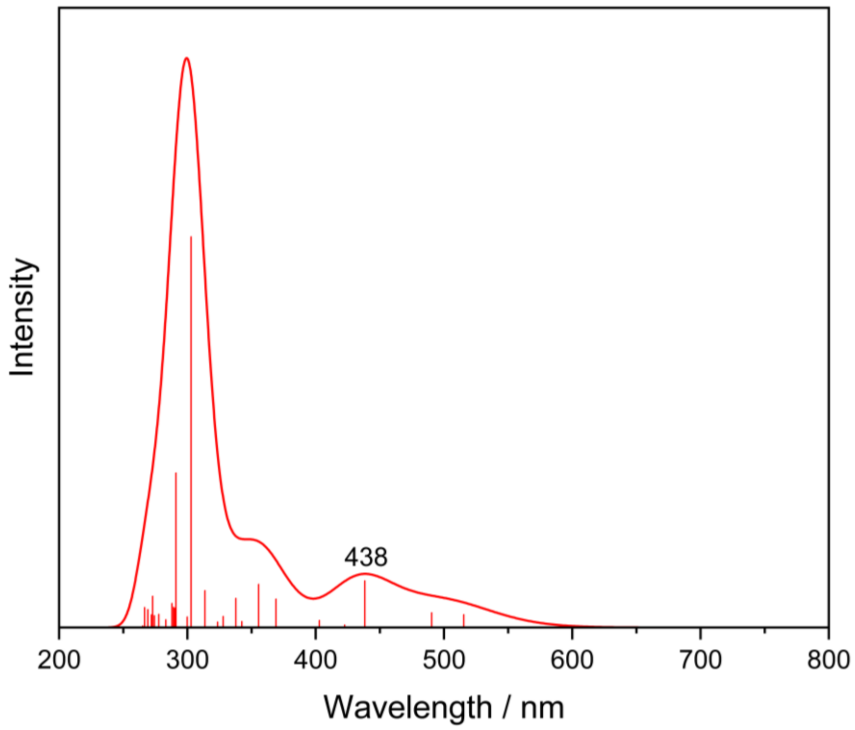

Figure S46. Computed UV/Vis spectrum of **^{T}5** (CPCM(THF)-B3LYP-D4/def2-TZVP//r^{2}SCAN-3c).

5.5 Singlet-Triplet Energy Differences for 4 and 5

Relative spin-state energies of the carbenes **4** and **5** were computed with DFT and coupled cluster methods (Table S16).

$$\Delta G_{ST} = G_{tot}(T) - G_{tot}(S)$$

All computational methods predict singlet ground states for both carbenes. ONIOM(CC:DFT) results converge at ΔG_{ST}(**4**) = 5.9 kcal mol^{-1} and ΔG_{ST}(**5**) = 23.7 kcal mol^{-1}. Extrapolated DLPNO-CCSD(T) results place the triplet electromers slightly higher with ΔG_{ST}(**4**) = 6.5 kcal mol^{-1} and ΔG_{ST}(**5**) = 25.2 kcal mol^{-1}. The results of the double hybrid functional mPW2PLYP are in good agreement with the ONIOM results. The compound DFT method r^{2}SCAN-3c overstabilizes the triplet electromer in both cases by about 5-6 kcal mol^{-1} compared to the ONIOM results.

Table S16. Singlet-triplet free energy differences (ΔG_{ST}) in kcal mol^{-1} for **4** and **5**. Positive ΔG_{ST} values indicate a singlet ground state.

Method	ΔG_{ST}(**4**)	ΔG_{ST}(**5**)
r^{2}SCAN-3c	0.5	18.2
mPW2PLYP-D4/def2-QZVPP	5.8	23.3
DLPNO-CCSD(T)/CBS[T/Q]/CPS[6/7]	6.5	25.2
ONIOM(CCSD(T*)-F12/VDZ:r^{2}SCAN-3c)	5.9	24.0
ONIOM(CCSD(T*)-F12/VTZ:r^{2}SCAN-3c)	5.9	23.7

5.6 Characterization of Metallocarbene 4

The NLMO analysis of **4** reveals the orbital interactions between the d-orbitals of Pt, the p-orbitals of $C_{Carbene}$ and the p-orbitals of C_{CO_2R}. In the singlet state, the $p_z(C_{Carbene})$ orbital is doubly occupied and the orthogonal $p_y(C_{Carbene})$ orbital is empty. The NLMO representing the lone pair of $C_{Carbene}$ has a 9% contribution of the $p_z(C_{CO_2R})$ orbital, indicating a pull interaction between $C_{Carbene}$ and C_{CO_2R}. The push interaction of the $d_{xy}(Pt)$ orbital into the empty $p_y(C_{Carbene})$ is indicated by a 11% contribution of $p_y(C_{Carbene})$ at the NLMO representing the $d_{xy}(Pt)$ orbital.

In the triplet state, both the $p_y(C_{Carbene})$ and the $p_z(C_{Carbene})$ orbitals are singly occupied. Due to the pull interaction of the ester substituent, the NLMO representing the $p_y(C_{Carbene})$ orbital has a significant contribution (32%) of the neighbouring $p_y(C_{CO_2R})$. In contrast, the NLMO representing the $p_z(C_{Carbene})$ orbital is primarily localized on the $C_{Carbene}$, indicating a preferential orientation of the pull interaction of the ester substituent. The metal substituent shows no preferential orientation for the push interaction, as the NLMOs representing the $d_{xy}(Pt)$ and $d_{xz}(Pt)$ orbitals both show comparable contributions of the corresponding $C_{Carbene}$ orbitals.

The push/pull interactions of the substituents with the carbene center can be assessed by NOCV analysis, in which the deformation density of two fragments A and B of the bound complex A–B is analyzed. The NOCVs decompose the deformation density into diagonal contributions, resulting in pairwise NOCV orbital interactions, that are associated with an energy contribution and the amount of charge flow of each NOCV pair. The deformation density, that is predominantly localized in the bond region between the two fragments, is typically represented by only a small number of significant NOCV pairs describing the bond formation between the two fragments. The choice of fragments and their electronic reference state is a crucial part of the NOCV analysis, because this directly affects the results of the analysis. The orbital reorganization is described by ΔE_{orb}, so the fragmentation scheme with the lowest absolute value for ΔE_{orb} most closely resembles the electronic situation in the bound complex (Frenking-Parr criterion).[41]

The Pt–C bond of **4** can be cleaved homolytically or heterolytically. Based on the Frenking-Parr criterion, the heterolytic cleavage into a $^{S}[(PNP)Pt]^{+}$ and a $^{S}[C\text{–}COOEt]^{-}$ fragment for the singlet carbene and into a $^{S}[(PNP)Pt]^{+}$ and a $^{T}[C\text{–}COOEt]^{-}$ fragment for the triplet carbene is favoured over homolytic fragmentation. Besides the formation of a dative σ-bond, the NOCV analysis reveals significant π-type Pt⟶C charge flow. In the singlet carbene, the π-type charge flow of 0.67 electrons is located in the xy-plane of the molecule. The π-interactions in the triplet carbene are described by two β-NOCV pairs describing a charge flow of 0.27 β-

electrons in the xy-plane and 0.19 electrons in the xz-plane associated with energetic contributions of –9.7 kcal mol^{-1} and –7.5 kcal mol^{-1}.

The best fragmentation schemes for the $C_{Carbene}$– C_{CO_2R} bond of **4** are a heterolytic fragmentation into ^{S}[(PNP)PtC]$^{-}$ and ^{S}[COOEt]$^{+}$ for the singlet carbene and a homolytic fragmentation into ^{Q}[(PNP)PtC] and ^{D}[COOEt] for the triplet carbene. In the singlet carbene, the NOCV analysis reveals a dominant π-type $C_{Carbene}$⟶C_{CO_2R} charge flow of 0.83 electrons and a smaller orthogonal π-type C_{CO_2R} ⟶$C_{Carbene}$ charge flow of 0.43 electrons. In the optimized structure of ^{S}**4**, the $C_{Carbene}$ atom and the carboxylate group are tilted out of the Pt(PNP)-plane (cf. S33); thus, the NOCV charge flow associated with the $C_{Carbene}$/C_{CO_2R} pull interaction exhibits a distortion from a strictly π-symmetric appearance ($\Delta\rho(\pi_z)$: $p_z(C_{Carbene})$⟶$\pi_z^*(C_{CO_2R})$; Figure S50 below and Figure 5d in the main text). For the triplet carbene, the NOCV analysis reveals two NOCV pairs representing orthogonal π-type $C_{Carbene}$⟶C_{CO_2R} charge flows of 0.41 and 0.16 electrons. The difference in the amount of charge flow in those two NOCV pairs highlights the preferential orientation of the pull interaction of the ester substituent.

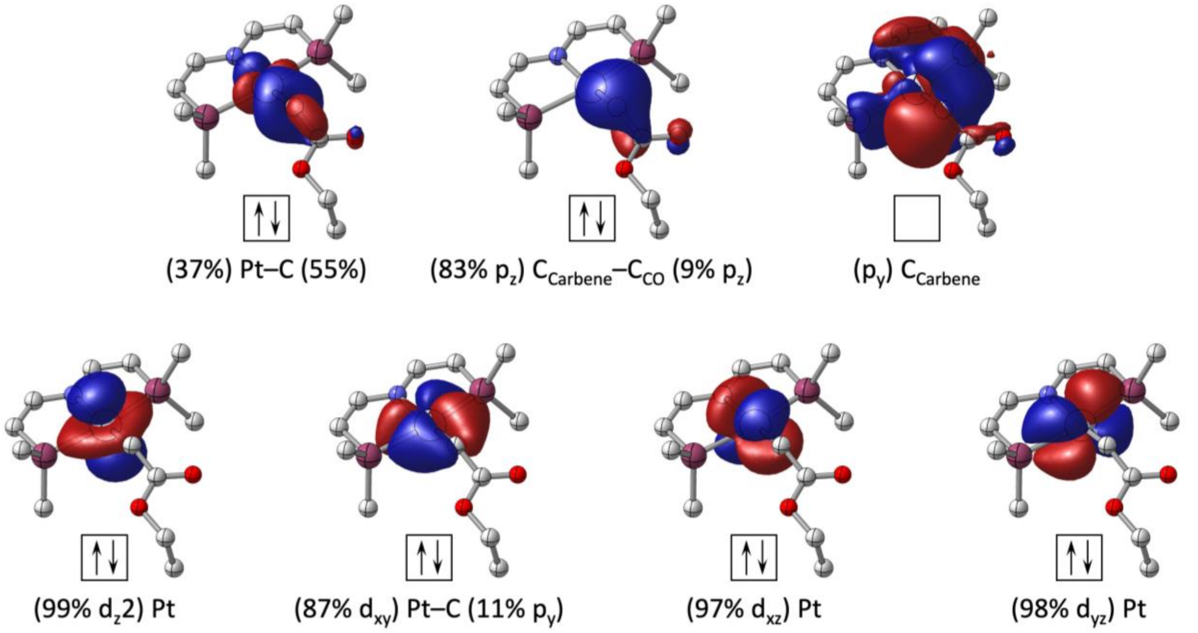

Figure S47. Characteristic NLMOs resulting from an NBO analysis of the singlet ground state of **4** (B3LYP-D4/def2-QZVPP//r^{2}SCAN-3c; isovalue ±0.05 $a_0^{-3/2}$, hydrogen atoms and ^{t}Bu methyl groups not shown).

Table S17. Fragmentation patterns for the fragmentation of the Pt–C bond of ^{S}**4** for NOCV analysis with their respective orbital interaction energies ΔE_{orb} (ZORA-BP86-D3BJ/ZORA-def2-TZVP//r^{2}SCAN-3c). All energies in kcal mol^{-1}.

Fragments	^{S}[(PNP)Pt]$^{+}$ ^{S}[C–COOEt]$^{-}$	^{D}[(PNP)Pt] ^{D}[C–COOEt]
ΔE_{orb}	–137.6	–228.4

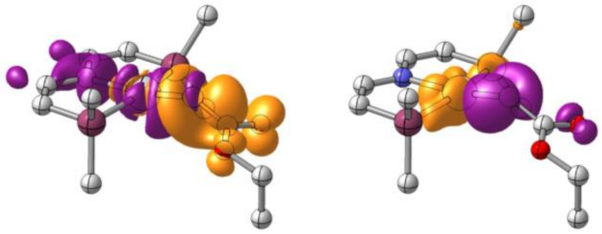

Δρ(σ) |ν| = 0.90 –70.1 kcal mol^{-1}

Δρ(π$_y$) |ν| = 0.67 –28.2 kcal mol^{-1}

Figure S48. Selected NOCV deformation densities associated with the σ and π interactions of the fragments ^{S}[(PNP)Pt]$^{+}$ and ^{S}[C–COOEt]$^{-}$ of ^{S}**4** (ZORA-BP86-D3BJ/ZORA-def2-TZVP//r^{2}SCAN-3c, eigenvalues |ν|: electron density transferred, charge flow: orange⟶purple, isovalue ±0.002 a_0^{-3}, hydrogen atoms and *^{t}*Bu methyl groups not shown).

Table S18. Fragmentation patterns for the fragmentation of the $C_{Carbene}$–C_{CO_2R} bond of ^{S}**4** for NOCV analysis with their respective orbital interaction energies ΔE_{orb} (ZORA-BP86-D3BJ/ZORA-def2-TZVP//r^{2}SCAN-3c). All energies in kcal mol^{-1}.

Fragments	^{S}[(PNP)PtC]$^{+}$ ^{S}[COOEt]$^{-}$	^{D}[(PNP)PtC] ^{D}[COOEt]	^{S}[(PNP)PtC]$^{-}$ ^{S}[COOEt]$^{+}$
ΔE_{orb}	–596.1	–397.3	–385.5

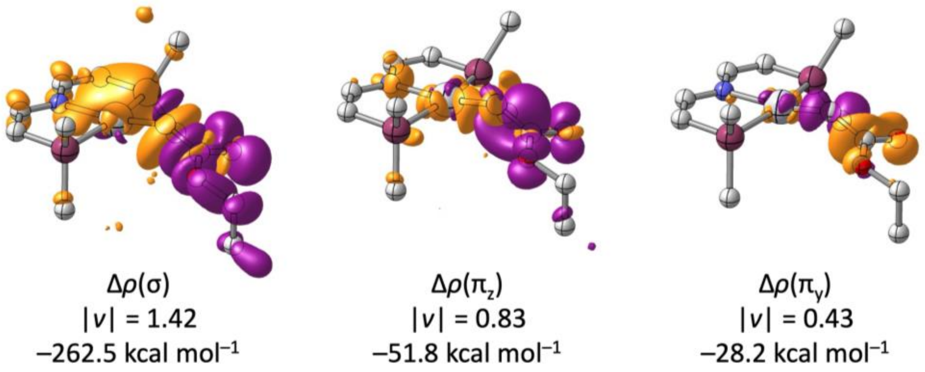

Figure S49. Selected NOCV deformation densities associated with the σ and π interactions of the fragments ^{S}[(PNP)PtC]$^{–}$ and ^{S}[COOEt]$^{+}$ of ^{S}**4** (ZORA-BP86-D3BJ/ZORA-def2-TZVP//r^{2}SCAN-3c, eigenvalues |ν|: electron density transferred, charge flow: orange⟶purple, isovalue ±0.002 a_0^{-3}, hydrogen atoms and *t*Bu methyl groups not shown).

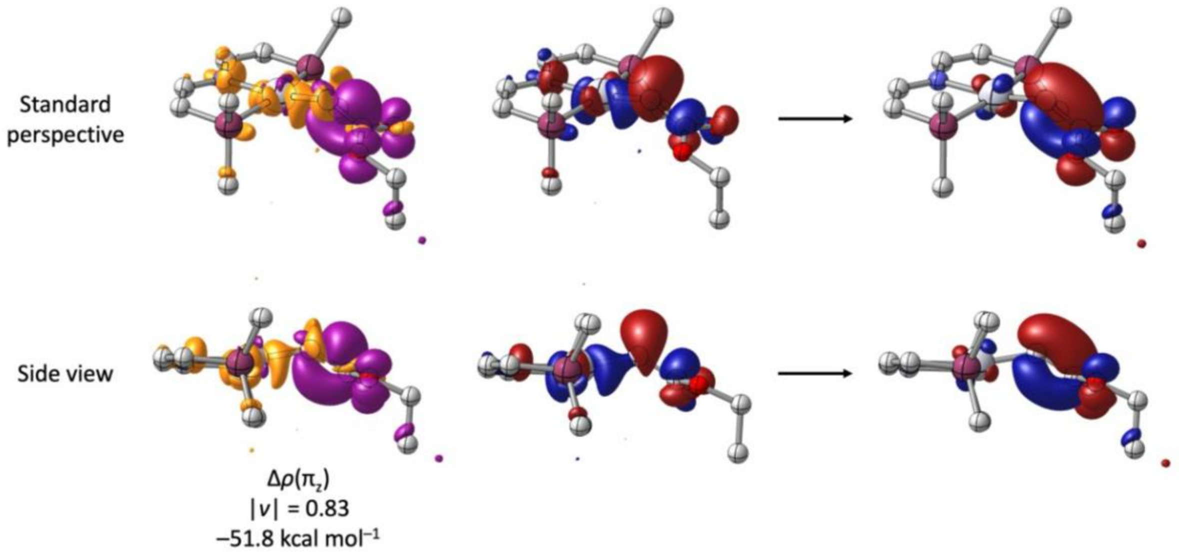

Figure S50. NOCV deformation density and the corresponding NOCV orbital pair associated with the π_z interaction of the fragments ^{S}[(PNP)PtC]$^{–}$ and ^{S}[COOEt]$^{+}$ of ^{S}**4** in the standard perspective and a side view (see text above, ZORA-BP86-D3BJ/ZORA-def2-TZVP//r^{2}SCAN-3c, eigenvalues |ν|: electron density transferred, charge flow: orange⟶purple, isovalues ±0.002 a_0^{-3} for NOCV deformation density and ±0.05 $a_0^{-3/2}$ for NOCV orbitals, hydrogen atoms and *t*Bu methyl groups not shown).

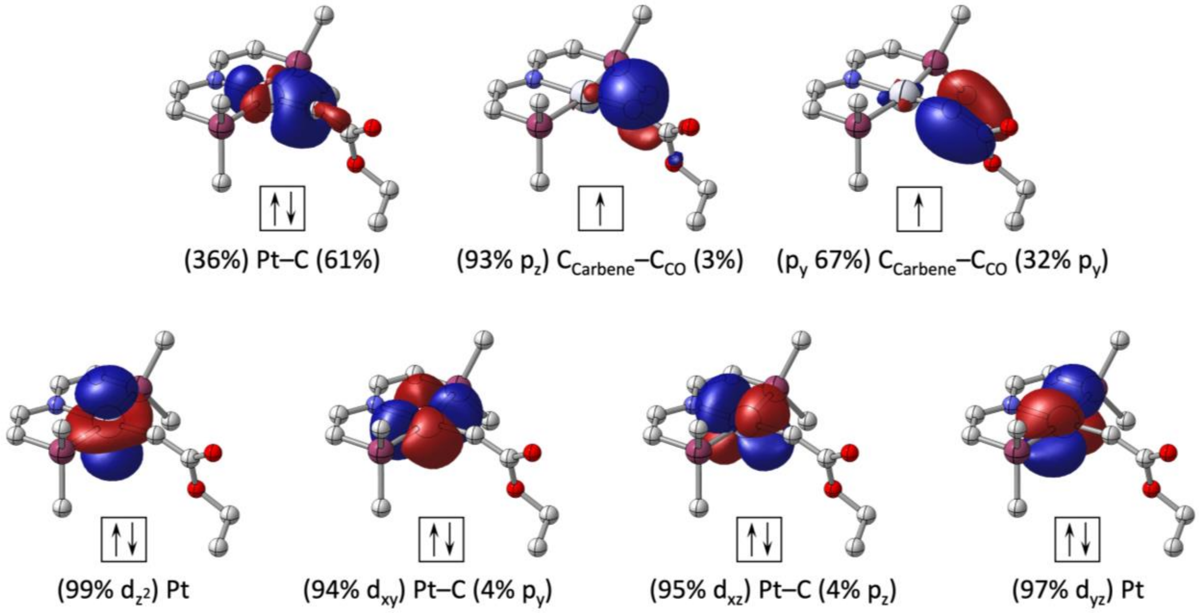

Figure S51. Characteristic NLMOs resulting from an NBO analysis of the triplet ground state of **4** (B3LYP-D4/def2-QZVPP//r^{2}SCAN-3c; isovalue ±0.05 $a_0^{-3/2}$, hydrogen atoms and ^{t}Bu methyl groups not shown). Results for doubly occupied orbitals were obtained by averaging over the respective α and β spin orbitals.

Table S19. Fragmentation patterns for the fragmentation of the Pt–C bond of ^{T}**4** for NOCV analysis with their respective orbital interaction energies ΔE_{orb} (ZORA-BP86-D3BJ/ZORA-def2-TZVP//r^{2}SCAN-3c). All energies in kcal mol^{-1}.

Fragments	^{S}[(PNP)Pt]$^{+}$<br>^{T}[C–COOEt]$^{-}$	^{D}[(PNP)Pt]<br>^{Q}[C–COOEt]
ΔE_{orb}	–120.1	–169.1

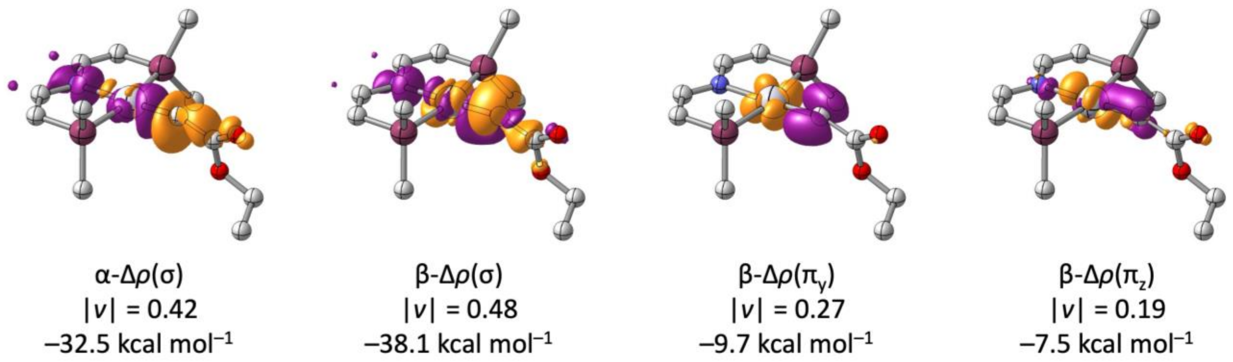

Figure S52. Selected NOCV deformation densities associated with the σ and π interactions of the fragments ^{S}[(PNP)Pt]$^{+}$ and ^{T}[C–COOEt]$^{–}$ of ^{T}**4** (ZORA-BP86-D3BJ/ZORA-def2-TZVP//r^{2}SCAN-3c, eigenvalues |ν|: electron density transferred, charge flow: orange⟶purple, isovalue ± 0.002 a_0^{-3}, hydrogen atoms and ^{t}Bu methyl groups not shown).

Table S20. Fragmentation patterns for the fragmentation of the $C_{Carbene}$–C_{CO_2R} bond of ^{T}**4** for NOCV analysis with their respective orbital interaction energies ΔE_{orb} (ZORA-BP86-D3BJ/ZORA-def2-TZVP//r^{2}SCAN-3c). All energies in kcal mol$^{–1}$.

Fragments	^{Q}[(PNP)PtC]<br>^{D}[COOEt]	^{T}[(PNP)PtC]$^{–}$<br>^{S}[COOEt]$^{+}$
ΔE_{orb}	–259.5	–373.7

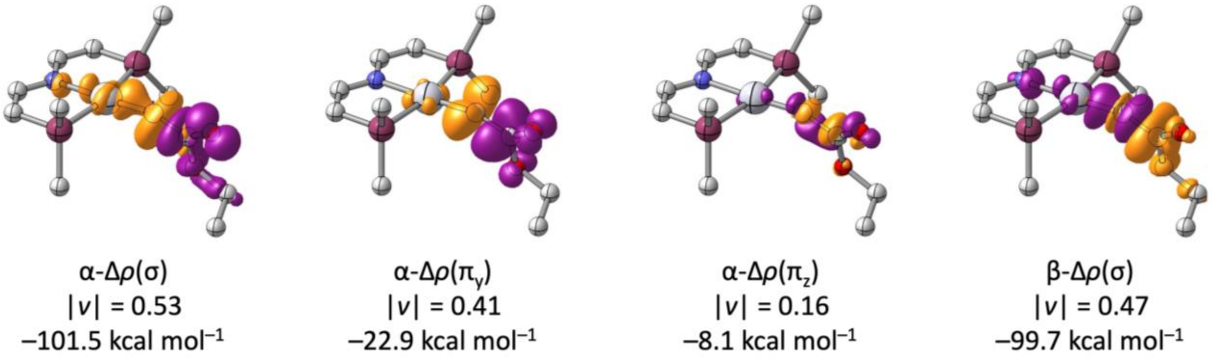

Figure S53. Selected NOCV deformation densities associated with the σ and π interactions of the fragments ^{Q}[(PNP)PtC] and ^{D}[COOEt] of ^{T}**4** (ZORA-BP86-D3BJ/ZORA-def2-TZVP//r^{2}SCAN-3c, eigenvalues |ν|: electron density transferred, charge flow: orange⟶purple, isovalue ± 0.002 a_0^{-3}, hydrogen atoms and ^{t}Bu methyl groups not shown).

Table S21. Homolytic bond dissociation energies of the Pt–C bonds of ^{S}**4** and ^{T}**4** according to $^{S/T}(PNP)PtCO_2Et \longrightarrow {}^{D}(PNP)Pt + {}^{D}CO_2Et$ (at 298 K, all energies in kcal mol^{-1}).

	^{S}**4**	^{T}**4**
$\Delta E_{\text{Pt–C}}$[a]	100.1	92.9
$\Delta H_{\text{contr}}^{298\text{ K}}$[b]	−2.6	−2.3
$\Delta H_{\text{Pt–C}}^{\text{BDE}}$[c]	97.6	90.7

[a] mPW2PLYP-D4/def2-QZVPP//r^{2}SCAN-3c.
[b] Thermal correction to enthalpy from vibrational analysis (r^{2}SCAN-3c).
[c] $\Delta H_{\text{Pt–C}}^{\text{BDE}} = \Delta E_{\text{Pt–C}} + \Delta H_{\text{contr}}^{298\text{ K}}$.

5.7 Characterization of Metallocarbene 5

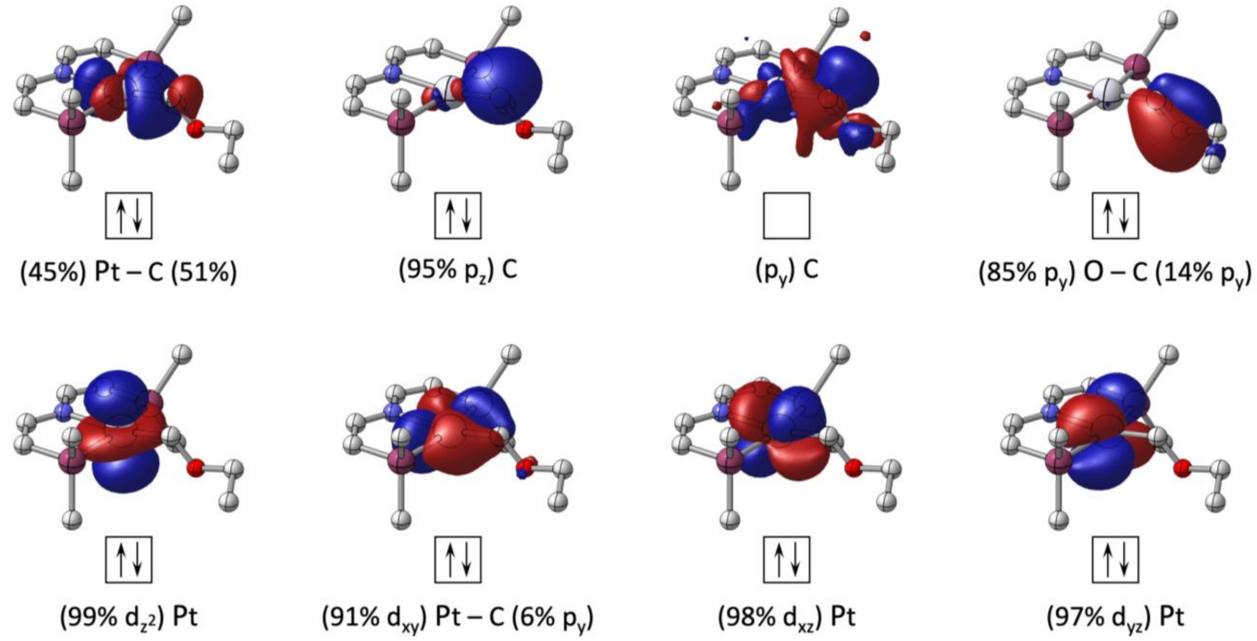

Figure S54. Characteristic NLMOs resulting from an NBO analysis of the singlet ground state of **5** (B3LYP-D4/def2-QZVPP//r^{2}SCAN-3c; isovalue ±0.05 $a_0^{-3/2}$, hydrogen atoms and ^{t}Bu methyl groups not shown).

Table S22. Fragmentation patterns for the fragmentation of the Pt–C bond of ^{S}**5** for NOCV analysis with their respective orbital interaction energies ΔE_{orb} (ZORA-BP86-D3BJ/ZORA-def2-TZVP//r^{2}SCAN-3c). All energies in kcal mol^{-1}.

Fragments	^{S}[(PNP)Pt]$^+$ ^{S}[C–OEt]$^-$	^{D}[(PNP)Pt] ^{D}[C–OEt]	^{S}[(PNP)Pt]$^-$ ^{S}[C–OEt]$^+$
ΔE_{orb}	–152.8	–231.9	–402.6

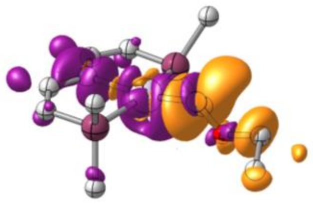

Figure S55. Selected NOCV deformation densities associated with the σ and π interactions of the fragments ^{S}[(PNP)Pt]$^{+}$ and ^{S}[C–OEt]$^{–}$ of ^{S}**5** (ZORA-BP86-D3BJ/ZORA-def2-TZVP//r^{2}SCAN-3c, eigenvalues |ν|: electron density transferred, charge flow: orange⟶purple, isovalue ±0.002 a_0^{-3}, hydrogen atoms and ^{t}Bu methyl groups not shown).

Table S23. Fragmentation patterns for the fragmentation of the C–O bond of ^{S}**5** for NOCV analysis with their respective orbital interaction energies ΔE_{orb} (ZORA-BP86-D3BJ/ZORA-def2-TZVP//r^{2}SCAN-3c). All energies in kcal mol^{-1}.

Fragments	^{S}[(PNP)PtC]$^{+}$ ^{S}[OEt]$^{–}$	^{D}[(PNP)PtC] ^{D}[OEt]	^{S}[(PNP)PtC]$^{–}$ ^{S}[OEt]$^{+}$
ΔE_{orb}	–376.7	–500.6	–701.8

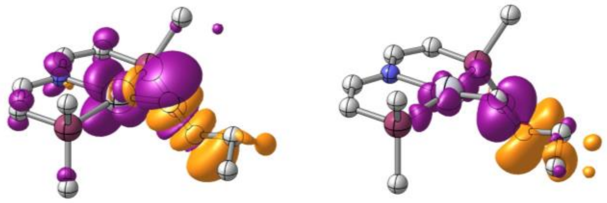

Figure S56. Selected NOCV deformation densities associated with the σ and π interactions of the fragments ^{S}[(PNP)PtC]$^{+}$ and ^{S}[OEt]$^{–}$ of ^{S}**5** (ZORA-BP86-D3BJ/ZORA-def2-TZVP//r^{2}SCAN-3c, eigenvalues |ν|: electron density transferred, charge flow: orange⟶purple, isovalue ±0.002 a_0^{-3}, hydrogen atoms and ^{t}Bu methyl groups not shown).

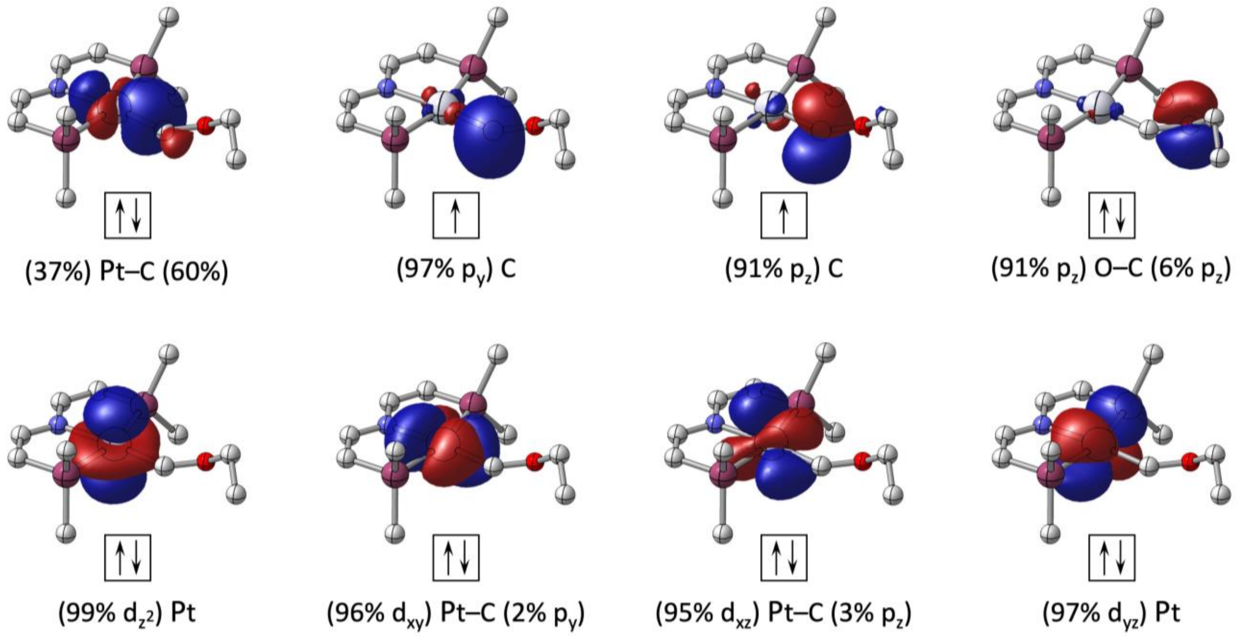

Figure S57. Characteristic NLMOs resulting from an NBO analysis of the triplet ground state of **5** (B3LYP-D4/def2-QZVPP//r^{2}SCAN-3c; isovalue ±0.05 $a_0^{-3/2}$, hydrogen atoms and ^{t}Bu methyl groups not shown). Results for doubly occupied orbitals were obtained by averaging over the respective α and β spin orbitals.

Table S24. Fragmentation patterns for the fragmentation of the Pt–C bond of $^{\mathbf{T}}$**5** for NOCV analysis with their respective orbital interaction energies ΔE_{orb} (ZORA-BP86-D3BJ/ZORA-def2-TZVP//r^{2}SCAN-3c). All energies in kcal mol^{-1}.

Fragments	^{S}[(PNP)Pt]$^+$<br>^{T}[C–OEt]$^-$	^{D}[(PNP)Pt]<br>^{Q}[C–OEt]
ΔE_{orb}	–126.0	–168.6

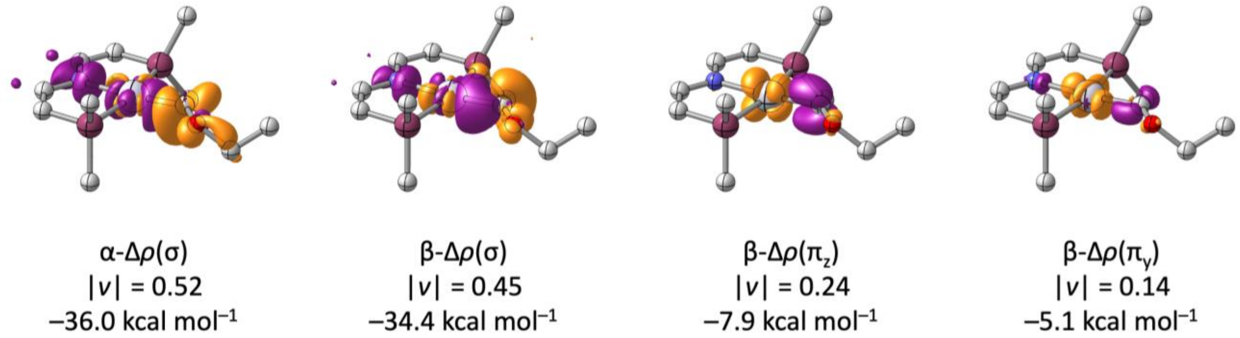

Figure S58. Selected NOCV deformation densities associated with the σ and π interactions of the fragments ^{S}[(PNP)Pt]$^{+}$ and ^{T}[C–OEt]$^{–}$ of $^{\mathbf{T}}$**5** (ZORA-BP86-D3BJ/ZORA-def2-TZVP//r^{2}SCAN-3c, eigenvalues |ν|: electron density transferred, charge flow: orange⟶purple, isovalue ±0.002 a_0^{-3}, hydrogen atoms and *t*Bu methyl groups not shown).

Table S25. Fragmentation patterns for the fragmentation of the C–O bond of $^{\mathbf{T}}$**5** for NOCV analysis with their respective orbital interaction energies ΔE_{orb} (ZORA-BP86-D3BJ/ZORA-def2-TZVP//r^{2}SCAN-3c). All energies in kcal mol^{-1}.

Fragments	^{T}[(PNP)PtC]$^{+}$ ^{S}[OEt]$^{–}$	^{Q}[(PNP)PtC] ^{D}[OEt]	^{T}[(PNP)PtC]$^{–}$ ^{S}[OEt]$^{+}$
ΔE_{orb}	–352.5	–450.5	–660.7

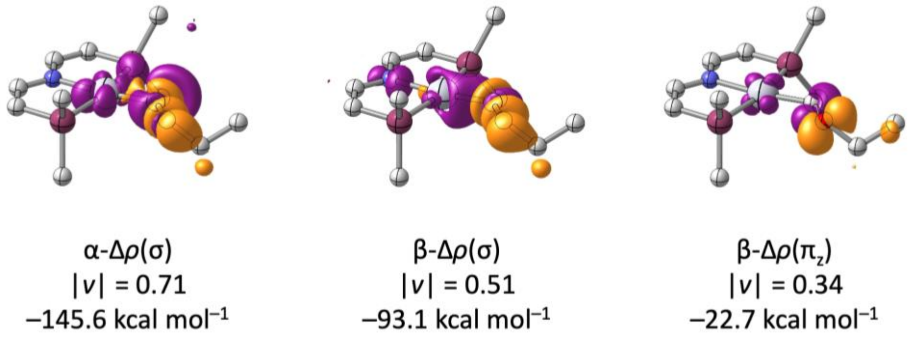

Figure S59. Selected NOCV deformation densities associated with the σ and π interactions of the fragments ^{T}[(PNP)PtC]$^{+}$ and ^{S}[OEt]$^{–}$ of $^{\mathbf{T}}$**5** (ZORA-BP86-D3BJ/ZORA-def2-TZVP//r^{2}SCAN-3c, eigenvalues |ν|: electron density transferred, charge flow: orange⟶purple, isovalue ±0.002 a_0^{-3}, hydrogen atoms and *t*Bu methyl groups not shown).

5.8 Computed Reaction Path

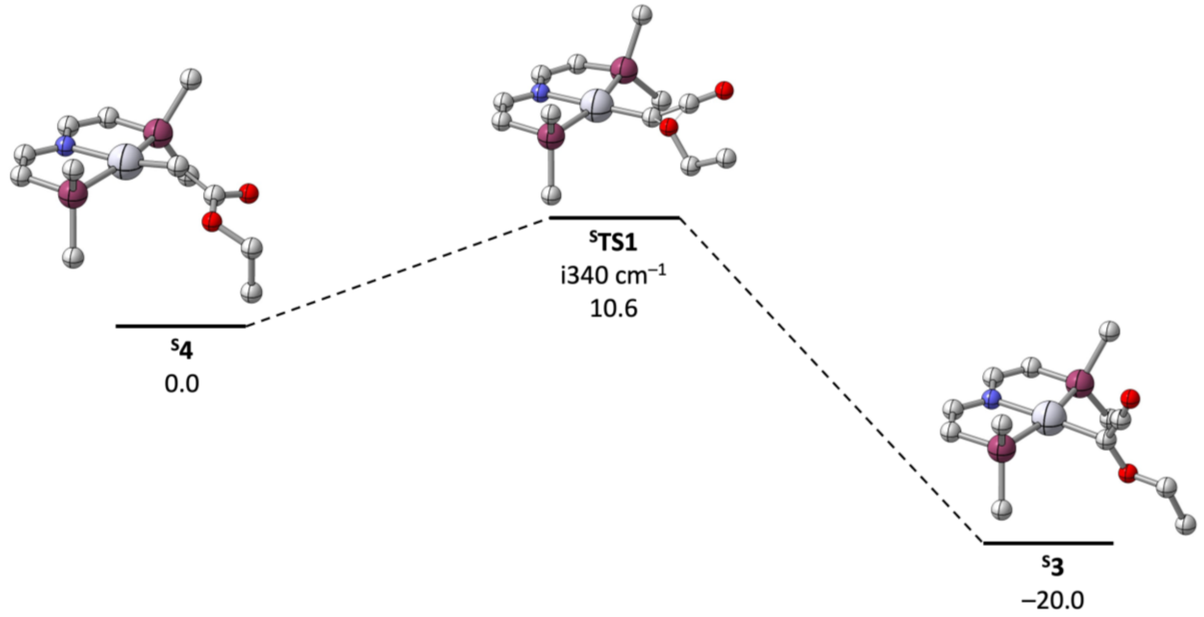

Figure S60. Computed reaction pathway of the intramolecular OEt shift for ^{S}**4** to form **3** (ΔG in kcal mol^{-1}; ONIOM(CCSD(T)-F12/VDZ:r^{2}SCAN-3c)//r^{2}SCAN-3c; hydrogen atoms and methyl groups not shown for clarity)

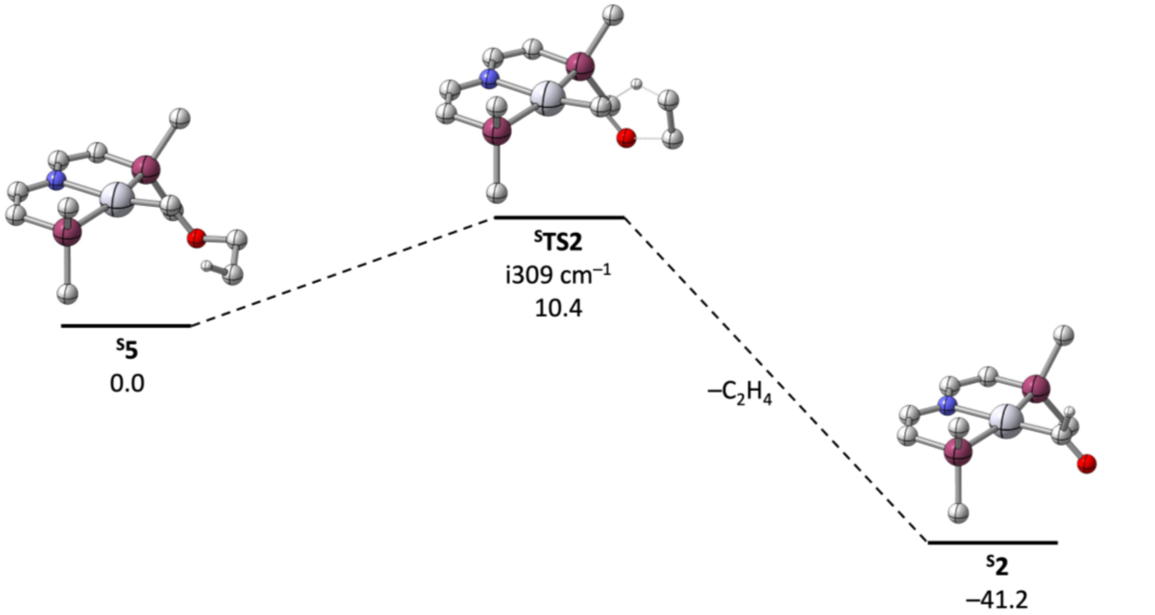

Figure S61. Computed reaction pathway of the intramolecular hydrogen abstraction for ^{S}**5** to form **2** (ΔG in kcal mol^{-1}; ONIOM(CCSD(T)-F12/VDZ:r^{2}SCAN-3c)//r^{2}SCAN-3c; hydrogen atoms not involved in the reaction and methyl groups not shown for clarity)

5.9 Total Energies

Table S26. Total energies E_{tot} (au) for the full and corresponding H-truncated model systems of the carbene intermediates **4** and **5**.

	^{S}**4**	^{T}**4**	^{S}**5**	^{T}**5**
$\Delta G_{corr}^{298.15}$(r^{2}SCAN-3c)	0.584 694	0.582 361	0.575 480	0.573 964
ΔE_{tot}^{full}(r^{2}SCAN-3c)	–1948.671 366	–1948.668 202	–1835.344 114	–1835.313 636
ΔE_{tot}^{full}(mPW2PLYP-D4/def2-QZVPP)	–1948.259 028	–1948.247 529	–1834.937 732	–1834.899 046
ΔE_{tot}^{full}(DLPNO-CCSD(T)/VTZ/PNO[6])	–1945.865 309	–1945.856 378	–1832.698 213	–1832.657 825
ΔE_{tot}^{full}(DLPNO-CCSD(T)/VTZ/PNO[7])	–1945.891 977	–1945.882 335	–1832.723 055	–1832.682 960
ΔE_{tot}^{full}(DLPNO-CCSD(T)/VQZ/PNO[6])	–1946.333 375	–1946.322 707	–1833.131 894	–1833.090 220
ΔE_{tot}^{full}(DLPNO-CCSD(T)/VQZ/PNO[7])	–1946.357 075	–1946.345 734	–1833.154 106	–1833.112 862
ΔE_{tot}^{full}(DLPNO-CCSD(T)/CBS[T/Q]/CPS[6/7])	–1946.664 403	–1946.651 659	–1833.439 804	–1833.398 094
ΔE_{tot}^{H}(r^{2}SCAN-3c)	–1319.783 390	–1319.782 059	–1206.465 157	–1206.436 415
ΔE_{tot}^{H}(CCSD(T*)-F12/VDZ)	–1317.935 702	–1317.925 726	–1204.739 784	–1204.701 802
ΔE_{tot}^{H}(CCSD(T*)-F12/VTZ)	–1318.028 185	–1318.018 332	–1204.817 986	–1204.780 422
ΔE_{tot}^{full}(ONIOM(CC/VDZ:DFT)[a]	–1946.823 678	–1946.811 869	–1833.618 741	–1833.579 024
ΔE_{tot}^{full}(ONIOM(CC/VTZ:DFT)[a]	–1946.916 162	–1946.904 476	–1833.696 943	–1833.657 643

[a] ONIOM(CCSD(T*)-F12/VnZ:r^{2}SCAN-3c), Total energy according to:

$$E_{tot}^{full}(\text{CC:DFT}) = E_{tot}^{full}(\text{DFT}) - E_{tot}^{H}(\text{DFT}) + E_{tot}^{H}(\text{CC})$$

Table S27. Total energies E_{tot} (au) for the full and corresponding H-truncated model systems for the ONIOM calculations of the studied reaction.

	r^{2}SCAN-3c			CCSD(T*)-F12/VDZ	ONIOM(CC:DFT)[a]
Species	ΔE_{tot}^{full}	ΔE_{tot}^{H}	$\Delta G_{corr}^{298.15}$	ΔE_{tot}^{H}	ΔE_{tot}^{full}
^{S}2	–1756.829 009	–1127.950 531	0.522 911	–1126.323 300	–1755.201 777
^{S}3	–1948.698 773	–1319.816 510	0.583 622	–1317.972 176	–1946.854 439
^{S}4	–1948.671 366	–1319.783 390	0.584 694	–1317.935 702	–1946.823 678
^{S}5	–1835.344 114	–1206.465 157	0.575 480	–1204.739 784	–1833.618 741
^{S}TS1	–1948.653 893	–1319.770 431	0.583 455	–1317.922 023	–1946.805 485
^{S}TS2	–1835.331 946	–1206.452 035	0.572 848	–1204.719 648	–1833.599 560
C_2H_4	–78.557 040	–78.557 040	0.029 969	–78.460 007	–78.460 007

[a] ONIOM(CCSD(T*)-F12/VDZ:r^{2}SCAN-3c), Total energy according to:

$$E_{tot}^{full}(\text{CC:DFT}) = E_{tot}^{full}(\text{DFT}) - E_{tot}^{H}(\text{DFT}) + E_{tot}^{H}(\text{CC})$$

Table S28. Total energies E_{tot} (au) for the rotational barrier of **4** on the broken symmetry singlet potential surface.

	r^2SCAN-3c		mPW2PLYP-D4/def2-QZVPP
Species	ΔE_{tot}^{full}	$\Delta G_{corr}^{298.15}$	ΔE_{tot}^{full}
^{S}**4**	–1948.671 366	0.584 694	–1948.259 028
^{BS}**TS3**	–1948.660 032	0.583 619	–1948.238 835

5.10 Cartesian Coordinates r²SCAN-3c Molecular Structures (Å)

1 (^{1}A, C_1), E_{tot} = –2058.214671748935

77

Pt	-2.73649751264919	1.31503515954019	-2.75934811174004
P	-3.04507143010413	2.65251138475967	-4.65757668572252
P	-3.01222027683509	0.16368819505771	-0.72659600021133
N	-4.62134028580680	2.04046940263291	-2.27802583791729
C	-5.22812741992495	2.92519989937378	-3.11441575442662
H	-6.21726721820717	3.28586530601543	-2.81805181842464
C	-4.65437951770291	3.34938559799158	-4.26825501748404
H	-5.16647394812134	4.06035761454355	-4.90683213538915
C	-1.86509229039804	4.13047010116808	-4.79731475946686
C	-1.92912451146440	4.81329771006719	-3.42013351375317
H	-1.25199956723560	5.67700773852848	-3.42291759340357
H	-1.61076948190644	4.12740598169951	-2.62670421568109
H	-2.93906049912206	5.16585932373418	-3.18906191258701
C	-0.41935156319954	3.68629155084095	-5.05123072867819
H	0.23557900186886	4.56214252047864	-4.95828244015190
H	-0.27227351296527	3.27185284514356	-6.04985058863597
H	-0.09498495704099	2.94773016207565	-4.31636519364028
C	-2.30196449599647	5.11970428278424	-5.88322571204123
H	-1.67458643835051	6.01799521217903	-5.81698918724265
H	-3.34351948770377	5.43501141713335	-5.76242431113872
H	-2.17161128571860	4.70539001997873	-6.88726701545679
C	-3.30229976268512	1.74167202517458	-6.30324165588853
C	-1.97916111292506	1.45162599865257	-7.01895035721290
H	-2.16533133490794	0.73515557987801	-7.82879776918869
H	-1.23084107600361	1.00885475087531	-6.35580962427076
H	-1.55864766205970	2.35431363787717	-7.47171659058652
C	-4.24025825450130	2.51571796613928	-7.24092407177422
H	-4.34505075334379	1.94786811428972	-8.17417129812837
H	-3.86106389410029	3.50771893042921	-7.49930972925181
H	-5.23632349013209	2.62676962106194	-6.80353079253722
C	-3.98683229017858	0.41602698546588	-5.92766144713952

H	-4.27318064156079	-0.10946863774917	-6.84761806472789
H	-4.89211192202911	0.58703093486012	-5.33451773715948
H	-3.32013634456459	-0.23433056887953	-5.35538276875633
C	-5.25349384375048	1.58156817609542	-1.16380068466042
H	-6.25838890396788	1.96854737952815	-0.97205195588097
C	-4.68337947318256	0.68602357703845	-0.32008428286267
H	-5.21743854485779	0.34363078316880	0.55909165948497
C	-1.97195256548649	0.76332536678069	0.74436456015023
C	-0.57356925580224	0.13697910599058	0.74052493223655
H	0.06639012826441	0.69048636237549	1.43968535251025
H	-0.10287956995960	0.17592486562485	-0.24559416318987
H	-0.60025512871305	-0.90338414600217	1.07684914318235
C	-1.86683530751646	2.28646299820328	0.55563013174668
H	-1.31015019621968	2.70908123913360	1.40252027054689
H	-2.85890657819684	2.75102508954185	0.53535057478064
H	-1.34591152429973	2.53773045718923	-0.37138542586236
C	-2.65564542307533	0.49565034095762	2.09244402941471
H	-2.01627963759021	0.89505955787057	2.89032435345035
H	-2.80451514908214	-0.56769844308222	2.29250424495108
H	-3.62222824488333	1.00293902558515	2.15779937780972
C	-3.08411738217984	-1.72461984776833	-0.88742664068115
C	-1.72479850073658	-2.32106576653140	-1.27561480094732
H	-1.87068316914125	-3.37236984693962	-1.55344253967768
H	-1.00632047568136	-2.29684104659199	-0.45408252968727
H	-1.28080995910314	-1.80908352215105	-2.13061181167274
C	-4.07870599771659	-1.97811885404833	-2.03380656666167
H	-4.17076291367814	-3.05992807744455	-2.19039514541663
H	-3.73025249610230	-1.52322262093281	-2.96799076795123
H	-5.06966770161238	-1.57312201542560	-1.80606401614313
C	-3.59470011213958	-2.40272476656761	0.38830779081079
H	-3.76613666369488	-3.46648415224068	0.17906197950469
H	-4.54161814192046	-1.97689865221308	0.73578985277852
H	-2.86168786216928	-2.34495700776914	1.19767374054532
C	-0.86080724807142	0.59479643978124	-3.25197230517871

N	-0.80164784121388	-0.39336526994378	-4.09802971715836
N	-0.81552998449416	-1.26274935915402	-4.83243948196579
C	0.45046342367418	0.99832261790076	-2.77755252807223
O	1.52364145116186	0.48647580772165	-3.05829802369148
O	0.34205828225359	2.07463335096538	-1.93306790056232
C	1.58212244219345	2.55614436837409	-1.36875858347634
H	2.23322333169108	1.70370130994164	-1.14892180336447
H	1.28391830364053	3.03754623420378	-0.43097940861296
C	2.26448049785449	3.53864948290366	-2.30014390300011
H	3.16711426080286	3.93707561523236	-1.82416882451595
H	2.55417663822954	3.04326220643782	-3.23084202307966
H	1.59798893759074	4.37508514725657	-2.53339989491159

2 (^{1}A, C_1), E_{tot} = –1756.829008546734

67

Pt	-2.58036959312060	1.80564893235022	-2.50906758170949
P	-2.40144861105738	0.98432200741296	-4.68497144974010
P	-2.99732326192779	3.17528897386511	-0.67026673419927
N	-3.61195569552403	3.38677000146972	-3.52935258158289
C	-3.81879263476092	3.27452910304129	-4.86304859999390
H	-4.36810492142930	4.08032782022515	-5.36223222727304
C	-3.36760064059390	2.20743048456439	-5.58025842693089
H	-3.52999718809977	2.15963078642051	-6.65190772072279
C	-0.64160968850673	1.08648895282262	-5.38772237194574
C	-0.26641176685546	2.57641427983988	-5.31175882089305
H	0.78148850563011	2.69054869079766	-5.61610647608769
H	-0.37104754466967	2.95758433777724	-4.28911648009333
H	-0.89128446309480	3.18679369241301	-5.96968161671954
C	0.34016472837371	0.30226640089480	-4.50724850000797
H	1.35460252410773	0.44593202730247	-4.90125340449762
H	0.14022416788101	-0.77150651625516	-4.49949587625409
H	0.32135442128572	0.65844788462357	-3.47372922235320
C	-0.55443902653294	0.61565036723397	-6.84265399688761
H	0.44371039208904	0.85127917230689	-7.23379698849147

H	-1.28724408170694	1.11994047473300	-7.48214441476109
H	-0.69298049468247	-0.46571475641972	-6.93353385707298
C	-3.24236427385421	-0.68136013612449	-5.00511723600024
C	-2.34154635273686	-1.84912258306446	-4.58952509926421
H	-2.93030674323104	-2.77512721502410	-4.59815713070849
H	-1.93975166548442	-1.71937584584001	-3.57877935826671
H	-1.50456999494241	-1.98502448455856	-5.28066460391898
C	-3.68100447613298	-0.84702993805464	-6.46620150781547
H	-4.19718065415461	-1.81000028761737	-6.57124948799176
H	-2.84056593583549	-0.84541558353457	-7.16442664925748
H	-4.38222567277280	-0.06090320048032	-6.76116532358728
C	-4.50036978938909	-0.66868611638246	-4.11902860008319
H	-5.08269940951609	-1.57901309956738	-4.31102645687987
H	-5.13239190341080	0.19837599205858	-4.33987612578134
H	-4.24113023137182	-0.63709856363841	-3.05579727872051
C	-4.02668333247658	4.45963354232622	-2.81295424388304
H	-4.54417229417465	5.26108868287512	-3.35146914014383
C	-3.81624875223958	4.56080551543682	-1.47122338600903
H	-4.17567189717941	5.42555415749721	-0.92328454696369
C	-1.44183431356116	3.88387188619772	0.14756685720158
C	-0.85977567283592	2.92250193347861	1.18957006133480
H	0.11670943190424	3.30667815011916	1.51147473320088
H	-0.70137235723743	1.92286174831824	0.77426530346348
H	-1.49045049080763	2.85117062134403	2.08105759950148
C	-0.42970713400882	4.04969122417776	-0.99966867937822
H	0.46669389284996	4.55059784500515	-0.61122974319887
H	-0.84049238721499	4.66445653923694	-1.80868132261827
H	-0.13754197059297	3.07742735602159	-1.41011214414986
C	-1.70279728002394	5.25848564014299	0.77766328349929
H	-0.76763216930971	5.62462307491417	1.22024858366945
H	-2.45295816839233	5.22568075849773	1.57201922968091
H	-2.02323704899897	5.98515062941774	0.02528024480721
C	-4.24837793045072	2.48138638038548	0.57449440869290
C	-3.77994147114673	1.12120476825530	1.10703537221115

H	-4.54336818198811	0.72467904279905	1.78858313550010
H	-2.83864068574116	1.17873213054214	1.65704225175252
H	-3.65374824625522	0.40416309927856	0.29062141229334
C	-5.54256881082772	2.26361209254501	-0.22745945665337
H	-6.27236351029806	1.74910453121743	0.41037410453898
H	-5.35991609451739	1.64295907294119	-1.11308245750622
H	-5.97639164813079	3.21004809172909	-0.56181822706096
C	-4.52691689277937	3.44207905545375	1.73563843555358
H	-5.36357504921790	3.05284688409307	2.33016479008481
H	-4.81017320914833	4.43899033732949	1.38119425201714
H	-3.66685840191198	3.53803944899086	2.40444013042854
C	-1.66147450314562	0.31840412565910	-1.55061033764444
O	-0.51457494594168	0.27879893255271	-1.13899201880458
H	-2.30225831416512	-0.59476878572596	-1.37196867319530

3 ([1]A, C_1), E_{tot} = –1948.698773205169

75

Pt	-2.55465881118639	1.83579017809054	-2.53122485016412
P	-2.40621670352693	0.96096700328887	-4.68734983475269
P	-2.96590906252092	3.17288943279674	-0.65413962039538
N	-3.57539177934439	3.36075004152441	-3.50740004075484
C	-3.76121252157022	3.27808605673425	-4.85251370773185
H	-4.29802178999529	4.10312111083476	-5.33017123614058
C	-3.30954726784249	2.22896351845218	-5.58747878560029
H	-3.46665024774000	2.20384874626638	-6.66028301205524
C	-0.63883227573422	0.99349287709295	-5.37641691737451
C	-0.18005642271375	2.45551890841771	-5.24340913546661
H	0.86494755818500	2.52842363288208	-5.57011329543153
H	-0.23979234081653	2.79378969534144	-4.20222600311152
H	-0.78583412699443	3.12826099912101	-5.85735598973057
C	0.29753163343820	0.11876429080638	-4.53174332672275
H	1.31751008968719	0.22103442518874	-4.92544190788785
H	0.03307440092482	-0.93999742053450	-4.56634471043039
H	0.29804915992486	0.43959610974743	-3.48645772601273

C	-0.57802776868150	0.57442055790504	-6.84901802230176
H	0.43678414977679	0.75307662728942	-7.22752084905303
H	-1.26885632841780	1.15523625565119	-7.46973983738479
H	-0.79408424323800	-0.48945179517695	-6.98264329264494
C	-3.31989447351552	-0.66061608851960	-5.04697162801966
C	-2.46591712705011	-1.89074113827486	-4.72150272259587
H	-3.10280947585055	-2.78288840945601	-4.77180519127561
H	-2.04314407149162	-1.84455306014902	-3.71454737804230
H	-1.65500755735096	-2.02569948618706	-5.44375721971629
C	-3.80072742276845	-0.73106487828629	-6.50343144214707
H	-4.33614955040837	-1.67843183078908	-6.64630813479410
H	-2.97995485354334	-0.70466373118156	-7.22502032083020
H	-4.49549841469449	0.08163940904912	-6.73389293030766
C	-4.55538745636584	-0.63447395136007	-4.13023169409613
H	-5.18690494410764	-1.50213920363636	-4.36004410036625
H	-5.14859176133634	0.27297088415710	-4.28990176873459
H	-4.27455310252103	-0.68466331081472	-3.07473407813705
C	-4.01793407204189	4.42930421267492	-2.79047130538472
H	-4.54393905755642	5.21304402946356	-3.34362454586899
C	-3.82559087037147	4.53744472718337	-1.45085589887368
H	-4.20697531457565	5.39706830802857	-0.91058663992641
C	-1.46211392863624	3.96129966136284	0.19204419586666
C	-0.86346474490574	3.04282079676116	1.26271839703033
H	0.10764150014360	3.45156059391836	1.57151437713143
H	-0.69507281062713	2.03383849606318	0.87479963522540
H	-1.49439462918587	2.98990812128483	2.15515568936860
C	-0.43364064301624	4.16739455053086	-0.93361188140588
H	0.42653165327782	4.71891925332654	-0.53143611867135
H	-0.85753480625276	4.75350649031324	-1.75712711039295
H	-0.08344404456957	3.20873677329488	-1.32697307618378
C	-1.80087726524538	5.32820285700594	0.80253636393671
H	-0.89812176386878	5.73093207151455	1.27965445743937
H	-2.58111455812859	5.27192236715752	1.56614958792902
H	-2.11677656895383	6.03887773763312	0.03342942679935

C	-4.21254045207870	2.43411788162458	0.57176272636928
C	-3.68223030597963	1.13547640185055	1.19220919187932
H	-4.47437708844575	0.69424500317727	1.81065680023423
H	-2.81318795887060	1.29806986647973	1.83273280768074
H	-3.40595841459936	0.41083461543883	0.42207096733270
C	-5.45016588190844	2.09218947442944	-0.27565591298318
H	-6.19054011370822	1.59724080441781	0.36516564154398
H	-5.19240474408834	1.40945963440102	-1.09371296847420
H	-5.90526715409596	2.98782722254631	-0.70845414194658
C	-4.61105184163005	3.42261007454480	1.67284360948937
H	-5.43954605395602	2.99357317359377	2.25114649620095
H	-4.95486759086282	4.37841206863940	1.26308961355973
H	-3.79061046624917	3.61297262496379	2.37100587455971
C	-1.45972594369486	0.38908754115269	-1.57285391537048
C	-2.03089115916091	-0.76686647172858	-1.34883000211476
O	-2.65576877593539	-1.77146342817083	-1.21255755109474
O	-0.17008583727726	0.72494097032978	-1.14234800432015
C	0.54118366628551	-0.35384870242004	-0.53224112813814
H	0.59839806949419	-1.19798814343075	-1.24078150041660
H	-0.01340227706426	-0.70050428848779	0.35713771731100
C	1.91965175255316	0.14511428687218	-0.16072428621103
H	2.49645553735863	-0.65598132529432	0.31213022321640
H	2.45845280590784	0.48144409324123	-1.05185632811887
H	1.85171790294107	0.98320249513184	0.53988711649820

^{S}**4** (^{1}A, C_1), E_{tot} = –1948.671365922010

75

Pt	-1.72793282207842	-0.54003701721381	-4.81684842328430
P	-2.42843636904261	-1.03752835051830	-2.63885542170589
P	-1.26839631814551	-0.79108652120062	-7.10876092689228
N	-3.06454517277701	-2.14978644199478	-5.24297066636645
C	-3.80845362282434	-2.68310137886821	-4.24286771869245
H	-4.51445099135773	-3.47546662443834	-4.50968947593598
C	-3.69289013204865	-2.26461494119101	-2.95445749358395

H	-4.28149375164387	-2.72056968025934	-2.16608010296723
C	-1.07633699590811	-1.91011078567740	-1.62940295892735
C	-0.78272435221660	-3.21901235962085	-2.38087196682779
H	0.07280501908213	-3.71222748863179	-1.90278336123290
H	-0.52675809553872	-3.02269070281560	-3.42822705518650
H	-1.63688836329489	-3.90140306743864	-2.35932697345407
C	0.20482769901608	-1.07121471402878	-1.60464303820058
H	0.97804003484218	-1.62717928087346	-1.05844946010571
H	0.07408124088684	-0.10347976617170	-1.11737655847842
H	0.56766850498236	-0.88336747587884	-2.61952556875044
C	-1.53120340699459	-2.23702046242516	-0.20327446269770
H	-0.77119043895064	-2.86954766187412	0.27297194274748
H	-2.47630417438481	-2.79092430117319	-0.18885597488422
H	-1.63944381713387	-1.33839129988781	0.41063662978272
C	-3.27646840880687	0.38250843049784	-1.71945994573402
C	-2.25377563536332	1.34576349570058	-1.11142801709345
H	-2.78339497274701	2.23453865576395	-0.74607505747666
H	-1.51613724928270	1.67918935080711	-1.84913088377039
H	-1.72597568002573	0.90477240145339	-0.26013999066278
C	-4.23488080810011	-0.13971404863166	-0.63947260319754
H	-4.71052523467317	0.72064231445411	-0.15247138512751
H	-3.73024420344323	-0.71983678031281	0.13640800130795
H	-5.02919121601159	-0.75281066387614	-1.07540508948901
C	-4.09953112994194	1.12731930383205	-2.78457427114795
H	-4.69452626257933	1.90366805879157	-2.28682011356269
H	-4.78628847646374	0.45010476441917	-3.30477184850427
H	-3.45034925950711	1.60160716897721	-3.52643621719883
C	-3.16777322650443	-2.60815776434900	-6.51637793489468
H	-3.88685742244513	-3.41139269279692	-6.70375244588216
C	-2.41449404522503	-2.10574159089305	-7.52786661816534
H	-2.52364766940409	-2.48595369904756	-8.53746855136869
C	0.45733803628096	-1.45865535987637	-7.54398418573909
C	1.48394441106943	-0.34364859760915	-7.76424023343104
H	2.47774128215986	-0.80372005390991	-7.84180266532418

H	1.50277020503134	0.36941089568696	-6.93555948578084
H	1.30478634001138	0.19640396875027	-8.69895035285610
C	0.88361156319521	-2.30762422473883	-6.33581928223331
H	1.83097098683239	-2.80997145371424	-6.57054187442873
H	0.13685077088377	-3.07450720517583	-6.10032102868021
H	1.02607607688824	-1.68189408858821	-5.44898596381316
C	0.39021802021133	-2.36303328574810	-8.78346206315211
H	1.40424496867748	-2.71729701645314	-9.00786158367124
H	0.02159405910911	-1.83722508266128	-9.66903419214002
H	-0.24230639567045	-3.23788722677548	-8.60900948403095
C	-1.78565059547554	0.70694289126566	-8.14812679607283
C	-0.99734710426259	1.96325689979636	-7.76379426262803
H	-1.41899639617673	2.81522069510263	-8.31245081519019
H	0.06299579501913	1.89566051088387	-8.01009775980608
H	-1.08258698177892	2.17400650310937	-6.69179047231793
C	-3.26720916243530	0.94231119442955	-7.80776654231122
H	-3.61833547537012	1.81812524313582	-8.36731044607316
H	-3.39600074542184	1.14359654031845	-6.73836057557798
H	-3.89066425705566	0.08392141107260	-8.07529195883640
C	-1.64502253064226	0.42150883898921	-9.64856077772841
H	-2.07753879701257	1.26036386009571	-10.20832054706116
H	-2.18114442229402	-0.48566174458903	-9.94796476707670
H	-0.59878962906525	0.33074956848227	-9.95434344603341
C	-0.80121585605029	1.06598564794038	-4.49425405497553
C	0.51028851751774	1.50211581105036	-4.23026196895506
O	1.30168862757293	1.73013398790433	-5.15619158219393
O	0.77150810011209	1.82401894205967	-2.92345952211948
C	2.06476734811348	2.42264425083603	-2.67518093247013
H	1.92716512130523	2.99502818649440	-1.75229446291459
H	2.30915119660917	3.10932252255557	-3.49207057865672
C	3.13747729934113	1.36240703106628	-2.51226937666010
H	4.09241394539689	1.83296485028378	-2.25271470288110
H	2.87022617034485	0.66124969081407	-1.71513745257902
H	3.26858788742477	0.80590347565192	-3.44430664978148

^{T}**4** (^{3}A, C_1), E_{tot} = –1948.668202681989, <S^{2}> = 2.02

75

Pt	-2.800618	1.421117	-3.462652
P	-2.424412	1.731515	-5.744680
P	-3.731504	1.356234	-1.322724
N	-4.709959	2.117036	-3.971129
C	-4.987528	2.395951	-5.273810
H	-5.998168	2.746340	-5.504317
C	-4.064436	2.252359	-6.261575
H	-4.313556	2.502236	-7.287403
C	-1.250547	3.163239	-6.148213
C	-1.920284	4.416780	-5.559930
H	-1.235034	5.265962	-5.673970
H	-2.132058	4.287991	-4.491999
H	-2.858792	4.654133	-6.068723
C	0.100690	2.969746	-5.448087
H	0.734638	3.838853	-5.667202
H	0.632744	2.078073	-5.787073
H	-0.022099	2.900623	-4.363880
C	-1.050247	3.355101	-7.655255
H	-0.498052	4.288749	-7.822878
H	-2.001897	3.434199	-8.192288
H	-0.462176	2.545693	-8.097151
C	-2.037785	0.135118	-6.682661
C	-0.562707	-0.249617	-6.528423
H	-0.419186	-1.264652	-6.919816
H	-0.253706	-0.249662	-5.476661
H	0.095990	0.415722	-7.094761
C	-2.422646	0.227098	-8.165309
H	-2.230561	-0.744801	-8.637371
H	-1.843573	0.976203	-8.709827
H	-3.487104	0.449851	-8.287938
C	-2.911066	-0.943837	-6.017832

H	-2.798584	-1.881769	-6.576370
H	-3.970532	-0.664010	-6.027145
H	-2.605956	-1.115508	-4.981143
C	-5.637368	2.278099	-2.988557
H	-6.621911	2.650463	-3.287290
C	-5.372664	1.994663	-1.686006
H	-6.140343	2.120689	-0.930084
C	-2.970776	2.549520	-0.063681
C	-1.757295	1.932040	0.638785
H	-1.250880	2.717057	1.215067
H	-1.034818	1.531284	-0.078121
H	-2.045710	1.144936	1.341538
C	-2.509866	3.765857	-0.884950
H	-2.153230	4.543060	-0.196403
H	-3.332151	4.184888	-1.476024
H	-1.693393	3.495747	-1.561643
C	-4.004716	3.012235	0.971918
H	-3.514082	3.701082	1.671184
H	-4.417096	2.186392	1.557649
H	-4.830962	3.550218	0.497984
C	-3.970017	-0.401318	-0.655453
C	-2.626908	-1.136429	-0.552571
H	-2.821078	-2.184692	-0.292556
H	-1.974734	-0.720338	0.217624
H	-2.085864	-1.111292	-1.503953
C	-4.833592	-1.117725	-1.708138
H	-4.963028	-2.163628	-1.403439
H	-4.351146	-1.104487	-2.692331
H	-5.821319	-0.657934	-1.805292
C	-4.697381	-0.418831	0.693299
H	-4.931208	-1.458082	0.957833
H	-5.643248	0.132993	0.656758
H	-4.081886	-0.008067	1.498932
C	-1.061903	0.697161	-3.039544

C	0.260403	0.776878	-2.544726
O	1.076821	-0.144530	-2.544868
O	0.574736	2.033865	-2.038443
C	1.936928	2.202506	-1.589087
H	2.260987	1.297019	-1.064885
H	1.892166	3.036358	-0.880374
C	2.862124	2.516741	-2.750621
H	3.879452	2.693356	-2.384095
H	2.888853	1.677976	-3.451548
H	2.524495	3.412987	-3.280854

^{S}**5** (^{1}A, C$_1$), E_{tot} = –1835.344114343734

73

Pt	-2.55248080930851	1.82543700571422	-2.42569241127834
P	-2.24946746706993	1.06431421145748	-4.61873826692511
P	-3.02994310772975	3.27072084914197	-0.65785837484907
N	-3.78849820524634	3.28754839422937	-3.49173145928982
C	-3.91242772282523	3.18050271924888	-4.83411808752086
H	-4.52224155265872	3.92878327300640	-5.35418986343822
C	-3.29988549274768	2.18932436259038	-5.54243909247732
H	-3.41225150422541	2.13596219365543	-6.62045226447250
C	-0.48983312210851	1.29397618070795	-5.29022573008119
C	-0.24427190393037	2.81179159770351	-5.27121977112659
H	0.80268540871601	3.00401389818663	-5.53810146642443
H	-0.43144079676240	3.22592089959882	-4.27357931651261
H	-0.88989663874955	3.33473488472924	-5.98222462277226
C	0.52180337060280	0.63495103167275	-4.34327108484595
H	1.53703638847290	0.84835716613020	-4.70320851771350
H	0.41011491586321	-0.45100977200335	-4.29406356749928
H	0.42283482563141	1.03320612401978	-3.32919010352062
C	-0.31016999512538	0.76902444810889	-6.71752800839886
H	0.67606343791512	1.07495974689914	-7.09087646235947
H	-1.06276272596593	1.17630316025928	-7.40150344357338
H	-0.35126762146702	-0.32321227106169	-6.76195795140739

C	-2.95153252392612	-0.66867548978322	-4.93371101818565
C	-1.98990772237928	-1.74985001367108	-4.43439717618801
H	-2.50075427125278	-2.72110640397486	-4.45573253203129
H	-1.69003044535260	-1.55614464425138	-3.39743327243389
H	-1.09811531676699	-1.83233385994126	-5.06355384508154
C	-3.31179516386936	-0.90329752715012	-6.40575711296093
H	-3.77703761134958	-1.89306087998427	-6.49965132441488
H	-2.44239577922220	-0.88540715983268	-7.06728265807589
H	-4.03589470245425	-0.16292487211703	-6.75892179551857
C	-4.24189748897173	-0.72656702241835	-4.09646183886687
H	-4.75155890322531	-1.67853035556742	-4.29541405121263
H	-4.92404702405883	0.08948424255273	-4.35980732823273
H	-4.01893871434696	-0.66359846160659	-3.02665449219403
C	-4.29255962014719	4.33382706758787	-2.79956797010192
H	-4.89361138878686	5.06951513282056	-3.34688771207311
C	-4.06406002184278	4.49774644512319	-1.46526734173518
H	-4.49823514050841	5.33806606283887	-0.93383570879086
C	-1.53543105830148	4.22836331198816	0.01478782497300
C	-0.75311570312742	3.39579751702230	1.03638444235337
H	0.19704656340561	3.90117790887132	1.25332098144617
H	-0.52355099497734	2.39866670511921	0.64756968159574
H	-1.29249794587575	3.29434571688311	1.98267780929511
C	-0.64056514328466	4.48582768602961	-1.20880649903473
H	0.21286984484096	5.10578212102379	-0.90359551555969
H	-1.18706469501952	5.01500134632231	-1.99727681682583
H	-0.26764524411250	3.54466154871609	-1.62621545778148
C	-1.93474331667400	5.57908534340883	0.62236448893790
H	-1.02819666433121	6.09384725222527	0.96644830926166
H	-2.60240121228440	5.47559325813253	1.48112236599311
H	-2.41983502542418	6.21825910888050	-0.12113725511135
C	-4.09594793303973	2.50427827297984	0.71528750083899
C	-3.41716387834510	1.27094774396642	1.32332553655503
H	-4.12037832285324	0.78813887063693	2.01423161261675
H	-2.51546326509111	1.51643888645285	1.88993896444251

H	-3.15348150761513	0.54330533171428	0.54537139733751
C	-5.37834021774188	2.03972518313237	0.00278877081009
H	-6.01548545563073	1.51680522895878	0.72710385491578
H	-5.14670745658798	1.34723060833786	-0.81492237932620
H	-5.93774845245112	2.88320539479705	-0.41212144040532
C	-4.46227345906966	3.50795271467698	1.81361435816487
H	-5.19993713344450	3.04922585559994	2.48482313506867
H	-4.91152222214853	4.41910545487901	1.40348219082379
H	-3.59630527137015	3.78537224893518	2.42200753046280
C	-1.80095666733873	0.33844429969976	-1.45226919070887
O	-0.55208729710029	0.42355985774089	-1.02587416151049
C	-0.06096193007459	-0.72732793301878	-0.26415336830158
H	-0.93787773646382	-1.28910799146681	0.07770586408795
H	0.47472417394110	-0.30488123004185	0.59366007950804
C	0.84750004765974	-1.56417273462299	-1.13525471675194
H	1.27581109303167	-2.38500700763467	-0.55019211087027
H	0.28432733381449	-1.99248553862043	-1.97029372011638
H	1.66741908639618	-0.96138142935431	-1.53801157889939

^{T}**5** (^{3}A, C_1), E_{tot} = –1835.313635981051, <S^2> = 2.01

73

Pt	-2.307554	2.016659	-2.534748
P	-2.219117	1.084191	-4.684080
P	-2.890022	3.299590	-0.685293
N	-3.618515	3.360119	-3.512076
C	-3.892017	3.184994	-4.830396
H	-4.579791	3.896974	-5.297899
C	-3.347715	2.171804	-5.557932
H	-3.573260	2.072058	-6.614416
C	-0.535066	1.258465	-5.549290
C	-0.245404	2.769847	-5.540122
H	0.750192	2.939876	-5.969033
H	-0.254987	3.167285	-4.518538
H	-0.979562	3.326739	-6.129712

C	0.571054	0.553918	-4.753787
H	1.542898	0.822655	-5.187785
H	0.484663	-0.534625	-4.788063
H	0.556613	0.865966	-3.703206
C	-0.550829	0.756100	-6.996543
H	0.388319	1.047173	-7.485000
H	-1.373024	1.191632	-7.575032
H	-0.620850	-0.334300	-7.050069
C	-2.956459	-0.656562	-4.830111
C	-1.953335	-1.713432	-4.357647
H	-2.473278	-2.672301	-4.234801
H	-1.514640	-1.434564	-3.391594
H	-1.148961	-1.866792	-5.083169
C	-3.446767	-0.972318	-6.248874
H	-3.902526	-1.971042	-6.251180
H	-2.640308	-0.975975	-6.985386
H	-4.210572	-0.258440	-6.572153
C	-4.170018	-0.659621	-3.884669
H	-4.688857	-1.622930	-3.975688
H	-4.875871	0.138311	-4.140421
H	-3.859230	-0.517809	-2.844520
C	-4.181180	4.367426	-2.795823
H	-4.848500	5.051305	-3.329740
C	-3.938631	4.532800	-1.468624
H	-4.416354	5.337655	-0.920458
C	-1.501688	4.285637	0.161525
C	-0.793547	3.479730	1.256291
H	0.087917	4.043788	1.587779
H	-0.454989	2.508160	0.887331
H	-1.430270	3.327956	2.133127
C	-0.493727	4.603091	-0.955613
H	0.295575	5.249331	-0.549602
H	-0.973106	5.128753	-1.789257
H	-0.037670	3.688888	-1.347942

C	-2.018652	5.610409	0.740171
H	-1.174780	6.143675	1.196597
H	-2.777406	5.471093	1.515018
H	-2.433072	6.251411	-0.042998
C	-4.012723	2.405920	0.562126
C	-3.329425	1.135824	1.082670
H	-4.030062	0.598429	1.734978
H	-2.424057	1.343657	1.657785
H	-3.059737	0.481372	0.248257
C	-5.258413	1.984519	-0.234717
H	-5.905727	1.382012	0.415241
H	-4.982578	1.380655	-1.107130
H	-5.824980	2.851002	-0.587026
C	-4.444505	3.304226	1.725341
H	-5.192226	2.772712	2.328590
H	-4.907334	4.231864	1.371518
H	-3.611713	3.558453	2.387061
C	-0.984657	0.816850	-1.748750
O	-0.503187	0.629163	-0.504334
C	-0.408651	-0.751445	-0.071632
H	-1.353584	-1.258958	-0.311625
H	-0.296476	-0.702727	1.017318
C	0.770614	-1.450228	-0.718963
H	0.845774	-2.480662	-0.354734
H	0.648784	-1.471111	-1.806458
H	1.703081	-0.927747	-0.484682

C_2H_4 (^1A_g, D_{2h}), E_{tot} = –78.557039654832

6

C	-0.66341490277848	0.00000000020616	0.00000000013241
C	0.66341490284458	-0.00000000074641	0.00000000008124
H	-1.23297695504826	0.92541077790316	0.00000000003921
H	-1.23297695498313	-0.92541077725833	-0.00000000015989
H	1.23297695417022	0.92541077789872	-0.00000000014604

H	1.23297695579507	-0.92541077800330	0.00000000005307

^{S}TS1 (^{1}A, C_1), E_{tot} = –1948.653892834729

75

Pt	-2.61888904169503	-0.09687085783283	-1.64737180543733
P	-3.73162697728564	0.13403280011273	-3.69832658278051
P	-2.14312741671835	-0.47433851449914	0.61648144795200
N	-4.53831494350183	-0.65354510798172	-1.01611905298237
C	-5.53192992876764	-0.78378483074972	-1.93474455889060
H	-6.50889102682350	-1.10813591842972	-1.56539538196068
C	-5.33914344322921	-0.52467599848403	-3.25243948018363
H	-6.15893100739207	-0.60858235973903	-3.95717946627659
C	-4.06392091877245	1.93597954616337	-4.20337773681223
C	-4.86649369145263	2.54568751362547	-3.04140609841774
H	-5.00164084429732	3.61673979861669	-3.23666470006681
H	-4.33088650313897	2.43465432241545	-2.09137328399327
H	-5.85149800477131	2.08296470973450	-2.93615432973549
C	-2.75935753781432	2.72527628986231	-4.34591172287631
H	-3.00711985087756	3.76333391362080	-4.60176202988754
H	-2.09726816714596	2.33942897505835	-5.12263873694570
H	-2.20186579123290	2.73237893180610	-3.40546254494270
C	-4.88910681997359	2.02603140315575	-5.49144635672942
H	-5.17785234115353	3.07254257673098	-5.65260087464417
H	-5.81000334680923	1.43560427939484	-5.43289683867296
H	-4.31916433115295	1.70675339391662	-6.36851436166464
C	-3.09559029673624	-0.94858454950655	-5.11671599515413
C	-1.96154904138527	-0.27233662553898	-5.89309070674026
H	-1.53114956031477	-1.00288587334295	-6.58971539770934
H	-1.15545943233530	0.07556909723480	-5.24075361305770
H	-2.31703589594550	0.57459106938814	-6.48717875052231
C	-4.22980037128619	-1.34012150826888	-6.07564894946517
H	-3.80921494969183	-1.96762626380951	-6.87145411823055
H	-4.70249253265995	-0.47792558502785	-6.55245422935487
H	-5.00046170134792	-1.92351015918087	-5.56376151367299

C	-2.56080647452437	-2.22642300448249	-4.44804792257578
H	-2.27719948392425	-2.94175830453484	-5.23072756857663
H	-3.32247694533975	-2.69639547834680	-3.81514050810725
H	-1.68377171164940	-2.01140492250913	-3.83018119667625
C	-4.77527402717222	-0.88264628721932	0.30216996092578
H	-5.79838094927480	-1.14626968499233	0.58464437831933
C	-3.79598403218013	-0.79508608355326	1.23688894525423
H	-4.00688121115070	-1.00669237581100	2.27897210433321
C	-1.48191358311805	0.98089810260372	1.63808754696670
C	0.04813239033626	1.04912433153354	1.65171494750741
H	0.34725038225557	1.98833314727209	2.13438503296602
H	0.45785447207796	1.05088385257999	0.63894207522204
H	0.49144539604279	0.23168538677224	2.22806526604540
C	-2.03528545948837	2.24106479961062	0.95295015434133
H	-1.77480794678173	3.11823319891049	1.55926310097990
H	-3.12670002986985	2.20078923180493	0.86174706982653
H	-1.60910254761637	2.36596867734235	-0.04736603689310
C	-2.01070063515468	0.92592822171874	3.07912496337160
H	-1.59387705907716	1.77580224049325	3.63409014098637
H	-1.71427172354704	0.01268163038552	3.60336211213016
H	-3.10063994281934	1.00774957132546	3.10981580795895
C	-1.19936383651973	-2.09671092048324	0.91015070748769
C	0.17477855968705	-2.05456732312744	0.23782959584076
H	0.63442688695169	-3.04934357256404	0.30028635679948
H	0.84996450922385	-1.34672411224279	0.72269746373494
H	0.08443702717738	-1.78251185996805	-0.81915083614629
C	-2.03790886852874	-3.18822341941417	0.22365238153826
H	-1.50423699807710	-4.14334067194784	0.30508135218808
H	-2.18593896244526	-2.96365090063589	-0.83898617728882
H	-3.02103817305577	-3.29805966988203	0.68973818575431
C	-1.06379792630934	-2.42604983801704	2.40057437006209
H	-0.62842056602845	-3.42810167834665	2.50649509003063
H	-2.03642451443806	-2.43501184321818	2.90409493026269
H	-0.40454204252202	-1.72613648625587	2.92223601475174

C	-0.88463265199355	0.36005106037352	-2.40739111693278
C	-0.12296612960141	1.20631743872875	-3.09586634733086
O	0.34492826302402	1.78907639509340	-4.03190008601453
O	0.61925966479342	1.07957219176893	-1.62477565302930
C	1.83955275202769	0.32100292975674	-1.76100031085357
H	1.72894787599436	-0.40133859083764	-2.58135524302260
H	1.99071920392696	-0.24007424370296	-0.83048806196485
C	2.99564892802551	1.27244004731064	-2.01122938965104
H	3.93488514267330	0.71037624622259	-2.06974378375514
H	2.84752476874899	1.81665553812645	-2.94706067670254
H	3.07895612229989	1.99691023611395	-1.19492824860217

^{S}**TS2** (^{1}A, C$_1$), E_{tot} = –1835.331945984424

73

Pt	-2.44157317650631	1.90033351143047	-2.49654538544646
P	-2.16488768998807	1.11921261085201	-4.67710049403540
P	-2.97356296156121	3.25356599973876	-0.68021342341768
N	-3.78441222697273	3.24423319314523	-3.49263766130178
C	-3.96537256191951	3.11794847645623	-4.82968320693415
H	-4.65124259574027	3.82023131355719	-5.31594672816658
C	-3.32405316156370	2.16565823830108	-5.56331173000122
H	-3.48212350848943	2.09966814841913	-6.63479148426135
C	-0.45247636712202	1.44732313579174	-5.42306102260486
C	-0.30133675015807	2.97723780367526	-5.43478386482237
H	0.71815324405673	3.22920828461479	-5.75305570856972
H	-0.46643586183034	3.39509511424259	-4.43480305055683
H	-1.00964926413403	3.44906534156865	-6.12162455302573
C	0.63717777505637	0.86512131384168	-4.51272204436240
H	1.62018898326929	1.12676694968780	-4.92568968669141
H	0.58802274066200	-0.22393412410246	-4.43899539935623
H	0.56490491603027	1.27672110950714	-3.50214159122576
C	-0.30145496791911	0.91128627794005	-6.84967600789799
H	0.64606102649355	1.27369339197956	-7.26961257782483
H	-1.10715239602233	1.25757584943846	-7.50639136958578

H	-0.27254477536980	-0.18194737217329	-6.87616660022861
C	-2.76512163632330	-0.66092209240528	-4.93363580148570
C	-1.71715167665344	-1.67191422535798	-4.45892895269467
H	-2.17240207857183	-2.67027839228644	-4.43305889827516
H	-1.37103357729332	-1.43957173307595	-3.44459900317765
H	-0.85545442927972	-1.72119959642368	-5.13177709896372
C	-3.16909555933547	-0.94413901269439	-6.38586000145969
H	-3.57535325387941	-1.96209685260294	-6.44561485223546
H	-2.32993333229956	-0.88450654410322	-7.08293406593589
H	-3.95138545891739	-0.25593325451757	-6.72027595016039
C	-4.01547391578003	-0.78689019687505	-4.04442475128637
H	-4.46553398286472	-1.77557453361122	-4.20355598021214
H	-4.76203703832589	-0.02550711147496	-4.29628076049732
H	-3.75711153546920	-0.68337337158726	-2.98572567722138
C	-4.35564543438989	4.24226068530452	-2.77643466000786
H	-5.02838727999167	4.92528906339562	-3.30633948212504
C	-4.11080915514791	4.41410864414569	-1.44735188242579
H	-4.59698109055074	5.21456290047344	-0.89976203599145
C	-1.53929911230241	4.30133473913028	-0.01466673575187
C	-0.66897612922201	3.50246525245444	0.96145814017871
H	0.24592103542710	4.07251587911101	1.16882669186004
H	-0.37447030502166	2.53610784374140	0.53956087415932
H	-1.17286569643023	3.33585111951413	1.91821486042509
C	-0.69981064105534	4.66206345116942	-1.25221027689929
H	0.10895761848455	5.34022881379583	-0.94971620546144
H	-1.30621561533660	5.16670511581709	-2.01242700324489
H	-0.26311193658473	3.76636472703913	-1.70584250482664
C	-2.02338411863452	5.60019044435685	0.64222069048567
H	-1.14874979985366	6.17405937982106	0.97509747357036
H	-2.65464970000053	5.42397584097986	1.51657133837391
H	-2.57781247004321	6.22012129925172	-0.06852133071107
C	-3.95212001331870	2.37448613491845	0.68902821994276
C	-3.16637588489841	1.18597972199448	1.25795069717785
H	-3.81074926452511	0.64421547123184	1.96231934723512

H	-2.26835537376016	1.49147918350022	1.79950577393426
H	-2.87327198193446	0.49084576408878	0.46248694685987
C	-5.20641824439761	1.82577725697682	-0.01330046274748
H	-5.78711374032836	1.23888569073665	0.70936409982233
H	-4.93558841163786	1.17190256287438	-0.85056824461599
H	-5.83816278360100	2.63073373043952	-0.39982473805299
C	-4.37586664484347	3.32099936188697	1.81714314018761
H	-5.07354794980420	2.79546120952343	2.48201338914167
H	-4.88998203659075	4.20960332833952	1.43499715938877
H	-3.52454535737173	3.64139617035555	2.42505503037335
C	-1.49184330770740	0.46463910006366	-1.55504784790721
O	-0.29936476691482	0.55076736446100	-1.20754405414886
C	0.35645526063944	-0.86729698937893	-0.34119544011130
H	0.65489655666280	-0.35614044802618	0.57080714164149
H	1.19131650707474	-1.06887928580159	-1.00759030509514
C	-0.72661577310829	-1.85173524953007	-0.26324845869184
H	-1.05275838237850	-2.07081963378354	0.75602127926351
H	-1.59857048357152	-1.33488078795750	-0.79348692450566
H	-0.52093003830900	-2.77924950731120	-0.80255431677708

[BS]**TS3** (^{1}A, C$_1$), E_{tot} = –1948.660032027314, <S^2> = 1.01

75

Pt	-2.834476	1.729804	-3.356036
P	-2.363325	1.908523	-5.636435
P	-3.894944	1.507619	-1.288108
N	-4.802559	1.991748	-4.018807
C	-5.033592	2.148720	-5.350971
H	-6.072984	2.282852	-5.665128
C	-4.030784	2.140229	-6.268440
H	-4.250079	2.280038	-7.321537
C	-1.382529	3.460484	-6.101238
C	-2.214396	4.639056	-5.566681
H	-1.669575	5.571232	-5.760736
H	-2.373761	4.552483	-4.485768

H	-3.192494	4.698382	-6.053056
C	-0.015612	3.477150	-5.404487
H	0.460729	4.448221	-5.591296
H	0.658163	2.701735	-5.774715
H	-0.119296	3.347934	-4.321682
C	-1.215727	3.603912	-7.618281
H	-0.769228	4.582244	-7.837854
H	-2.176959	3.554806	-8.141458
H	-0.550988	2.840526	-8.032465
C	-1.707400	0.324834	-6.447102
C	-0.182038	0.219913	-6.348707
H	0.118348	-0.785572	-6.669803
H	0.166522	0.348158	-5.320090
H	0.324889	0.937191	-7.000949
C	-2.154265	0.220510	-7.911517
H	-1.763581	-0.716275	-8.328706
H	-1.777173	1.039585	-8.530225
H	-3.244710	0.193873	-7.994409
C	-2.335950	-0.829758	-5.647894
H	-2.086327	-1.777430	-6.142413
H	-3.427891	-0.742666	-5.607316
H	-1.943245	-0.855087	-4.626350
C	-5.815724	1.979961	-3.109847
H	-6.830503	2.124778	-3.492512
C	-5.598602	1.796602	-1.780135
H	-6.430674	1.776966	-1.084286
C	-3.453069	2.860403	-0.043059
C	-2.113403	2.563441	0.638483
H	-1.795477	3.454246	1.194958
H	-1.332683	2.329312	-0.095146
H	-2.188710	1.739666	1.354888
C	-3.316825	4.145774	-0.877718
H	-3.138210	4.990098	-0.199661
H	-4.229023	4.351241	-1.448833

H	-2.478593	4.073950	-1.577621
C	-4.557421	3.070625	1.001570
H	-4.265099	3.901182	1.656637
H	-4.716848	2.193838	1.633081
H	-5.507072	3.339968	0.528942
C	-3.811605	-0.246424	-0.578951
C	-2.354498	-0.712396	-0.459204
H	-2.349018	-1.743281	-0.082428
H	-1.768921	-0.104099	0.234074
H	-1.849332	-0.702781	-1.428924
C	-4.518754	-1.138588	-1.613297
H	-4.407139	-2.185838	-1.306322
H	-4.069598	-1.024080	-2.606548
H	-5.585188	-0.907938	-1.689449
C	-4.527445	-0.367732	0.770453
H	-4.584501	-1.428414	1.046554
H	-5.551737	0.019308	0.731680
H	-3.985524	0.148113	1.568240
C	-1.025883	1.593160	-2.734601
C	0.147227	0.822821	-2.600346
O	0.234736	-0.386751	-2.861539
O	1.221501	1.552064	-2.133931
C	2.472701	0.838908	-2.055801
H	2.297915	-0.164131	-1.651134
H	3.072591	1.416488	-1.345139
C	3.152592	0.766680	-3.411792
H	4.139139	0.300199	-3.313096
H	2.557149	0.167904	-4.106485
H	3.284449	1.770564	-3.828257

6. Supplementary References

(1) Sun, J.; Abbenseth, J.; Verplancke, H.; Diefenbach, M.; de Bruin, B.; Hunger, D.; Würtele, C.; van Slageren, J.; Holthausen, M. C.; Schneider, S. A Platinum(II) Metallonitrene with a Triplet Ground State. *Nat. Chem.* **2020**, *12*, 1054−1059.

(2) Willett, K. L.; Hites, R. A. Chemical Actinometry: Using o-Nitrobenzaldehyde to Measure Lamp Intensity in Photochemical Experiments. *J. Chem. Educ.* **2000**, 77, 900–902.

(3) (a) APEX3 v2016.9-0 (SAINT), Bruker AXS Inc., Madison, WI, USA, **2016**; (b) Krause, L.; Herbst-Irmer, R.; Sheldrick, G. M.; Stalke, D. Comparison of Silver and Molybdenum Microfocus X-ray Sources for Single-Crystal Structure Determination. *J. Appl. Cryst.* **2015**, *48*, 3–10; (c) Sheldrick, G. M. SHELXT - Integrated Space-Group and Crystal-Structure Determination. *Acta Cryst.* **2015**, *A71*, 3–8; (d) Sheldrick, G. M. Crystal Structure Refinement with SHELXL. *Acta Cryst.* **2015**, *C71*, 3–8.

(4) Sheldrick, G. M. A Short History of *SHELX*. *Acta Cryst.* **2008**, *A64*, 112−122.

(5) Dolomanov, O. V.; Bourhis, L. J.; Gildea, R. J.; Howard, J. A. K.; Puschmann, H. *OLEX2*: A Complete Structure Solution, Refinement and Analysis Program. *J. Appl. Cryst.* **2009**, *42*, 339−341.

(6) Hübschle, Ch. B.; Sheldrick, G. M.; Dittrich, B. ShelXle: a Qt Graphical User Interface for SHELXL. *J. Appl. Cryst.,* **2011**, *44*, 1281−1284.

(7) (a) Neese, F. The ORCA Program System. WIREs *Comput. Mol. Sci.* **2012**, *2*, 73–78; (b) Neese, F.; Wennmohs, F.; Becker, U.; Riplinger, C. The ORCA Quantum Chemistry Program Package. *J. Chem. Phys.* **2020**, *152*, 224108; (c) Neese, F. Software Update: The ORCA Program System—Version 5.0. WIREs *Comput. Mol. Sci.* **2022**, *12*, e1606.

(8) Grimme, S.; Hansen, A.; Ehlert, S.; Mewes, J.-M. r2SCAN-3c: A “Swiss Army Knife” Composite Electronic-Structure Method. *J. Chem. Phys.* **2021**, *154*, 064103.

(9) Andrae, D.; Haeussermann, U.; Dolg, M.; Stoll, H.; Preuss, H. Energy-Adjustedab Initio Pseudopotentials for the Second and Third Row Transition Elements. *Theor. Chim. Acta* **1990**, *77*, 123–141.

(10) (a) Whitten, J. L. Coulombic Potential Energy Integrals and Approximations. *J. Chem. Phys*. **1973**, *58*, 4496–4501; (b) Dunlap B. I.; Connolly, J. W. D.; Sabin, J. R. On some Approximations in Applications of Xα Theory. *J. Chem. Phys*. **1979**, *71*, 3396–3402; (c) Neese, F. An Improvement of the Resolution of the Identity Approximation for the Formation of the Coulomb Matrix. *J. Comput. Chem*. **2003**, *24*, 1740–1747.

(11) Schwabe, T.; Grimme, S. Towards Chemical Accuracy for the Thermodynamics of Large

Molecules: New Hybrid Density Functionals Including Non-Local Correlation Effects. *Phys. Chem. Chem. Phys.* **2006**, *8*, 4398–4401.

(12) Weigend F.; Ahlrichsb, R. Balanced Basis Sets of Split Valence, Triple zeta Valence and Quadruple zeta Valence Quality for H to Rn: Design and Assessment of Accuracy. *Phys. Chem. Chem. Phys.* **2005**, *7*, 3297–3305.

(13) Caldeweyher E.; Bannwarth, C.; Grimme, S. Extension of the D3 Dispersion Coefficient Model. *J. Chem. Phys.* **2017**, *147*, 034112.

(14) Neese, F.; Wennmohs, F.; Hansen, A.; Becker, U. Efficient, Approximate and Parallel Hartree–Fock and Hybrid DFT Calculations. A 'Chain-of-Spheres' Algorithm for the Hartree–Fock Exchange. *Chem. Phys.* **2009**, *356*, 98–109.

(15) Weigend, F. Accurate Coulomb-Fitting Basis Sets for H to Rn. *Phys. Chem. Chem. Phys.* **2006**, *8*, 1057–1065.

(16) (a) Hättig, C. Optimization of Auxiliary Basis Sets for RI-MP2 and RI-CC2 Calculations: Core–Valence and Quintuple-ζ Basis Sets for H to Ar and QZVPP Basis Sets for Li to Kr. *Phys. Chem. Chem. Phys.* **2005**, *7*, 59–66; (b) Hellweg, A.; Hättig, C.; Höfener, S.; Klopper, W. Optimized Accurate Auxiliary Basis Sets for RI-MP2 and RI-CC2 Calculations for the Atoms Rb to Rn. *Theor. Chem. Acc.* **2007**, *117*, 587–597.

(17) (a) Becke, A. D. Density-Functional Exchange-Energy Approximation with Correct Asymptotic Behavior. *Phys. Rev. A*. **1988**, *38*, 3098–3100; (b) Perdew, J. P.; Wang, Y. Accurate and Simple Density Functional for the Electronic Exchange Energy: Generalized Gradient Approximation. *Phys. Rev. B* **1986**, *33*, 8800.

(18) Barone, V.; Cossi, M. Quantum Calculation of Molecular Energies and Energy Gradients in Solution by a Conductor Solvent Model. *J. Phys. Chem. A* **1998**, *102*, 1995–2001.

(19) Stephens, P. J.; Devlin, F. J.; Chabalowski, C.F.; Frisch, M. J. Ab Initio Calculation of Vibrational Absorption and Circular Dichroism Spectra Using Density Functional Force Fields. *J. Phys. Chem.* **1994**, *98*, 11623–11627.

(20) (a) Glendening, E. D.; Badenhoop, J. K.; Reed, A. E.; Carpenter, J. E.; Bohmann, J. A.; Morales, C. M.; Karafiloglou, P.; Landis, C. R.; Weinhold, F. NBO 7.0.; *Theoretical Chemistry Institute*; University of Wisconsin: Madison, **2018**; (b) Glendening, E. D.; Landis, C. R.; Weinhold, F. NBO 7.0: New Vistas in Localized and Delocalized Chemical Bonding Theory. *J. Comput. Chem.* **2019**, *40*, 2234–2241.

(21) Mitoraj, M.; Michalak, A. Natural Orbitals for Chemical Valence as Descriptors of Chemical Bonding in Transition Metal Complexes. *J. Mol. Model.* **2007**, *13*, 347–355.

(22) a) Lu, T.; Chen, F. Multiwfn: A Multifunctional Wavefunction Analyzer. *J. Comput.*

Chem. **2012**, *33*, 580–592; (b) Lu, T. A Comprehensive Electron Wavefunction Analysis Toolbox for Chemists, Multiwfn. *J. Chem. Phys.* **2024**, *161*, 082503.

(23) (a) Lenthe, E. V.; Baerends, E. J.; Snijders, J. G. Relativistic Regular Two-Component Hamiltonians. *J. Chem. Phys.* **1993**, *99*, 4597–4610; (b) Van Lenthe, E.; Baerends, E. J.; Snijders, J. G. Relativistic Total Energy Using Regular Approximations. *J. Chem. Phys.* **1994**, *101*, 9783–9792; (c) vanLenthe, E.; Snijders, J. G.; Baerends, E. J. The Zero-Order Regular Approximation for Relativistic Effects: The Effect of Spin-Orbit Coupling in Closed Shell Molecules. *J. Chem. Phys.* **1996**, *105*, 6505–6516; (d) van Wüllen, C. Molecular Density Functional Calculations in the Regular Relativistic Approximation: Method, Application to Coinage Metal Diatomics, Hydrides, Fluorides and Chlorides, and Comparison with First-Order Relativistic Calculations. *J. Chem. Phys.* **1998**, *109*, 392–399.

(24) Pantazis, D. A.; Chen, X. Y.; Landis, C. R.; Neese, F. All-Electron Scalar Relativistic Basis Sets for Third-Row Transition Metal Atoms. *J. Chem. Theory Comput.* **2008**, *4*, 908–919.

(25) (a) Grimme, S.; Antony, J.; Ehrlich, S.; Krieg, H. A Consistent and Accurate ab Initio Parametrization of Density Functional Dispersion Correction (DFT-D) for the 94 Elements H-Pu. *J. Chem. Phys.* **2010**, *132*, 154104; (b) Grimme, S.; Ehrlich, S.; Goerigk, L. Effect of the Damping Function in Dispersion Corrected Density Functional Theory. *J. Comput. Chem.* **2011**, *32*, 1456–1465; (c) Becke, A. D.; Johnson, E. R. A Density-Functional Model of the Dispersion Interaction. *J. Chem. Phys.* **2005**, *123*, 154101.

(26) (a) Svensson, M.; Humbel, S.; Froese, R. D. J.; Matsubara, T.; Sieber, S.; Morokuma, K. ONIOM: A Multilayered Integrated MO + MM Method for Geometry Optimizations and Single Point Energy Predictions. A Test for Diels–Alder Reactions and $Pt(P(t\text{-}Bu)_3)_2$ + H_2 Oxidative Addition. *J. Phys. Chem.* **1996**, *100*, 19357–19363; (b) Svensson, M.; Humbel, S.; Morokuma, K. Energetics Using the Single Point IMOMO (Integrated Molecular Orbital Plus Molecular Orbital) Calculations: Choices of Computational Levels and Model System. *J. Chem. Phys.* **1996**, *105*, 3654–3661.

(27) (a) Werner, H.-J.; Knowles, P. J.; Knizia, G.; Manby, F. R.; Schütz, M. Molpro: A General-Purpose Quantum Chemistry Program Package. *Wiley Interdiscip. Rev. Comput. Mol. Sci.* **2012**, *2*, 242–253; (b) Werner, H.-J.; Knowles, P. J.; Manby, F. R.; Black, J. A.; Doll, K.; Heßelmann, A.; Kats, D.; Köhn, A.; Korona, T.; Kreplin, D. A.; Ma, Q.; Miller, T. F.; Mitrushchenkov, A.; Peterson, K. A.; Polyak, I.; Rauhut, G.; Sibaev, M. The Molpro Quantum Chemistry Package. *J. Chem. Phys.* **2020**, *152*, 144107; (c) Werner, H. -J.; Knowles, P. J. and others, MOLPRO, Version 2024, a package of ab initio programs.

(28) Adler, T. B.; Knizia, G.; Werner, H.-J. A simple and efficient CCSD (T)-F12

approximation. *J. Chem. Phys.* **2007**, *127*, 221106.

(29) Knizia, G.; Adler, T. B.; Werner, H.-J. Simplified CCSD (T)-F12 methods: Theory and benchmarks. *J. Chem. Phys.* **2009**, *130*, 054104.

(30) Peterson, K. A.; Adler, T. B.; Werner, H.-J. Systematically Convergent Basis Sets for Explicitly Correlated Wavefunctions: The Atoms H, He, B–Ne, and Al–Ar. *J. Chem. Phys.* **2008**, *128*, 084102.

(31) Figgen, D.; Peterson, K. A.; Dolg, M.; Stoll, H. Energy-Consistent Pseudopotentials and Correlation Consistent Basis Sets for the 5d Elements Hf–Pt. *J. Chem. Phys.* **2009**, *130*, 164108.

(32) (a) Yousaf, K. E.; Peterson, K. A. Optimized Auxiliary Basis Sets for Explicitly Correlated Methods. *J. Chem. Phys.* **2008**, *129*, 184108; (b) Kritikou, S.; Hill, J. G. Auxiliary Basis Sets for Density Fitting in Explicitly Correlated Calculations: The Atoms H-Ar. *J. Chem. Theory Comput.* **2015**, *11*, 5269–5276; (c) Weigend, F. Hartree–Fock Exchange Fitting Basis Sets for H to Rn. *J. Comput. Chem.* **2008**, *29*, 167–175; (d) Hill, J. G. Auxiliary Basis Sets for Density Fitting Second-Order Møller-Plesset Perturbation Theory: Correlation Consistent Basis Sets for the 5d Elements Hf-Pt. *J. Chem. Phys.* **2011**, *135*, 044105.

(33) (a) Riplinger, C.; Neese, F. An Efficient and Near Linear Scaling Pair Natural Orbital Based Local Coupled Cluster Method. *J. Chem. Phys.* **2013**, *138*, 034106; (b) Guo, Y.; Riplinger, C.; Becker, U.; Liakos, D. G.; Minenkov, Y.; Cavallo, L.; Neese, F. Communication: An Improved Linear Scaling Perturbative Triples Correction for the Domain Based Local Pair-Natural Orbital Based Singles and Doubles Coupled Cluster Method [DLPNO-CCSD(T)]. *J. Chem. Phys.* **2018**, *148*, 011101.

(34) (a) Dunning, T. H. Gaussian Basis Sets for Use in Correlated Molecular Calculations. I. The Atoms Boron Through Neon and Hydrogen. *J. Chem. Phys.* **1989**, *90*, 1007–1023; (b) Kendall, R. A.; Dunning, T. H.; Harrison, R. J. Electron Affinities of the First-Row Atoms Revisited. Systematic Basis Sets and Wave Functions. *J. Chem. Phys.* **1992**, *96*, 6796–6806; (c) Woon, D. E.; Dunning, T. H., Jr. Gaussian Basis Sets for Use in Correlated Molecular Calculations. III. The Atoms Aluminum through Argon. *J. Chem. Phys.* **1993**, *98*, 1358–1371.

(35) Weigend, F.; Köhn, A.; Hättig, C. Efficient Use of the Correlation Consistent Basis Sets in Resolution of the Identity MP2 Calculations. *J. Chem. Phys.* **2002**, *116*, 3175–3183.

(36) Figgen, D.; Peterson, K. A.; Dolg, M.; Stoll, H. Energy-Consistent Pseudopotentials and Correlation Consistent Basis Sets for the 5d Elements Hf–Pt. *J. Chem. Phys.* **2009**, *130*, 164108.

(37) Altun, A.; Neese, F.; Bistoni, G. Extrapolation to the Limit of a Complete Pair Natural Orbital Space in Local Coupled-Cluster Calculations. *J. Chem. Theory Comput.* **2020**, *16*, 6142–6149.

(38) (a) Klopper, W.; Kutzelnigg, W. Gaussian Basis Sets and the Nuclear Cusp Problem. *J. Mol. Struct.* **1986**, *135*, 339–356; (b) Kutzelnigg, W. Theory of the Expansion of Wave Functions in a Gaussian Basis. *Int. J. Quantum Chem.* **1994**, *51*, 447–463.

(39) Truhlar, D. G. Basis-Set Extrapolation. *Chem. Phys. Lett.***1998**, *294*, 45–48.

(40) Neese, F.; Valeev, E. F. Revisiting the Atomic Natural Orbital Approach for Basis Sets: Robust Systematic Basis Sets for Explicitly Correlated and Conventional Correlated ab Initio Methods? *J. Chem. Theory Comput.* **2011**, *7*, 33–43.

(41) (a) Bickelhaupt, F. M.; Fonseca Guerra, C.; Mitoraj, M.; Sagan, F.; Michalak, A.; Pan, S.; Frenking, G. Clarifying Notes on the Bonding Analysis Adopted by the Energy Decomposition Analysis. *Phys. Chem. Chem. Phys.* **2022**, *24*, 15726–15735; (b) Li, L.; Parr, R. G. The Atom in a Molecule: A Density Matrix Approach. *J. Chem. Phys*. **1986**, *84*, 1704–1711.
